# Cost-Effectiveness of Chimeric Antigen Receptor (CAR) T-Cell Therapy for Blood Cancers: An Updated Systematic Review

**DOI:** 10.1007/s41669-025-00614-x

**Published:** 2025-12-15

**Authors:** Nishma Patel, Suzanne Farid, Manuel Gomes

**Affiliations:** 1 Department of Primary Care and Population Health, University College London, Gower St, London, WC1E 6BT UK; 2 Department of Biochemical Engineering, University College London, Gower St, London, WC1E 6BT UK

## Abstract

**Background:**

Chimeric antigen receptor (CAR) T-cell therapy is an area of rapid development, showing the promise of curing blood cancers. While substantial health gains may justify high costs, it is currently unclear the extent to which the overall cost effectiveness of these therapies is driven by i) context-specific factors, such willingness-to-pay thresholds and study perspective, or ii) important subgroups such as line of treatment and therapy product.

**Objective:**

This paper aims to critically review published evidence on the cost effectiveness of CAR T-cell therapies and assess the key factors that drive their cost effectiveness.

**Methods:**

We conducted a systematic review using PubMed, Scopus and Ovid (Embase) databases to identify full economic evaluations of CAR T-cell therapies published up to January 2024. One reviewer screened and extracted data from the studies and the second reviewer assessed a sample of the full-text studies against the inclusion/exclusion criteria. Studies were critically appraised using the CHEERS checklist. Cost data are presented in 2022 US dollars.

**Results:**

The review identified 45 full cost-effectiveness studies of CAR T-cell therapies. These studies considered a total of 92 treatment comparisons, which included tisagenlecleucel (*n* = 37), axicabtagene ciloleucel (*n* = 28), brexucabtagene autoleucel (*n* = 7), lisocabtagene maraleucel (*n* = 8), idecabtagene vicleucel (*n* = 6), ciltacabtagene autoleucel (*n* = 4) and relmacabtagene autoleucel (*n* = 2). Incremental cost ranged from − US$74,980 to US$714,178 and incremental quality-adjusted life year (QALY) gains ranged from − 0.02 to 10.77. The resulting cost-per-QALY-gained ratios ranged from − US$37,490,000 to US$7,972,845, and the range of willingness-to-pay (WTP) thresholds between US$36,184 to US$317,825. The price of CAR T-cell therapy represented 75% (mean US$391,060) of the total cost of CAR T-cell therapy but was not the sole factor influencing cost effectiveness. Hospitalisation made up 6% of the total cost (mean US$34,152), while adverse events accounted for 9% (mean US$47,350). Regression analysis indicated cost effectiveness did not change according to important clinical or contextual factors.

**Conclusions:**

The findings demonstrate that the cost effectiveness of CAR T-cell therapies is determined by a combination of factors: the relative difference between the cost of the CAR T-cell therapy and comparator, the magnitude of the QALY gains and the WTP thresholds. Their cost- effectiveness does not differ according to therapy product, line of treatment, or country.

**Supplementary Information:**

The online version contains supplementary material available at 10.1007/s41669-025-00614-x.

## Key Points for Decision Makers

**Table:** 

Costs associated with chimeric antigen receptor (CAR) T-cell therapy are high but yield substantial incremental quality-adjusted life years (QALY) compared with standard care.
Key drivers of cost effectiveness are not limited to the price of drug acquisition but also costs associated with hospitalisation and adverse events.
There is a statistically non-significant relationship between the treatment line and cost effectiveness.

## Introduction

Hematologic cancers such as such as Hodgkin's and non-Hodgkin lymphoma, leukaemia, and multiple myeloma pose a health burden for the National Health Service (NHS) and affect 250,000 adults and children each year [1], with an estimated cost of US$2,281,447 per annum in the UK [2, 3]. Traditional treatments for blood cancers have been chemotherapy, radiotherapy and stem cell transplantation, which have shown improved 5-year survival rates [4], but at an increased risk of cardiovascular diseases (CVD) and long-term treatment-related morbidity [5]. Success of the revolutionary, one-time, autologous chimeric antigen receptor T-cell (CAR T-cell) therapy gives promise for potential cure for chronic, debilitating, and life-threatening blood cancers, but entails complex research, development, manufacturing and delivery [6].

Since 2017, regulatory agencies, such as the US Food and Drug Administration (FDA) and European Medicines Agency (EMA) have approved six cell-based therapies [7–14]:

1. tisagenlecleucel (Kymriah^®^) for the treatment of paediatric and young adult patients up to 25 years of age with B-cell acute lymphoblastic leukaemia (ALL) that are refractory, in relapse post-transplant or in second or later relapse as well as for adult patients with relapsed or refractory diffuse large B-cell lymphoma (DLBCL) after two or more lines of systemic therapy and follicular lymphoma;
2. axicabtagene ciloleucel (Yescarta^®^) for the treatment of adult patients with relapsed or refractory DLBCL and primary mediastinal large B-cell lymphoma (PMBCL) after two or more lines of systemic therapy;
3. brexucabtagene autoleucel (Tecartus^®^) for treating relapsed or refractory B-cell ALL in people aged 26 years and above;
4. lisocabtagene maraleucel (Breyanzi^®^) for the treatment of adult patients with relapsed or refractory (DLBCL), PMBCL and follicular lymphoma, after two or more lines of systemic therapy;
5. idecabtagene vicleucel (Abecma^®^) for treatment of adult patients with relapsed or refractory multiple myeloma after four or more prior lines of therapy;
6. ciltacabtagene autoleucel (Carvykti^®^) for treatment of adults with relapsed or refractory multiple myeloma after three lines of systemic therapy.

More recently, the Chinese National Medical Products Administration (NMPA) has granted approval for relmacabtagene autoleucel (Carteyva) [15, 16] for treatment in adults with multiple myeloma and DLBCL.

Early reviews on cost-effectiveness studies of advanced therapeutic medicinal products (ATMPs) include gene therapies alongside cell therapies and evaluate the challenges in economic evaluation of ATMPs. Lloyd-Williams and Hughes [17] reported on 23 studies, and highlight the lack of data on health-related quality of life/utilities, small size of clinical trials and the challenge this presents, alongside assumptions about efficacy and comparative effectiveness. Pinho-Gomes and Cairns [18] reviewed the methodological challenges of ATMPs by the UK National Institute for Health and Care Excellence (NICE) and concluded the need for new methods of appraisal to address uncertainty, given high upfront costs and unknown long-term benefits. Ho et al. [19] highlighted the importance of long-term efficacy and choice of comparators, model parameters and assumptions. More recent ATMP reviews [18–22] reported cost-effectiveness results on FDA-approved cell and gene therapies, suggesting long-term value despite high upfront costs, though there is uncertainty due to lack of long-term data [21]. Building on previous work by Lloyd-Williams and Hughes [17], de Labry-Lima et al. [20] reviewed economic analyses of ATMPs, concluding the need to align clinical trial design with Health Technology Assessment (HTA) requirements. One review study reported the lack of adherence to recommendations for cell and gene therapies [22].

Reviews solely on CAR T-cell therapies [23, 24] summarised the economic evidence of CAR T-cell therapies up to 2022, indicating CAR T-cell therapies are cost effective. A more recent review by Thavorn et al. [25] reviewed cost-effectiveness evidence on the use of CAR T-cell therapy in hematologic and solid malignancies, suggesting cost effectiveness is embedded with uncertainty and influenced by patient characteristics, type of cancer and the model assumptions.

Despite emerging evidence, there is uncertainty and a lack of clarity about the value for money of CAR T-cell therapies and how different countries should respond based on increasing healthcare costs and limited budgets. There is ambiguity in the factors driving the cost effectiveness of CAR T-cell therapies across different countries and concerns about the sustainability and affordability of cell therapies to healthcare systems.

This paper provides an up-to-date review of the published evidence on the cost effectiveness of CAR T-cell therapies and assesses the key factors that drive their cost effectiveness.

## Methods

### Search Strategy

The protocol for this systematic review was not registered with PROSPERO or any other systematic review registry. The PRISMA guidelines for systematic reviews were followed [26].

The aim of the search strategy was to identify full economic evaluations of CAR T-cell therapies which have received market authorisation between 1 January 2017 and 31 January 2024. Searches were conducted within this same period to ensure consistency. The search was performed using three databases: PubMed, Scopus and Ovid (Embase). The search strategy combined CAR T-cell therapies and economics evaluation-related search terms. A full list of search terms is reported in the electronic supplementary material (ESM, Table S1). For example, the following medical subject headings (MeSH) from PubMed were applied to identify relevant peer-reviewed studies: ‘Cell- and Tissue-Based Therapy’ OR ‘Receptors, Chimeric Antigen’ OR ‘Genetic Therapy’ OR ‘Antigens, CD19’ OR ‘Precision Medicine’ OR ‘Regenerative Medicine’ AND ‘Technology Assessment, Biomedical’ OR ‘Cost-Benefit Analysis’. To complement the MeSH search, we have also conducted a free-text search (non-MeSH terms) using Ovid and Scopus. Free-text search terms used to initiate the search were ‘Tisagenlecleucel OR Kymriah OR Axicabtagene ciloleucel OR Yescarta OR Brexucabtagene autoleucel OR Tecartus OR Lisocabtagene maraleucel OR Breyanzi OR Idecabtagene vicleucel OR Abecma OR Ciltacabtagene autoleucel OR Carvykti OR Relmacabtagene Autoleucel OR Carteyva AND Economic Evaluation OR Decision Modelling OR Budget impact OR Cost Utility Analysis OR Cost Effectiveness Analysis OR Cost Benefit Analysis OR Health Technology Assessment OR Value for money’. All free-text search terms were agreed with the second reviewer (MG).

### Inclusion and Exclusion Criteria

Studies were included if they were full cost-effectiveness (CEA), cost-benefit (CBA) or cost-utility (CUA) studies, comparing both the costs and health effects of a CAR T-cell therapy with alternative interventions. Conference abstracts, unavailable full-text, commentaries, editorials, cost-only analysis, reviews, budget impact analysis, partial economic evaluations (e.g., cost-minimisation analysis), economic reports with redacted information (reimbursement agencies’ appraisals), guidance and non-English studies were excluded.

### Data Extraction

All searches were downloaded into a reference manager library (EndNote). After duplicates were removed, titles and abstracts were reviewed by the first reviewer 1 (NP) to determine whether each study met the eligibility criteria. Abstracts were screened by the first reviewer (NP), followed by retrieval of full-text copies of relevant studies. A sample (10%) of the full-text studies were independently considered by the second reviewer (MG) against the inclusion/exclusion criteria. At the end of the full-text review, NP and MG ensured that all the selected studies met the predefined inclusion criteria and NP extracted the data. Any disagreements were addressed through discussion; no third reviewer was required to resolve these. All cost data were converted to 2022 US dollars using OECD purchasing power parity (PPP) adjustments.

### Narrative Synthesis

Data were extracted from the included studies, summarising key study characteristics (i.e. country, cost of CAR T-cell therapy perspective, population, discount rate and time horizon). For cost-effectiveness results, the denominator was the total number of comparisons across all cost-effectiveness studies (Table 2). There are currently no studies summarising the cost effectiveness of CAR T-cell therapies by treatment line.

### Regression Analysis

We conducted a multiple linear regression analysis to assess whether cost effectiveness was associated with various clinical and contextual factors: (i) type of CAR T-cell therapy, (ii) price of CAR T-cell therapy, (iii) treatment line, (iv) country, (v) funding source, (vi) type of cancer, (vii) population and (viii) maturity of the efficacy evidence (overall survival). We specifically examined the impact of the maturity of efficacy evidence by extracting the maturity of Kaplan–Meier (KM) curves for overall survival, which reflected the follow-up duration from the randomised controlled trials (RCTs) informing the efficacy endpoints. Based on this, we created a categorical variable representing evidence maturity: (i) up to 2 years, (ii) between 2 and 3 years, and (iii) over 3 years. This variable was included in the regression analyses. The regression included comparisons with a payer perspective, excluded head-to-head CAR T-cell therapy comparisons, and included the specified lines of treatment (*n* = 52). Regression 1 used the line of treatment classifications reported in the paper (i.e., 2L, ≥2L, 3L, ≥3L, and ≥4L). In contrast, regression 2 (adjusted analysis) simplified the treatment line categories to 2, 3, or 4.

### Assessment of Study Reporting Quality

The updated Consolidated Health Economic Evaluation Reporting Standards (CHEERS 2022) checklist [70, 71] was used to assess the reporting quality of each study included in the review (ESM, Table S6). Contents of each paper were checked against the checklist, indicating the section where the relevant information was available in the paper.

## Results

**Fig. 1:**
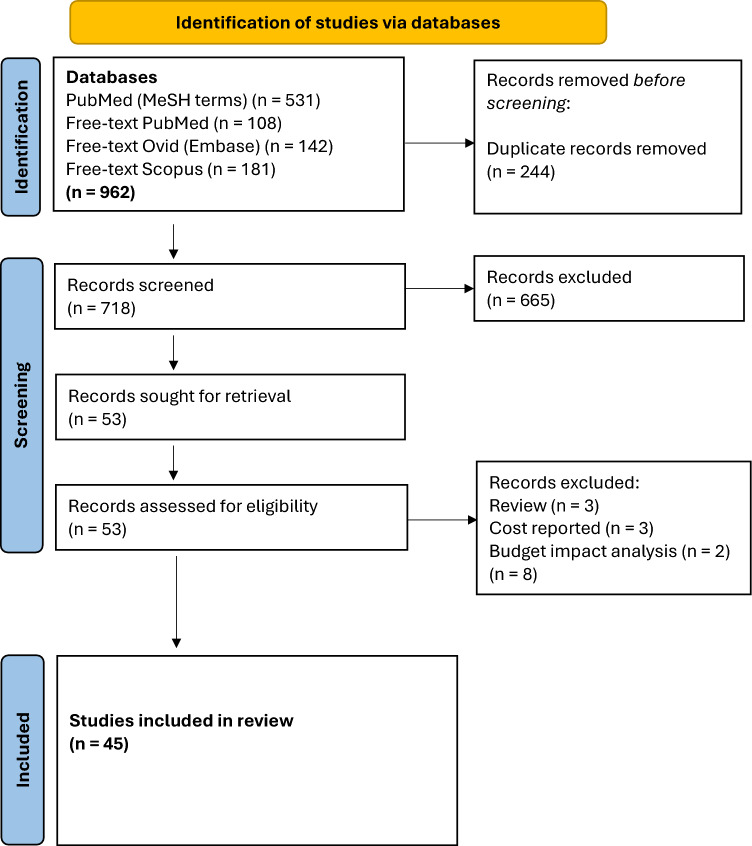
PRISMA flow diagram

The full results of the selection process are shown in Fig. 1. The search yielded 962 studies, of which 244 were duplicates. A total of 53 potentially relevant studies met the eligibility criteria and were shortlisted for full-text screening. After full-text screening, a further 8 studies were excluded: literature review (*n* = 3), cost study (*n* = 3) and budget impact analysis (*n* = 2). A total of 45 studies were included in the review.

### Characteristics of Studies

Study characteristics were extracted and are summarised in Table 1. The 45 studies were conducted across several countries. The vast majority of the studies reported cost-effectiveness results for a single country, with the exception of two studies that reported cost-effectiveness results for two countries [65, 68]. Studies were categorised by country and more than half of the studies were from the US (*n* = 23; 51%), followed by Canada (*n* = 4; 9%), China (*n* = 4; 9%), Singapore (*n* = 3; 7%) and Japan (*n* = 3; 7%) (Table 1). Two main CAR T-cell therapies were considered in several studies, tisagenlecleucel (*n* = 18; 40%) [28, 43–56] and axicabtagene ciloleucel (*n* = 18; 40%) [16, 27–29, 31–42, 66, 67]. Most common blood cancers treated with CAR T-cell therapies were (i) adult, large B-cell lymphoma (*n* = 12; 27%), (ii) adult, DLBCL (*n* = 11; 24%) and (iii) paediatric, B-cell ALL (*n* = 8; 18%) (Table 1). Five studies (*n* = 5; 11%) [31, 33, 34, 37, 62] considered head-to-head comparisons of CAR T-cell therapies, of which one study [62] considered two head-to-head comparisons.

**Table 1:** Study characteristics

Author (year)	Country	Population/indication	Treatment line	Type of model	Perspective
Roth et al. (2018) [27]	United States	Adult, r/r large B-cell lymphoma	Not specified	TSPS	Public payer
Lin et al. (2018) [28]	United States	Paediatric, r/r B-cell ALL	Not specified	Markov	Public payer
Whittington et al. (2019) [29]	United States	Adult, r/r B-cell lymphoma	Not specified	TSPS + DT	Public payer
		Adult, r/r B-cell lymphoma	Not specified	TSPS + DT	Commercial payer
Lin et al. (2019) [30]	United States	Adult, DLBCL	Not specified	Markov	Public payer
Liu et al. (2021) [31]	United States	Adult, r/r large B-cell lymphoma	≥ 2L	TSPS	Public payer
Perales et al. (2022) [32]	United States	Adult, large B-cell lymphoma	2L	TSPS	Commercial payer
Oluwole et al. (2022) [33]	United States	Adult, r/r large B-cell lymphoma	≥ 2L	TSPS	Public payer
Cummings et al. (2022) [34]	United States	Adult, r/r large B-cell lymphoma	≥ 2L	DT	Public payer
Kambhampati et al. (2022) [35]	United States	Adult, r/r DLBCL	2L	Markov	Public payer
Hillis et al. (2022) [36]	Canada	Adult, r/r large B-cell lymphoma	≥ 2L	TSPS	Public payer
		Adult, r/r large B-cell lymphoma		TSPS	Societal
Bastos-Oreiro (2022) [37]	Spain	Adult, r/r DLBCL	≥ 2L	TSPS	Public payer
Li et al. (2022) [38]	China	Adult, r/r DLBCL	≥ 2L	TSPS + DT	Public payer
Choe et al. (2022) [39]	United States	Adult, r/r large B-cell lymphoma	2L	TSPS	Public payer
				TSPS	Societal
Wu et al. (2023) [16]	China	Adult, r/r DLBCL	1L, 2L, ≥ 3L	Markov	Public payer
Potnis et al. (2023) [40]	United States	Adult, r/r follicular lymphoma	3L	Markov	Public payer
Loftager et al. (2023) [41]	Sweden	Adult, r/r large B-cell lymphoma	2L	TSPS	Public payer
Vijenthira et al. (2023) [42]	United States	Adult, r/r DLBCL	2L	Markov	Public payer
Whittington et al. (2018) [43]	United States	Paediatric, r/r B-cell ALL	Not specified	TSPS + DT	Public payer
Sarkar et al. (2019) [44]	United States	Paediatric, B-cell ALL	Not specified	Markov model	Third-party payer
				Markov model	Societal
Qi et al. (2021) [45]	United States	Adult, r/r DLBCL	≥ 2L	TSPS	Third-party payer
Furzer et al. (2020) [46]	Canada	Paediatric, B-cell ALL	2L	Microsimulation	Public payer
Santasusana et al. (2020) [47]	Spain	Paediatric, r/r B-cell ALL	Not specified	TSPS	Public payer
Wakase et al. (2021) [48]	Japan	Paediatric and young adult, r/r B-cell ALL	Not specified	TSPS + DT	Public payer
Wakase et al. (2021) [49]	Japan	Adult, r/r DLBCL	≥ 3L	TSPS + DT	Public payer
Thielen et al. (2020) [50]	Netherlands	Paediatric, r/r B-cell ALL	1L	TSPS	Public payer
				TSPS	Societal
Moradi-Lakeh et al. (2021) [51]	Switzerland	Paediatric ALL, Adult DLBCL	≥ 2	TSPS	Public payer
Cher et al. (2020) [52]	Singapore	Adult, r/r B-cell ALL	≥ 2L	TSPS + DT	Public payer
Wang et al. (2021) [53]	Singapore	Adult, r/r DLBCL	≥ 2L	TSPS	Private Payer
Wang et al. (2022) [54]	Singapore	Paediatric and young adult, r/r B-cell ALL	≥ 2L	TSPS + DT	Public payer
Carey et al. (2022) [55]	Ireland	Paediatric and young adult, B-cell ALL	≥ 2L	TSPS + DT	Public payer
Gye et al. (2022) [56]	Australia	Paediatric and young adult, B-cell ALL	Not specified	TSPS + DT	Public payer
Simons et al. (2021) [57]	United States	Adult, r/r MCL	≥ 2L	TSPS	Public payer
Ball et al. (2022) [58]	Canada	Adult, r/r MCL	Not specified	TSPS	Public payer
Shah et al. (2022) [59]	United States	Adult, r/r B-cell ALL	Not specified	TSPS + DT	Public payer
Petersohn et al. (2022) [60]	United Kingdom	Adult, r/r MCL	≥ 2L	TSPS	Public payer
Marchetti and Visco (2023) [61]	Italy	Adult, r/r MCL	Not specified	TSPS	Public payer
Parker et al. (2023) [62]	United States	Adult, r/r large B-cell lymphoma	≥ 3L	TSPS	Commercial payer
Kapinos et al. (2023) [63]	United States	Adult, r/r multiple myeloma	Not specified	Microsimulation	Public payer
Wu et al. (2023) [64]	China	Adult, r/r multiple myeloma	≥ 4L	Markov model	Public payer
Karampampa et al. (2023) [65]	Canada	Adult, r/r multiple myeloma	≥ 3L	TSPS	Societal
	France	Adult, r/r multiple myeloma	≥ 3L	TSPS	Societal
Lin et al. (2023) [15]	China	Adult r/r large B-cell lymphoma	2L	TSPS + DT	Public payer
Kelkar et al. (2023) [66]	United States	Adult, DLBCL	2L	Microsimulation	Public payer
Oluwole et al. (2024) [67]	United States	Adult, large B-cell lymphoma	2L	TSPS	Third-party payer
Yamamoto et al. (2024) [68]	Japan	Adult, r/r multiple myeloma	≥ 3L	Markov	Public payer
	United States	Adult, r/r multiple myeloma	≥ 3L	Markov	Public payer
Choe et al. (2024) [69]	United States	Adult, r/r DLBCL	2L	TSPS	Public payer
				TSPS	Societal

45 papers; two papers report cost-effectiveness results for two countries [65, 68]. Costs converted to 2022 US dollars using OECD purchasing power parity (PPP) adjustments

*ALL* acute lymphoblastic leukaemia, *DLBCL* diffuse large B-cell lymphoma, *MCL* mantle cell lymphoma, *r/r* relapsed/refractory, *TSPS* three-state partitioned survival model, *TSPS + DT* three-state partitioned survival model and decision tree,* DT* decision tree, *1L* first-line treatment, *2L* second-line treatment, *3L* third-line treatment

The public healthcare perspective was the most adopted perspective (*n* = 40; 89%) and four studies [36, 39, 50, 69] presented both public healthcare and societal perspectives (*n* = 4; 9%) (Table 1). Health-state utilities were derived from clinical trial data (*n* = 10; 22%) [15, 36, 41, 50, 57, 61, 62, 64, 65, 69] and published literature (*n* = 35; 78%) [16, 27, 29–35, 37–40, 42–49, 51–56, 58–60, 63, 66–68], using the EuroQol instruments EQ-5D-3L and EQ-5D-5L.

A lifetime horizon was applied in the majority of the studies, with the exception of two studies that used a 20-month [63] and a 10-year horizon [68], respectively. Discount rates ranged from 0.1% to 5%.

The included studies were either cost-effectiveness (CEA) or cost-utility (CUA) studies. The most common model structure employed was the three-state partitioned survival model (*n* = 32; 71%), often preferred in cancer areas [72]. Where studies were not conducted alongside CAR T-cell therapy clinical trials, 89% (*n* = 40 papers) of the studies [15, 16, 27–29, 31–41, 43, 45, 47–55, 57–63, 65–69] applied clinical efficacy and survival data from trial data to inform the models.

The results of the multiple linear regression analyses are reported in the ESM (Table S5). The regression models suggested 51% to 64% of the variability in cost per QALY gained was explained by the dependent variables. Irrespective of the model used in the regression, there was some (weak) evidence that long-term survival (over 3 years) was associated with a more favourable cost-effectiveness profile, reflected in a lower cost per QALY gained. None of the other factors appeared to drive the cost effectiveness of CAR-T therapies.

**Fig. 2:**
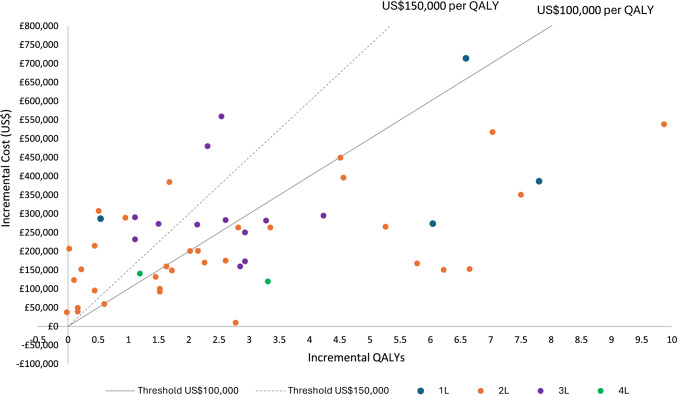
Incremental cost (US$) and QALYs by treatment line. *N* = 52 comparisons. Where two perspectives are given and one is societal, we took the payer perspective. Excludes CAR T vs CAR T comparisons and unspecified treatment line case. *CAR T* chimeric antigen receptor T-cell, *QALY* quality-adjusted life years, 1L = first line, 2L = second line, 3L = third line, 4L = fourth line

The analysis of incremental cost (US$) and QALYs by treatment line appeared to illustrate a nonlinear relationship at the WTP thresholds of US$100,000 and US$150,000 per QALY (Fig. 2).

### Variation in Cost and Utility Estimates

**Fig. 3:**
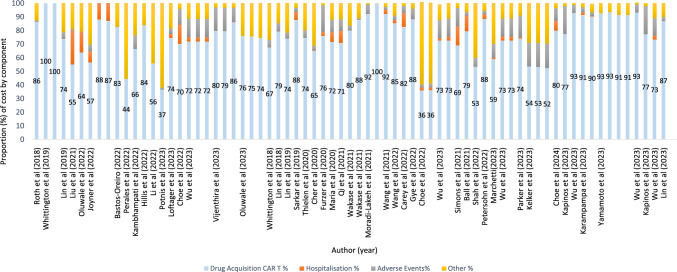
CAR T-cell therapy spending by cost component (%). *CAR T-cell therapy* chimeric antigen receptor T-cell therapy

The cost components included in the economic evaluation varied across studies (Fig. 3). To derive the mean for each cost category, cost data were extracted from individual studies and aggregated. All studies included the cost of drug acquisition and the vast majority (*n* = 42, 93%) reported the cost of adverse events, which accounted for approximately 9% (mean US$47,350) of the total cost [16, 35, 39, 42, 46, 57, 62, 63, 66]. The largest cost component of CAR T-cell therapies was the cost of the drug itself, which was responsible for 75% (mean US$391,060) of total costs. Hospitalisation accounted for 6% (mean US$34,152) of total costs. Several studies (*n* = 18; 41%) failed to disaggregate the cost of hospitalisation post-infusion and hospital readmissions due to adverse events. A breakdown of costs associated with adverse events is reported in the ESM, Table S3. One-time event costs, such as administration, monitoring, infusion and leukapheresis, hematologic stem cell transplantation (HSCT) events and chemotherapy costs were combined for the purpose of this review.

Lowest QALYs were reported for lisocabtagene maraleucel (1.41) in the USA [66], followed by idecabtagene vicleucel (1.81) in Japan [68]. In contrast, the highest gains were reported in the USA for tisagenlecleucel (16.76) [44] and axicabtagene ciloleucel (9.61) [42] (Table 2).

**Table 2:** Summary of cost-effectiveness results

Author (year)	CAR T-cell (US$)		Comparator		Intervention (US$)		Comparator (US$)		Δ US$		Intervention QALYs		Comparator QALYs		Δ QALYs		ICER (US$)	WTP (US$)	Cost-effective^a^ (%)
Axicabtagene ciloleucel																			
Roth et al. (2018) [27]	373,000		SC		552,921		172,737		380,184		7.67		1.13		6.54		58,146	100,000	90.0
Whittington et al. (2019) [29]	459,000		Chemo		459,700		108,600		351,100		2.07		0.55		1.52		896,600	NR	NR
	554,000		Chemo		554,000		114,500		439,500		2.07		0.55		1.52		1,615,000	NR	NR
Lin et al. (2019) [30, 31]	373,000		SC + SCT		651,000		169,000		482,000		5.5		1.78		3.72		129,000	150,000	73
Perales et al. (2022) [32]	399,000		SC		635,794		535,428		100,366		7.08		5.56		1.52		66,381	150,000	75.0
Kambhampati et al. (2022) [35]	393,104		SC		771,838		508,034		263,804		5.42		2.6		2.82		93,547	150,000	73
Hillis et al. (2022) [36]	395,936		Chemo		502,894		106,704		396,190		7.71		3.15		4.56		86,851	150,000	92
			Chemo		682,976		188,274		494,702		7.02		2.84		4.18		108,365	122,449	71
Li et al. (2022) [38]	173,978		SC		198,070		22,690		175,380		3.08		0.47		2.61		67,251	200,000	99
Potnis et al. (2023) [40]	443,118		SC		731,682		458,490		273,192		7.04		5.54		1.5		182,127	150,000	4
Loftager et al. (2023) [41]	386,242		Chemo		472,860		379,963		92,897		7.51		5.99		1.52		61,102	114,273	73
Choe et al. (2022) [39]	399,000		SC + HST		678,903		619,149		59,754		4.53		3.93		0.6		99,101	100,000	44
																		150,000	57
			SC + ASCT		688,507		629,431		59,076		4.55		3.94		0.6		97,977	NR	NR
Wu et al. (2023) [16]	298,359		SC		339,867		52,913		286,954		8.4		7.86		0.54		528,421	191,875	0
	298,359		SC + ASCT		329,737		197,849		131,889		5.03		3.58		1.45		90,497	191,875	0
	298,359		SC		345,696		63,758		281,939		5.39		2.11		3.28		86,029	191,875	0
Vijenthira et al. (2023) [42]	399,000		R-CHOP plus 2L SC ± ASCT		498,243		283,398		214,845		9.61		9.17		0.44		488,284	150,000	0
	399,000		R-CHOP plus 2L SC ± ASCT		332,968		283,398		49,570		9.33		9.17		0.16		309,813	150,000	0.1
	399,000		R-CHOP plus 2L SC ± ASCT		204,092		108,172		95,920		9.61		9.17		0.44		218,000	150,000	0
	399,000		R-CHOP plus 2L SC ± ASCT		147,769		108,172		39,597		9.33		9.17		0.16		247,480	150,000	0
Kelkar et al. (2023) [66]	417,735		SC + ASCT		537,361		385,260		152,101		1.82		1.6		0.22		684,225	200,000	20
Oluwole et al. (2024) [67]	462,000		SC + ASCT		769,890		609,981		159,909		7.23		5.6		1.63		98,040	150,000	82
Liu et al. (2021) [31]	373,000		Tis		586,313		587,720		− 1407		7.47		5.16		2.31		− 609	31,500	95.0
Oluwole et al. (2022) [33]	468,499		LM		611,440		597,174		14,266		7.76		5.94		1.82		7843	50,000	93.0
Cummings et al. (2022) [34]	399,000		LM		637,129		620,962		16,167		7.705		5.898		1.807		8946	150,000	100
	399,000		Tis		631,331		576,563		54,768		7.24		5.005		2.235		24,506	150,000	100
Bastos-Oreiro (2022) [37]	516,316		Tis		708,465		658,901		81,520		7.47		5.16		2.31		1003	36,184	92
Tisagenlecleucel																			
Whittington et al. (2018) [43]	405,490		Clo		666,754		337,256		329,498		9.28		2.1		7.18		45,891	NR	NR
Lin et al. (2018) [28]	475,000		Blin		599,000		282,000		317,000		8.74		3.57		5.17		61,315	100,000	98
	475,000		CC		599,000		374,000		225,000		8.74		3.52		5.22		43,520	NR	NR
	475,000		CM		599,000		314,000		285,000		8.74		3.12		5.62		55,125	NR	NR
Lin et al. (2019) [30]	373,000		SC + SCT		529,000		169,000		360,000		3.92		1.78		2.14		168,224	150,000	33
Sarkar et al. (2019) [44]	475,000		SC		528,200		440,600		87,600		16.76		8.58		8.18		64,601	100,000	94.8
																	69,500	100,000	NR
Qi et al. (2021) [45]	373,000		SC		588,080		324,319		263,761		5.29		1.94		3.35		78,652	150,000	91.9
Furzer et al. (2020) [46]	507,959		SC + ASCT		477,551		93,061		384,490		5.14		3.46		1.68		383,685	122,449	32.0
Santasusana et al. (2020) [47]	413,452		SC		587,870		162,906		424,964		9.43		0.46		8.97		47,400	NR	NR
Wakase et al. (2021) [48]	349,622		Blin		412,782		235,478		177,304		11.6		3.1		8.5		20,857	51,149	100.0
			CC		412,782		153,592		259,189		11.6		2.1		8.5		27,105	51,149	100.0
Wakase et al. (2021) [49]	349,622		SC		369,813		210,032		159,781		5.42		2.57		2.85		56,127	51,149	80.0
Thielen et al. (2020) [50]	418,848		Blin		536,077		262,164		273,914		11.26		2.25		9.01		30,404	NR	NR
	418,848		CM		536,077		149,132		386,945		11.26		0.49		10.77		35,920	NR	NR
	418,848		CC		536,077		178,101		714,178		11.26		1.7		9.56		37,449	NR	NR
	418,848		Blin		723,402		349,815		273,914		11.26		2.25		9.01		34,188	104,712	98.0
	418,848		CM		723,402		210,475		386,945		11.26		0.49		10.77		40,271	NR	NR
	418,848		CC		723,402		253,822		357,976		11.26		1.7		9.56		41,880	NR	NR
Moradi-Lakeh et al. (2021) [51]	253,012		CC (pALL)		340,838		188,008		152,830		8.29		1.64		6.65		22,989	107,581	pALL 100
			SC (pALL)		340,838		172,813		168,025		8.29		2.51		5.78		21,279	107,581	pALL 100
			Blin (pALL)		340,838		190,143		150,695		8.29		2.07		6.22		24,247	107,581	pALL 100
			SC (DLBCL)		268,622		98,292		170,328		4.77		2.51		2.26		75,352	66,578	DLBCL 86.6
Cher et al. (2020) [52]	370,370		SC		297,911		39,536		307,589		2.064		1.556		0.508		508,530	280,000	0.0
Wang et al. (2021) [53]	595,238		SC		616,252		626,343		10,090		5.6655		2.8905		2.775		− 10,090	317,825	100.0
Wang et al. (2022) [54]	595,238		SC		713,005		174,532		538,473		10.6		0.73		9.87		54,571	317,825	100.0
	595,239		Blin		713,005		249,101		350,885		10.6		3.1		7.5		61,879	317,825	100.0
Carey et al. (2022) [55]	387,371		Blin		483,797		282,349		201,448		4.33		2.18		2.15		93,820	57,766	16.0
Gye et al. (2022) [56]	385,667		Blin		585,890		145,380		267,510		5.36		1.09		4.27		62,705	NR	NR
Choe et al. (2022) [39]	373,000		SC + ASCT		534,426		496,623		37,803		2.02		2.04		− 0.02		− 130,355	100,000	9
																		150,000	15
	373,000		SC + ASCT		489,767		218,368		271,399		3.86		1.72		2.14		126,593	100,000	9
																		150,000	79
	373,000		SC + ASCT		543,578		504,098		39,480		2.02		2.04		− 0.02		− 136,138	NR	NR
	373,000		SC + ASCT		499,457		225,016		274,442		3.86		1.72		2.14		128,012	NR	NR
Wu et al. (2023) [16]	309,547		SC + ASCT		353,865		146,532		207,333		2.34		2.32		0.02		7,972,845	191,875	0
	309,547		SC		360,350		63,758		296,592		3.93		2.11		1.82		162,963	191,875	0
Brexucabtagene autoleucel																			
Simons et al. (2021) [57]	373,000		Cyto chemo, PI, IMD, Bcl-2, BTKI		693,832		574,263		119,569		7.39		3.65		3.74		31,985	100,000	94
Ball et al. (2022) [58]	304,490		BSC		570,777		53,753		517,841		8.34		1.31		7.03		72,247	81,633	82
Shah et al. (2022) [59]	399,000		Blin		776,320		725,407		50,913		5.95		4.92		1.03		20,843	150,000	78
	399,000		Ino		776,320		524,789		251,531		5.95		2.69		3.26		77,271	150,000	74
	399,000		SC		776,320		344,293		432,027		5.95		2.17		3.78		93,768	150,000	75
Petersohn et al. (2022) [60]	464,197		Cyto chemo, PI, IMD, Bcl-2		566,468		117,095		449,373		5.99		1.48		4.51		99,432	NR	NR
Marchetti and Visco (2023) [61]	600,000		R-BAC		685,672		124,025		561,647		6.4		1.2		5.2		107,997	£145,550	88
Lisocabtagene maraleucel																			
Wu et al. (2023) [16]	309,547		SC + ASCT		348,128		198,826		149,302		4.73		3.01		1.72		86,225	191,875	0
	309,547		SC		351,235		61,418		289,817		2.83		1.88		0.95		306,807	191,875	0
	309,547		SC		347,072		63,758		283,314		4.72		2.11		2.61		108,617	191,875	0
Kelkar et al. (2023) [66]	412,362		SC + ASCT		547,951		424,386		123,565		1.41		1.31		0.1		1,171,909	200,000	19
Choe et al. (2024) [69]	410,300		SCare chemo + ASCT		668,624		467,624		201,001		3.64		1.62		2.02		99,669	100,000	54
			SCare chemo + ASCT		882,475		744,914		137,560		3.64		1.62		2.02		68,212	100,000	84
Parker et al. (2023) [62]	410,300		AC		440,106		515,085		− 74,980		5.09		5.09		0.002		− 37,490,000	100,000	82
	410,300		Tis		440,106		372,180		67,926		5.09		3.07		2.02		33,627	100,000	96
Idecabtagene vicleucel																			
Kapinos et al. (2023) [63]	442,705		ADC		455,761		65,428		390,333		3.25		0.52		2.73		142,979	NR	NR
Wu et al. (2023) [64]	193,023		SC		217,205		76,512		140,693		2.11		0.92		1.19		118,229	37,653	0
Karampampa et al. (2023) [65]	444,898		Chemo		603,933		123,533		480,400		3.37		1.53		2.31		208,363	NR	NR
	509,772		Chemo		802,606		243,047		559,559		3.57		1.03		2.54		559,559	NR	NR
Yamamoto et al. (2024) [68]	334,598		Chemo		445,156		212,889		232,267		1.81		0.7		1.11		208,959	76,866	NR
	419,500		Chemo		599,699		308,831		290,868		1.81		0.7		1.11		261,678	150,000	NR
Ciltacabtagene autoleucel																			
Kapinos et al. (2023) [63]	465,000		ADC		477,980		65,428		412,552		4.29		0.52		3.77		109,497	123,618	50
Wu et al. (2023) [64]	193,023		SC		196,318		75,512		119,806		4.23		0.92		3.31		36,195	37,653	72
Yamamoto et al. (2024) [68]	334,598		Chemo		386,295		212,889		173,406		3.63		0.7		2.93		59,223	76,866	NR
	465,000		Chemo		559,330		308,831		250,499		3.63		0.7		2.93		85,553	150,000	NR
Relmacabtagene autoleucel																			
Wu et al. (2023) [16, 64]	320,736		SC		358,909		63,758		295,151		6.34		2.11		4.23		69,857	191,875	0
Lin et al. (2023) [15]	320,736		SC		400,022		134,624		265,398		6.67		1.41		5.26		50,506	60,400	74
Mean incremental QALYs															3.47				

*n* = 92 comparisons, including societal perspective

All costs converted to 2022 US dollars using OECD purchasing power parity (PPP) adjustments

Δ indicates difference, *AC* axicabtagene ciloleucel, *ASCT* autologous stem cell transplant, *BA* brexucabtagene autoleucel, *Blin* blinatumomab, *CA* ciltacabtagene autoleucel, *CC* clofarabine combination, *Chemo* chemotherapy, *Clo* clofarabine, *CM* clofarabine monotherapy, *CP* commercial payer, *Cyto* chemo (bendamustine), *DLBCL* diffuse large B-cell lymphoma, *HP* healthcare perspective, *HST* autologous and allogeneic stem cell transplant, *ICER* incremental cost-effectiveness ratio, *IMD* immunomodulatory drugs, *INO* Inotuzumab ozogamicin, *IV* idecabtagene vicleucel, *LM* lisocabtagene maraleucel, *NR* not reported, *pALL* paediatric acute lymphoblastic leukaemia, *PI* proteasome inhibitors (bortezomib), *Pol-R-CHP + 2L CAR-T* polatuzumab–rituximab, cyclophosphamide, doxorubicin and prednisone (R-CHP) plus second-line CAR-T for early relapse, *PP* public payer, *RA* relmacabtagene autoleucel, *R-BAC* rituximab, bendamustine, cytarabine, *R-CHOP + 2L CAR-T* rituximab, cyclophosphamide, doxorubicin, vincristine and prednisone plus second-line CAR-T for early relapse, *SC* salvage chemotherapy, *SCare chemo + ASCT* standard care chemotherapy and autologous stem cell transplant, *SCT* stem cell transplant, *SP* societal perspective, *Tis* tisagenlecleucel

^a^Probability (%) CAR T-cell therapy is cost effective at various willingness-to-pay (WTP) thresholds, representing the likelihood CAR T-cell therapy would be considered cost effective relative to the comparator

Incremental QALYs ranged widely from 0.002 [39] to 10.77 [50], primarily driven by survival. The lowest incremental QALY (0.002) was reported for lisocabtagene maraleucel in the USA [62]. The highest incremental QALYs were observed for tisagenlecleucel in the Netherlands (10.77) [50], Singapore (9.87) [54] and Spain (8.97) [47] for use in paediatric relapsed/refractory B-cell ALL. Notably, one study is the USA reported a loss in incremental QALYs for tisagenlecleucel (− 0.02) [39]. On average, CAR T-cell therapy led to 3.47 additional QALYs across 92 comparisons (Table 2).

### Comparative Cost-Effectiveness Results

Table 2 describes 92 treatment comparisons (some studies conducted multiple comparisons), made across the 45 economic evaluations. A large proportion (*n* = 60; 66%) of comparisons reported incremental cost-effectiveness ratio (ICERs) below US$100,000, and less than one third (*n* = 30; 33%) reported ICERs above US$150,000 (three comparisons did not report the ICER). The highest ICER (US$7,972,845 per QALY) was reported in China, for tisagenlecleucel [16], and the highest cost of CAR T-cell therapy was reported for brexucabtagene autoleucel (US$600,000) in Italy [61] (Table 2).

Almost three quarters of comparisons (*n* = 67; 74%) reported a probability of CAR T-cell therapy being cost effective above 70%, partly because many countries considered relatively high WTP thresholds (above US$100k). Given the large variation in incremental benefits in long-term survival, high total costs and varying WTP thresholds, 25 (27%) comparisons indicated CAR T-cell therapy was not cost effective [16, 38–40, 42, 52, 55, 63, 64, 66–68], 21 comparisons made inconclusive recommendations [28–30, 39, 43, 44, 46, 50, 60, 61, 65] and 46 (51%) CAR T-cell therapy comparisons suggested CAR T-cell therapy was cost effective [15, 27, 28, 31–37, 39, 41, 44, 45, 47–51, 53, 54, 56–60, 62–64, 68, 69]. Nineteen comparisons did not report WTP threshold [28, 29, 39, 43, 44, 47, 50, 56, 60, 63, 65], and hence it wasn’t possible to establish the extent to which the CAR T-therapy was cost effective in the given country.

Three comparisons showed adverse events accounted for 20% of total costs (ESM, Table S3) in comparisons for axicabtagene ciloleucel, idecabtagene vicleucel and ciltacabtagene autoleucel [46, 63], while 17 comparisons did not account for the cost of adverse events. We found 38 comparisons failed to factor in the cost of hospitalisations, while in one comparison, hospitalisation costs accounted for 25% of the total [31]. Further, insufficient consideration of adverse events and hospitalisations contributed to uncertainty in the long-term cost-effectiveness of CAR T-cell therapies.

### Key Drivers of Cost Effectiveness

The price of the CAR T-cell therapy was high across all comparisons, ranging between US$173,978 and US$600,000 (Table 2), and was not the sole driver of cost effectiveness. Cost effectiveness was also dependent on (i) how inexpensive the comparator was (US$22,690–US$659,000), (ii) whether the CAR-T delivered considerable incremental QALY gains (− 0.02 to 10.77), and (iii) the WTP threshold (US$36,184–US$317,825). Twenty-nine studies (*n* = 30; 67%) were funded by a pharmaceutical company and 25 (*n* = 25; 56%) reported a recommendation for the respective CAR T-cell therapy (ESM, Figure S2).

### Study Reporting Quality

Items least likely to be reported in the CHEERS (2022) checklist [70, 71] were inclusion of a health economic analysis plan (HEAPS—item 4), characterisation of heterogeneity (item 18) and distributional effects (item 19). Given the nature of the studies, there was no stakeholder engagement and items 21 and 25 were categorised as not applicable (N/A). Four CHEERS checklist categories were fulfilled by all studies: Title; Abstract; Introduction and Discussion. Quality assessment showed that analytics and assumptions (item 17) (82%), followed by currency, price date, and conversion (item 15) were least likely to be clearly reported (93%). Overall, the study reporting was of good quality according to the CHEERS checklist.

## Discussion

### Main Findings

This study provides an up-to-date review of economic evaluations of CAR T-cell therapies for blood cancers, an area of rapid development. We found that CAR T-cell therapy was a cost-effective option, although most CAR T-cell therapies are associated with high upfront drug costs, which accounted for almost three quarters of the total cost, followed by costs related to adverse events and hospitalisation.

This review found that a combination of the relative cost of the CAR T-cell therapy versus the comparator, the magnitude of the QALY gains and the WTP thresholds determines the overall cost effectiveness of CART T-cell therapies. WTP thresholds varied widely, with some countries going well above the traditional WTP values for a QALY gain. In the USA, CAR T-cell therapy often exceeded a WTP of US$100,000 per QALY gained but was still recommended for use [32, 34, 35, 39, 45, 59, 63, 68]. In contrast, CAR T-cell therapy was not recommended in China for axicabtagene ciloleucel, tisagenlecleucel, lisocabtagene maraleucel and demonstrated 0% probability of being cost effective, at a WTP threshold of US$191,875, due to their high cost per QALY gained [16, 38]. We recognise that many studies used a WTP threshold that is not officially endorsed by HTA agencies or government. For example, the USA does not have a mandated willingness-to-pay (WTP) threshold for healthcare interventions. In the regression analysis, we investigated whether the cost effectiveness of CAR T-cell therapies were associated with any clinical and contextual factors, such as the type of CAR T-cell therapy, type of cancer, maturity of the efficacy evidence (overall survival), treatment line, population, price of CAR T-cell therapy, country and funding source. There was some evidence that maturity of the efficacy evidence (overall survival) was positively associated with the therapy’s cost effectiveness, but none of the other factors appeared to be cost-effectiveness drivers.

### Contributions

This paper complements previous published reviews on the cost effectiveness of CAR T-cell therapies. Previous reviews on ATMPs focused on methodological aspects of the economics of cell and gene therapies [17–22], highlighting the challenges around immature data [21] and drawing upon the use of methodological choices by authors using the same clinical data and the impact of this on recommendations [20].

The overall findings from our review differ with the literature in ATMP reviews. Many of the reviews of ATMPs have included gene therapy products for rare diseases, while our focus has been purely on cell therapy for blood cancers. We have incorporated published studies beyond Europe and USA, including CAR T-cell therapy approved by the Chinese NMPA.

We provide a comprehensive update to reviews by Petrou [23, 24] and Thavorn et al. [25]. Petrou provides a summary of the effectiveness, costs and the cost effectiveness for each individual CAR T-cell therapy. Our review extends Petrou’s work in several ways. Firstly, we conducted a more up-to-date review up to January 2024 given that this is an area of rapid development, and included studies not considered by Petrou’s review. Secondly, we provided a fuller description of the existing economic evaluations across disease areas and countries. We include 18 additional CAR T-cell therapy studies [15, 16, 36, 37, 41, 42, 49, 54, 58, 61–63, 65–69] and explore the relationship between treatment line and cost per QALY gained, an evolving topic in the field as CAR T-cell therapy makes its way to front-line treatment [73–76]. This offers valuable insights into how the positioning of CAR T-cell therapies in the treatment pathway influence their cost effectiveness. A more recent review by Thavorn et al. [25] included broader types of economic analyses, such as cost studies (2/47) and other non-peer reviewed reports (2/47), whereas our review is focused on published full economic evaluations (e.g. cost-utility and cost-effectiveness analyses). As a result, Thavorn’s review ended up including more studies than ours, even though our review included more recent full economic evaluations. Thavorn et al. [25] compared the use of CAR T-cell therapy in adults versus paediatric patients, reporting cost-effectiveness results are sensitive to patient population (adults versus paediatrics), type of cancer and model assumptions and identified the cost of CAR T-cell therapy as the key driver of cost effectiveness. While our findings support that the cost of CAR T-cell therapy is a key driver, we undertook a careful assessment of reported study-level characteristics and the different cost-effectiveness components. We found that the cost of CAR T-cell therapy alone does not drive cost effectiveness. Two additional key cost drivers, adverse events and hospitalisation readmissions, seem to play a key role in the cost effectiveness of delivering CAR T-cell therapy. Understanding how these costs contribute to the overall high cost of CAR T-cell therapy is vital, especially when considering the long-term financial burden of CAR T-cell therapy on healthcare systems. The choice of comparator, incremental cost and QALY, alongside the WTP threshold, are equally imperative in determining overall cost effectiveness. Furthermore, while Thavorn et al. [25] focused on how the type of CAR T-cell therapy determines their overall cost effectiveness, we conducted a broader investigation of the cost-effectiveness drivers, including type of CAR-T product, therapy price, treatment line, country, funder, population, maturity of the efficacy evidence (overall survival), and type of cancer.

### Limitations

Limitations of this review were the exclusion of grey literature and non-English reports/papers due to a lack of resources and translators. Therefore, we may have missed reports/papers, thus impacting on the generalisability of our conclusions on key cost-effectiveness drivers. Despite institutional affiliations and authorship playing a significant role in academic publishing bias, we did not blind the author or institute while reviewing the selected papers. We followed the PRISMA statement (Fig. 1) [26] and used a second reviewer to screen a sample of the records. We do recognise that this approach is somewhat less robust than a full double screening of all papers with two independent reviewers. In addition, we limited this review to the selected databases, at the risk of missing emerging studies. We acknowledge that the lack of transparency and reporting on adverse events in the included literature may have affected our conclusions. Eighteen comparisons failed to report adverse events associated with standard care [16, 29, 32, 36, 38, 41–43, 48, 49, 57, 58, 60, 64], but included the CAR T-cell-related adverse events. This may have led to underestimating costs and overestimating QALYs associated with standard care, hence impacting incremental costs and cost effectiveness of CAR T-cell therapy versus standard care. We recommend the inclusion of adverse events to capture and quantify the economic burden of treatment and additional use of healthcare resources. In future health economic analyses, resource use associated with different forms of cytokine release syndrome (CRS) and neurologic toxicity could be better reported for accuracy for costing purposes. Similarly, the costs associated with hospitalisation and bed days for treating adverse events need to be reported more comprehensively.

Moreover, we did not formally assess publication bias in published studies. While our review included studies with both positive and negative recommendations, there remains a potential favouring of studies with positive outcomes, as they are often considered more impactful and tend to attract greater interest from policy makers and healthcare providers.

### Further Research

Studies in this review were mainly conducted from a public payer perspective without accounting for the broader societal impacts of treatment on cost and the quality of life of caregivers and patients. Only five studies [36, 39, 44, 50, 65, 69] included societal costs, four studies included either caregiver or patient time, patient travel and informal care [36, 39, 44, 50, 69]. Three studies included the cost of productivity loss [36, 50, 65] in the base case analysis, while two studies measured productivity gains in the sensitivity analyses [8, 9]. Including costs from a societal perspective is likely to enhance the benefits of CAR T-cell therapy. We found more than half of the published studies use proxy utility estimates, resulting in variation in cost-effectiveness estimates. Future research should focus on generating primary utility data to enhance the precision of cost-effectiveness analyses. While we applied purchasing power parities (PPP) to account for cost differences, variations in costing methods across different countries make comparisons difficult. A standardised and transparent approach is needed to improve comparability. A specialised health economics framework for emerging cell therapies would allow a comparative analysis between the costs, benefits, and ICER of CAR T-cell therapies. Two studies [53, 54] included budget impact analyses (BIA) to estimate budgetary implications of introducing CAR T-cell therapy in the healthcare system. Given the high cost associated with CAR T-cell therapy, BIA should be applied in conjunction with cost-effectiveness studies to help inform reimbursement decisions. Finally, future studies should disentangle the cost components of the study to enable a better understanding of the different cost inputs in the economic models. For example, it is difficult to ascertain how much of the hospitalisation cost was attributable to patient recovery and how much of the total cost was attributable to adverse event ICU inpatient stay.

## Conclusion

This review of the published evidence on the cost effectiveness of CAR T-cell therapies found potential QALY gains despite high costs, with no single WTP threshold consistently applied across countries. Key drivers of cost effectiveness were the cost of CAR T-cell therapy, hospitalisation and adverse events. Furthermore, we found no statistically significant relationship between treatment line and cost per QALY gained. To confirm our findings, future research should incorporate long-term data and real-world evidence to improve the accuracy of cost-effectiveness estimates, enabling policymakers to make informed decisions regarding the reimbursement and implementation of cell therapies.

## Supplementary Information

Below is the link to the electronic supplementary material.

Supplementary file1 (PDF 1264 KB)

## Declarations

### Availability of data and material

Search strategies and data extraction templates are available in the supplementary material. This review is based upon previously conducted studies, and all data are publicly available in the referenced publications.
