# Supplementary material for: Cost-Effectiveness of Chimeric Antigen Receptor (CAR) T-Cell Therapy for Blood Cancers: An Updated Systematic Review
Source: Pharmacoecon Open. 2025 Dec 15;10(1):35–51. doi: 10.1007/s41669-025-00614-x (PMC12796026; doi:10.1007/s41669-025-00614-x)
Supplement: Supplementary file 1 — Supplementary file1 (PDF 1264 KB) [file 41669_2025_614_MOESM1_ESM.pdf]

Supplementary Material

Pharmacoeconomics - Open

Cost-effectiveness of Chimeric Antigen Receptor (CAR) T-cell Therapy for Blood Cancers: An Updated Systematic Review

Nishma Patel[1*], Suzanne Farid[2], Manuel Gomes[1]

[1]Department of Primary Care and Population Health, University College London, London, United Kingdom

[2]Department of Biochemical Engineering, University College London, United Kingdom

*Corresponding author: Nishma Patel

Department of Primary Care and Population Health

University College London

Gower St

London WC1E 6BT

nishma.patel@ucl.ac.uk

Figures

Figure S1: CAR T-cell therapy by study country (n)
Figure S2: Study conclusion by funding source (n)
Figure S3: Incremental quality adjusted life years (QALYs)
Figure S4: Incremental cost-effectiveness ratio
Figure S5: Probability cost-effective (%) by price of CAR T (US$)
Figure S6: Probability cost-effective (%) by incremental QALY
Figure S7: Probability cost-effective (%) by incremental cost (US$)
Figure S8: Incremental cost (US$) and QALYs by CAR T-cell Therapy

Tables

Table S1: MeSH and Free-text Search Terms
Table S2: Population, intervention, outcome, inclusion and exclusion criteria
Table S3: CAR T-cell therapy spending by cost component (US$)
Table S4: Payer perspective comparisons including head-to-head CAR T-cell therapy comparisons
Table S5: Multiple linear regression analysis, relationship between cost per QALY gained, line of treatment and predictor variables
Table S6: Consolidated Health Economic Evaluation Reporting Standards (CHEERS) Checklist 2022

FIGURES

Figure S1: CAR T-cell therapy by study country (n)

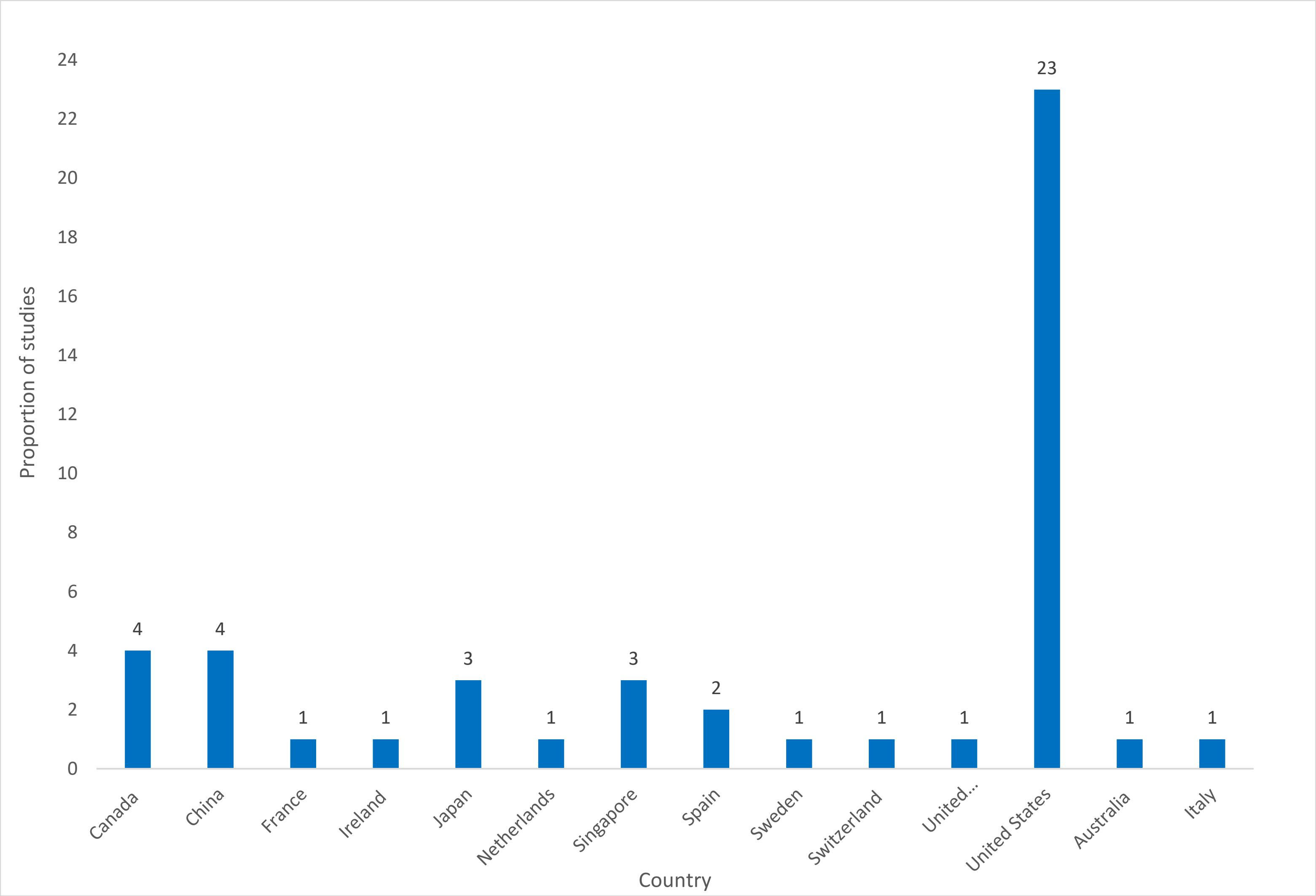

45 papers, two papers report cost-effectiveness results for two countries (26, 27), n = 47.

Figure S2: Study conclusion by funding source (n)

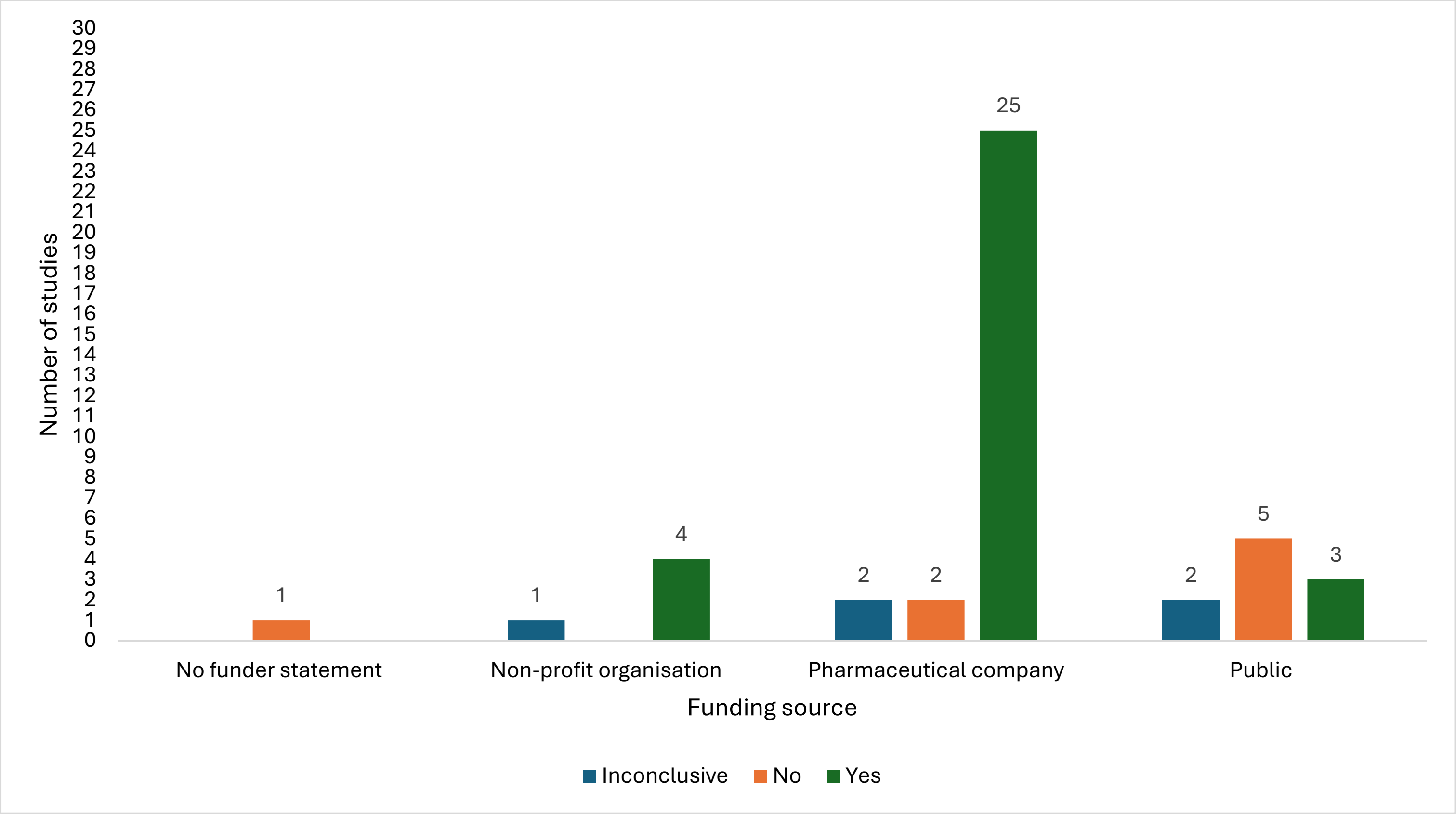

45 papers, two papers report cost-effectiveness results for two countries (26, 27) and six papers gave two perspectives.

Figure S3: Incremental quality adjusted life years (QALYs)

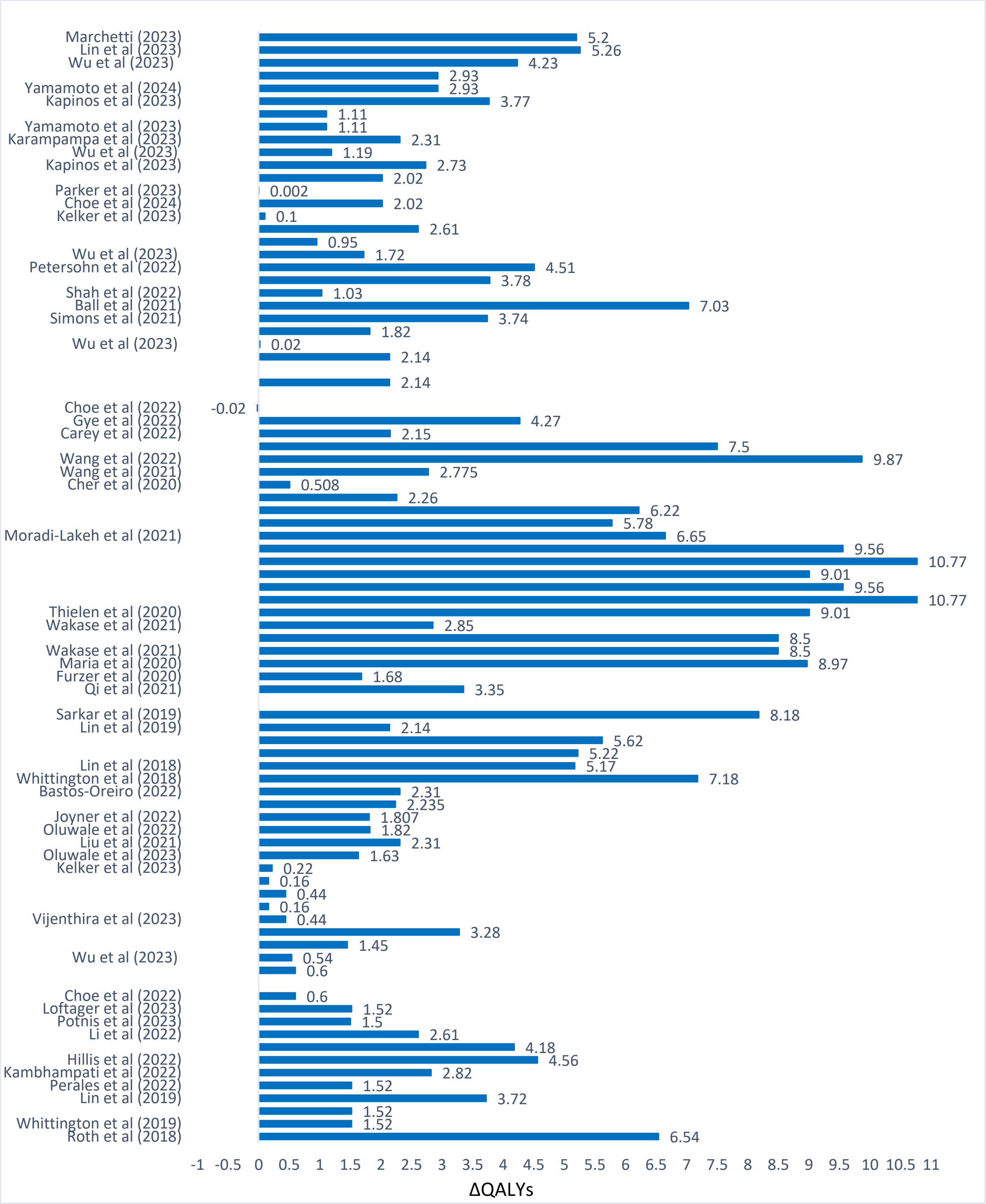

n = 92 comparisons, including societal perspective.

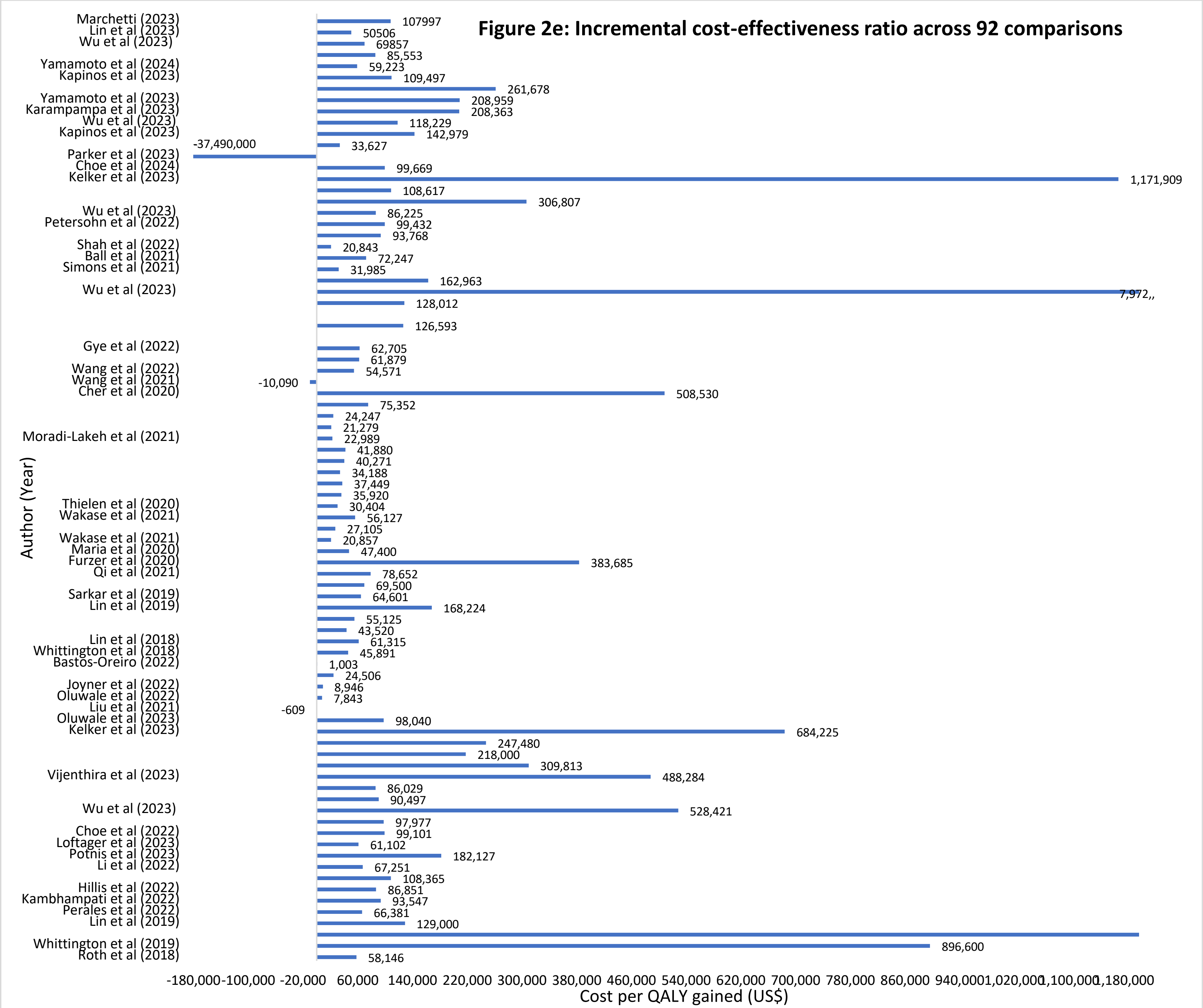

Figure 2e: Incremental cost-effectiveness ratio across 92 comparisons

Figure S5: Probability cost-effective (%) by price of CAR T (US$)

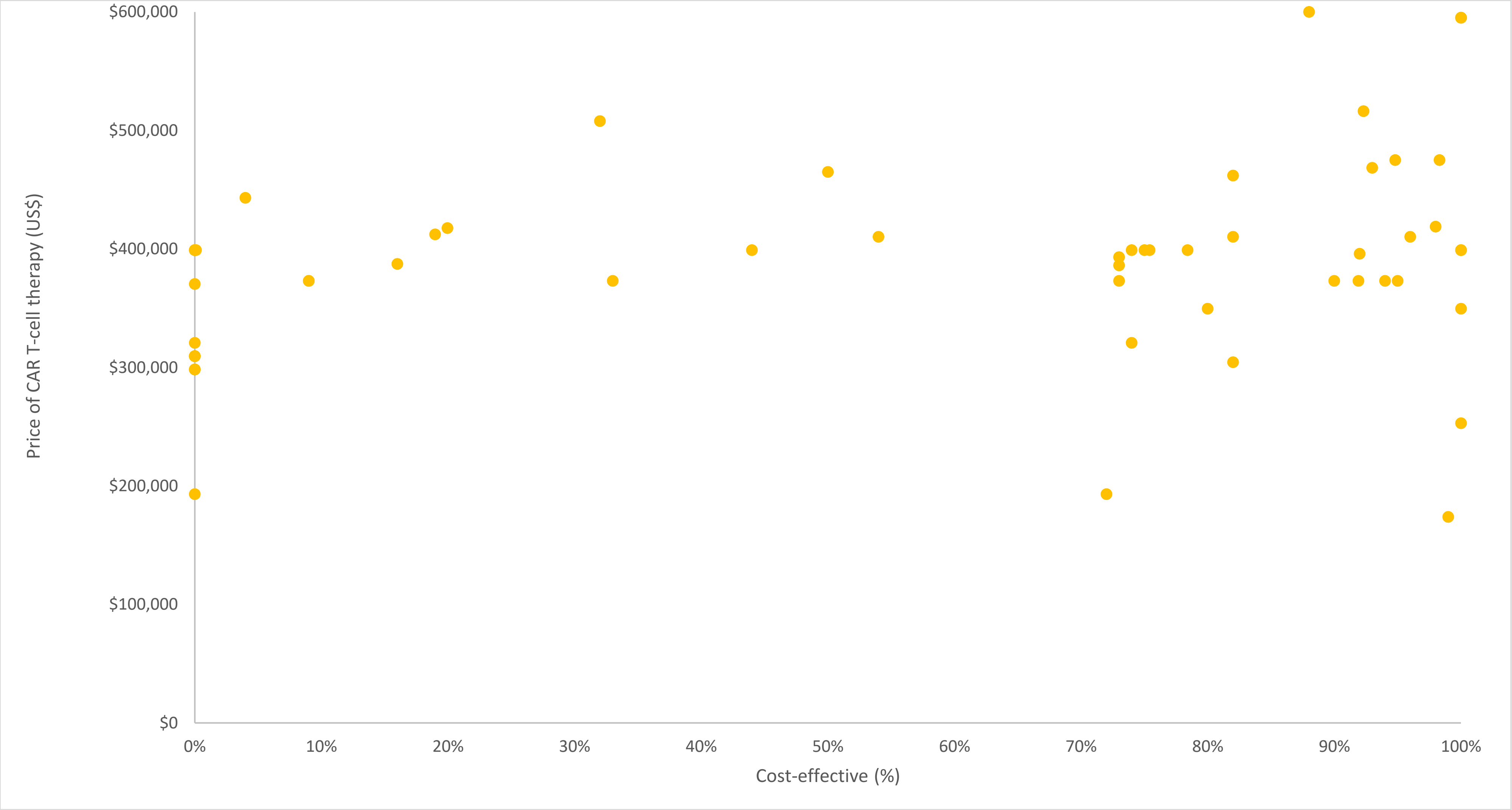

n = 63 comparisons. Where two or more perspectives are given and one is societal, we took the payer perspective. Excluded studies without reporting of cost-effective (%) or WTP (US$) but includes CAR T V CAR T comparisons.

Figure S6: Probability cost-effective (%) by incremental QALY

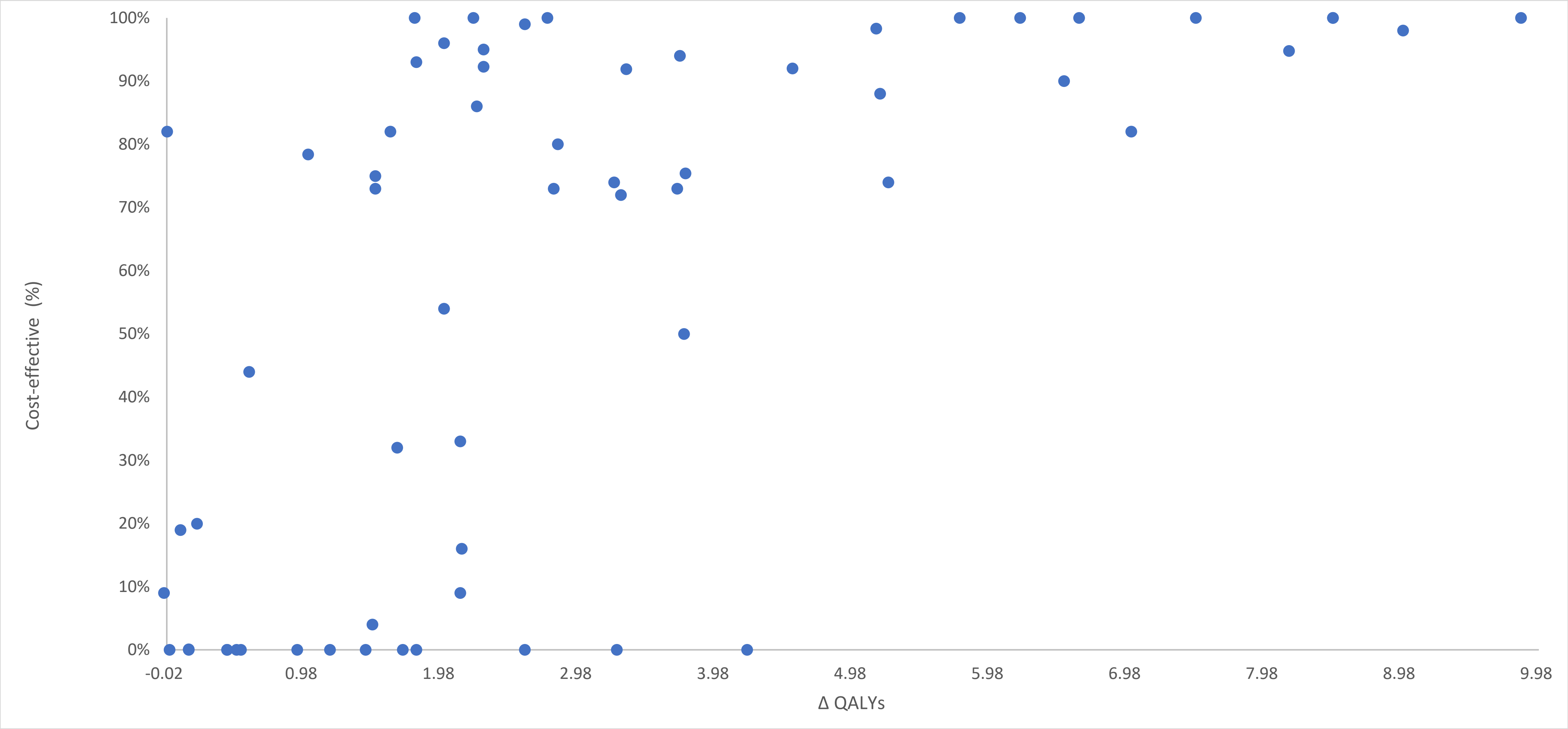

n = 63 comparisons. Where two or more perspectives are given and one is societal, we took the payer perspective. Excluded studies without reporting of cost-effective (%) or WTP (US$) but includes CAR T V CAR T comparisons.

Figure S7: Probability cost-effective (%) by incremental cost (US$)

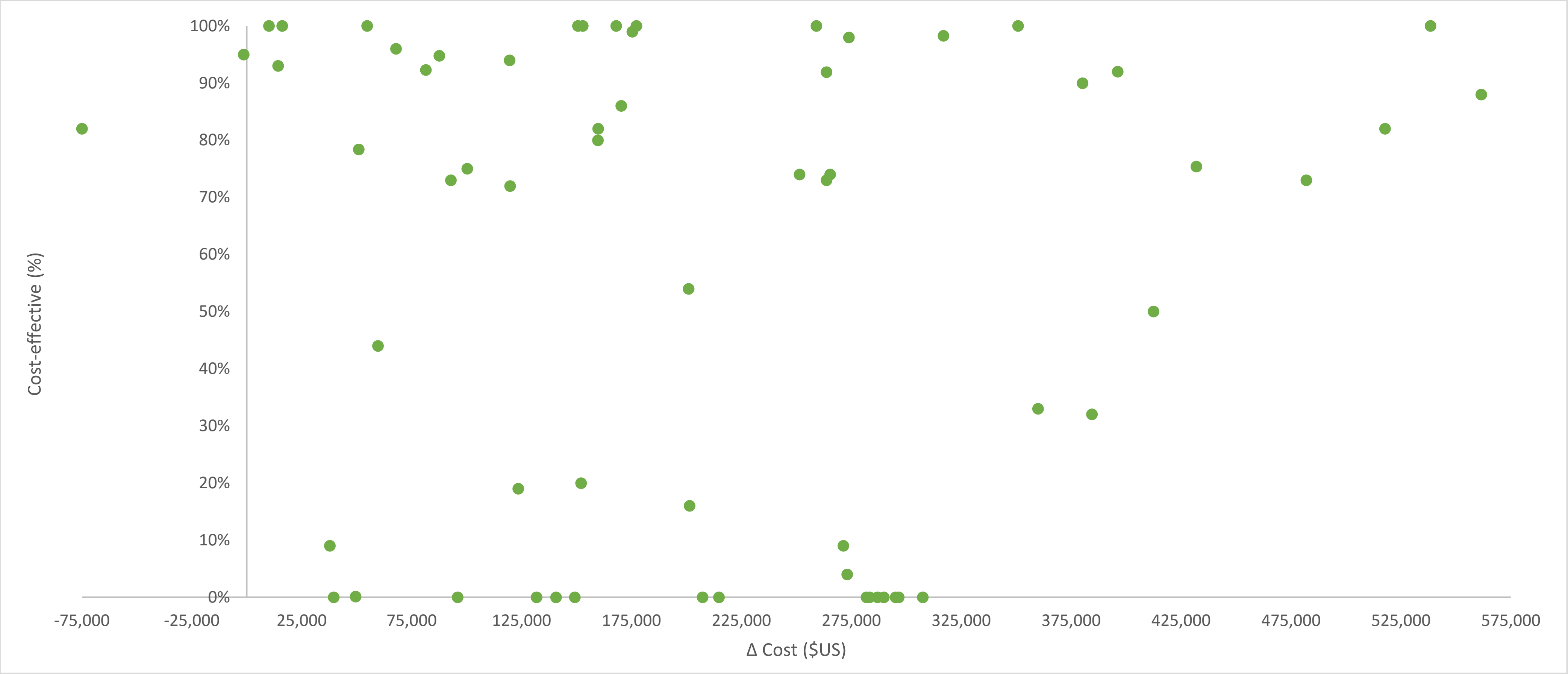

n = 63 comparisons. Where two or more perspectives are given and one is societal, we took the payer perspective. Excluded studies without reporting of cost-effective (%) or WTP (US$) but includes CAR T V CAR T comparisons.

Figure S8: Incremental cost (US$) and QALYs by CAR T-cell Therapy

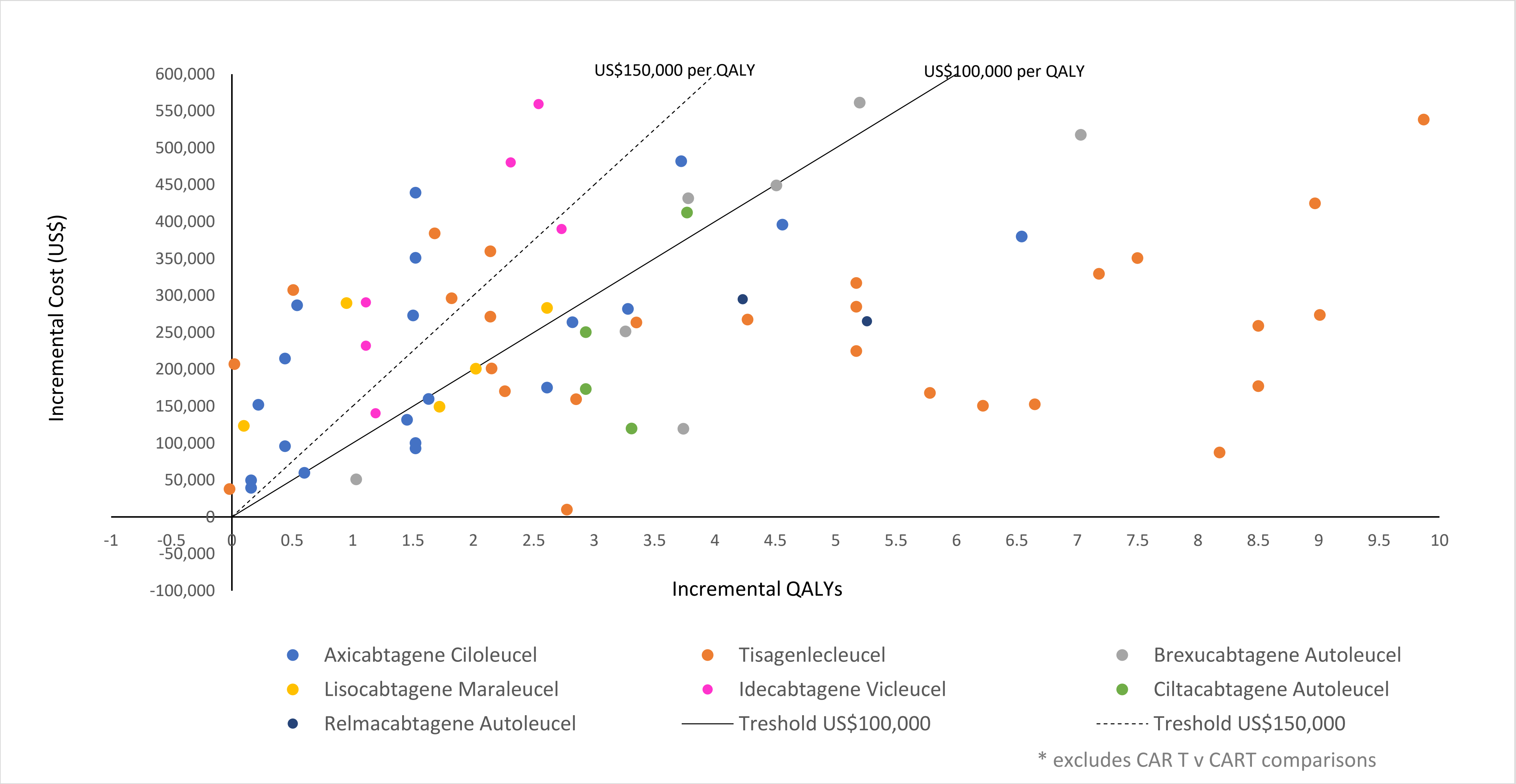

n = 73 comparisons. Where two perspectives are given and one is societal, we took the payer perspective. Excludes CAR T v CAR T comparisons.

TABLES

Table S1: MeSH and Free-text Search Terms

Healthcare Database

MeSH (Medical Subject Headings) is the NLM controlled vocabulary thesaurus used for indexing articles for PubMed, 1st January 2017 to 31st January 2024 (MeSH database)

Search number	Query	Filters	Search Details	Results
14	("Cell- and Tissue-Based Therapy"(Mesh) OR "Precision Medicine"(Mesh) OR "Regenerative Medicine"(Mesh) OR "Receptors, Chimeric Antigen"(Mesh) OR "Genetic Therapy"(Mesh) OR "Antigens, CD19"(Mesh) AND (2017/1/1:2024/1/31(pdat))) AND (("Reimbursement Mechanisms"(Mesh)) AND "Costs and Cost Analysis"(Mesh) AND (2017/1/1:2024/1/31(pdat)))	from 2017/1/1 - 2024/1/31	(("cell and tissue based therapy"(MeSH Terms) OR "Precision Medicine"(MeSH Terms) OR "Regenerative Medicine"(MeSH Terms) OR "receptors, chimeric antigen"(MeSH Terms) OR "Genetic Therapy"(MeSH Terms) OR "antigens, cd19"(MeSH Terms)) AND 2017/01/01:2024/01/31(Date - Publication] AND ("Reimbursement Mechanisms"(MeSH Terms) AND "Costs and Cost Analysis"(MeSH Terms) AND 2017/01/01:2024/01/31(Date – Publication))) AND (2017/1/1:2024/1/31(pdat))	7
13	("Cell- and Tissue-Based Therapy"(Mesh) OR "Precision Medicine"(Mesh) OR "Regenerative Medicine"(Mesh) OR "Receptors, Chimeric Antigen"(Mesh) OR "Genetic Therapy"(Mesh) OR "Antigens, CD19"(Mesh) AND (2017/1/1:2024/1/31(pdat))) AND ("Cost-Benefit Analysis"(Mesh) OR "Cost-Effectiveness Analysis"(Mesh) OR "Technology Assessment, Biomedical"(Mesh) AND (2017/1/1:2024/1/31(pdat)))	from 2017/1/1 - 2024/1/31	(("cell and tissue based therapy"(MeSH Terms) OR "Precision Medicine"(MeSH Terms) OR "Regenerative Medicine"(MeSH Terms) OR "receptors, chimeric antigen"(MeSH Terms) OR "Genetic Therapy"(MeSH Terms) OR "antigens, cd19"(MeSH Terms)) AND 2017/01/01:2024/01/31(Date – Publication) AND (("Cost-Benefit Analysis"(MeSH Terms) OR "Cost-Effectiveness Analysis"(MeSH Terms) OR "technology assessment, biomedical"(MeSH Terms)) AND 2017/01/01:2024/01/31(Date – Publication))) AND (2017/1/1:2024/1/31(pdat))	476
12	Ciltacabtagene autoleucel(MeSH Terms)	from 2017/1/1 - 2024/1/31	(Ciltacabtagene autoleucel(MeSH Terms)) AND (2017/1/1:2024/1/31(pdat))	0

11	Lisocabtagene maraleucel(MeSH Terms)	from 2017/1/1 - 2024/1/31	(Lisocabtagene maraleucel(MeSH Terms)) AND (2017/1/1:2024/1/31(pdat))	0
10	((((("tisagenlecleucel" (Supplementary Concept) AND (2017/1/1:2024/1/31(pdat))) OR ("axicabtagene ciloleucel" (Supplementary Concept) AND (2017/1/1:2024/1/31(pdat)))) OR ("brexucabtagene autoleucel" (Supplementary Concept) AND (2017/1/1:2024/1/31(pdat)))) OR ("idecabtagene vicleucel" (Supplementary Concept) AND (2017/1/1:2024/1/31(pdat)))) OR ("relmacabtagene autoleucel" (Supplementary Concept) AND (2017/1/1:2024/1/31(pdat)))) AND (("Reimbursement Mechanisms"(Mesh)) AND "Costs and Cost Analysis"(Mesh) AND (2017/1/1:2024/1/31(pdat)))	from 2017/1/1 - 2024/1/31	((("tisagenlecleucel"(Supplementary Concept) AND 2017/01/01:2024/01/31(Date - Publication)]) OR ("axicabtagene ciloleucel"(Supplementary Concept) AND 2017/01/01:2024/01/31(Date – Publication)) OR ("brexucabtagene autoleucel(Supplementary Concept) AND 2017/01/01:2024/01/31(Date – Publication)) OR ("idecabtagene vicleucel"(Supplementary Concept) AND 2017/01/01:2024/01/31(Date – Publication)) OR ("relmacabtagene autoleucel"(Supplementary Concept) AND 2017/01/01:2024/01/31(Date – Publication))) AND ("Reimbursement Mechanisms"(MeSH Terms) AND "Costs and Cost Analysis"(MeSH Terms) AND 2017/01/01:2024/01/31(Date – Publication))) AND (2017/1/1:2024/1/31(pdat))	0
]	((((("tisagenlecleucel" (Supplementary Concept) AND (2017/1/1:2024/1/31(pdat))) OR ("axicabtagene ciloleucel" (Supplementary Concept) AND (2017/1/1:2024/1/31(pdat)))) OR ("brexucabtagene autoleucel" (Supplementary Concept) AND (2017/1/1:2024/1/31(pdat)))) OR ("idecabtagene vicleucel" (Supplementary Concept) AND (2017/1/1:2024/1/31(pdat)))) OR ("relmacabtagene autoleucel" (Supplementary Concept) AND (2017/1/1:2024/1/31(pdat)))) AND ("Cost-Benefit Analysis"(Mesh) OR "Cost-Effectiveness Analysis"(Mesh) OR "Technology Assessment, Biomedical"(Mesh) AND (2017/1/1:2024/1/31(pdat)))	from 2017/1/1 - 2024/1/31	((("tisagenlecleucel"(Supplementary Concept) AND 2017/01/01:2024/01/31(Date – Publication)) OR ("axicabtagene ciloleucel"(Supplementary Concept) AND 2017/01/01:2024/01/31(Date – Publication)) OR ("brexucabtagene autoleucel"(Supplementary Concept) AND 2017/01/01:2024/01/31(Date – Publication)) OR ("idecabtagene vicleucel"(Supplementary Concept) AND 2017/01/01:2024/01/31(Date – Publication)) OR ("relmacabtagene autoleucel"(Supplementary Concept) AND 2017/01/01:2024/01/31(Date – Publication))) AND (("Cost-Benefit Analysis"(MeSH Terms) OR "Cost-Effectiveness Analysis"(MeSH Terms) OR "technology assessment,	48

			biomedical"(MeSH Terms)) AND 2017/01/01:2024/01/31(Date - Publication])) AND (2017/1/1:2024/1/31(pdat))	
8	("Reimbursement Mechanisms"(Mesh)) AND "Costs and Cost Analysis"(Mesh)	from 2017/1/1 - 2024/1/31	("Reimbursement Mechanisms"(MeSH Terms) AND "Costs and Cost Analysis"(MeSH Terms)) AND (2017/1/1:2024/1/31(pdat))	1,189
7	"relmacabtagene autoleucel" (Supplementary Concept)	from 2017/1/1 - 2024/1/31	("relmacabtagene autoleucel"(Supplementary Concept)) AND (2017/1/1:2024/1/31(pdat))	5
6	"idecabtagene vicleucel" (Supplementary Concept)	from 2017/1/1 - 2024/1/31	("idecabtagene vicleucel"(Supplementary Concept)) AND (2017/1/1:2024/1/31(pdat))	53
5	"brexucabtagene autoleucel" (Supplementary Concept)	from 2017/1/1 - 2024/1/31	("brexucabtagene autoleucel"(Supplementary Concept)) AND (2017/1/1:2024/1/31(pdat))	26
4	"axicabtagene ciloleucel" (Supplementary Concept)	from 2017/1/1 - 2024/1/31	("axicabtagene ciloleucel"(Supplementary Concept)) AND (2017/1/1:2024/1/31(pdat))	192
3	"tisagenlecleucel" (Supplementary Concept)	from 2017/1/1 - 2024/1/31	("tisagenlecleucel"(Supplementary Concept)) AND (2017/1/1:2024/1/31(pdat))	236
2	"Cost-Benefit Analysis"(Mesh) OR "Cost-Effectiveness Analysis"(Mesh) OR "Technology Assessment, Biomedical"(Mesh)	from 2017/1/1 - 2024/1/31	("Cost-Benefit Analysis"(MeSH Terms) OR "Cost-Effectiveness Analysis"(MeSH Terms) OR "technology assessment, biomedical"(MeSH Terms)) AND (2017/1/1:2024/1/31(pdat))	25,879
1	"Cell- and Tissue-Based Therapy"(Mesh) OR "Precision Medicine"(Mesh) OR "Regenerative Medicine"(Mesh) OR "Receptors, Chimeric Antigen"(Mesh) OR "Genetic Therapy"(Mesh) OR "Antigens, CD19"(Mesh)	from 2017/1/1 - 2024/1/31	("cell and tissue based therapy"(MeSH Terms) OR "Precision Medicine"(MeSH Terms) OR "Regenerative Medicine"(MeSH Terms) OR "receptors, chimeric antigen"(MeSH Terms) OR "Genetic Therapy"(MeSH Terms) OR "antigens, cd19"(MeSH Terms)) AND (2017/1/1:2024/1/31(pdat))	106,204
**Free-text search in PubMed**				
**Search number**	**Query**	**Filters**	**Search Details**	**Results**

5	(Tisagenlecleucel OR Kymriah OR Axicabtagene ciloleucel OR Yescarta OR Brexucabtagene autoleucel OR Tecartus OR Lisocabtagene maraleucel OR Breyanzi OR Idecabtagene vicleucel OR Abecma OR Ciltacabtagene autoleucel OR Carvykti OR Relmacabtagene autoleucel OR Carteyva AND (2017/1/1:2024/1/31(pdat))) AND ((Economic Evaluation OR Decision Modelling OR Budget impact OR Cost Utility Analysis OR Cost Effectiveness Analysis OR Cost Benefit Analysis OR Health Technology Assessment OR Value for money AND (2017/1/1:2024/1/31(pdat))) OR (Reimburse* AND Cost Analysis AND (2017/1/1:2024/1/31(pdat))) AND (2017/1/1:2024/1/31(pdat)))	from 2017/1/1 - 2024/1/31	(("tisagenlecleucel"(Supplementary Concept) OR "tisagenlecleucel"(All Fields) OR ("tisagenlecleucel"(Supplementary Concept) OR "tisagenlecleucel"(All Fields) OR "kymriah"(All Fields)) OR ("axicabtagene ciloleucel"(Supplementary Concept) OR "axicabtagene ciloleucel"(All Fields)) OR ("axicabtagene ciloleucel"(Supplementary Concept) OR "axicabtagene ciloleucel"(All Fields) OR "yescarta"(All Fields)) OR ("brexucabtagene autoleucel"(Supplementary Concept) OR "brexucabtagene autoleucel"(All Fields)) OR ("brexucabtagene autoleucel"(Supplementary Concept) OR "brexucabtagene autoleucel"(All Fields) OR "tecartus"(All Fields)) OR ("Lisocabtagene"(All Fields) AND "maraleucel"(All Fields)) OR ("breyanzi"(All Fields) OR "lisocabtagene maraleucel"(All Fields)) OR ("idecabtagene vicleucel"(Supplementary Concept) OR "idecabtagene vicleucel"(All Fields)) OR ("abecma"(All Fields) OR "idecabtagene vicleucel"(Supplementary Concept) OR "idecabtagene vicleucel"(All Fields)) OR ("Ciltacabtagene"(All Fields) AND "autoleucel"(All Fields)) OR "Carvykti"(All Fields) OR ("relmacabtagene autoleucel"(Supplementary Concept) OR "relmacabtagene autoleucel"(All Fields)) OR "Carteyva"(All Fields)) AND 2017/01/01:2024/01/31(Date - Publication) AND (((("cost benefit analysis"(MeSH Terms) OR ("cost benefit"(All Fields) AND "analysis"(All Fields)) OR "cost benefit analysis"(All Fields) OR ("economic"(All Fields) AND "evaluation"(All Fields)) OR "economic evaluation"(All Fields) OR ("decision modelling"(All Fields) OR "decision support techniques"(MeSH Terms) OR ("decision"(All Fields) AND "support"(All Fields) AND "techniques"(All Fields)) OR "decision support	**109**

			techniques"(All Fields) OR ("decision"(All Fields) AND "modeling"(All Fields)) OR "decision modeling"(All Fields)) OR (("budget s"(All Fields) OR "budgeted"(All Fields) OR "budgets"(MeSH Terms) OR "budgets"(All Fields) OR "budget"(All Fields) OR "budgeting"(All Fields)) AND ("impact"(All Fields) OR "impactful"(All Fields) OR "impacting"(All Fields) OR "impacts"(All Fields) OR "tooth, impacted"(MeSH Terms) OR ("tooth"(All Fields) AND "impacted"(All Fields)) OR "impacted tooth"(All Fields) OR "impacted"(All Fields))) OR ("cost benefit analysis"(MeSH Terms) OR ("cost benefit"(All Fields) AND "analysis"(All Fields)) OR "cost benefit analysis"(All Fields) OR ("cost"(All Fields) AND "utility"(All Fields) AND "analysis"(All Fields)) OR "cost utility analysis"(All Fields)) OR ("cost effectiveness analysis"(MeSH Terms) OR ("cost effectiveness"(All Fields) AND "analysis"(All Fields)) OR "cost effectiveness analysis"(All Fields) OR ("cost"(All Fields) AND "effectiveness"(All Fields) AND "analysis"(All Fields)) OR "cost effectiveness analysis"(All Fields)) OR ("cost benefit analysis"(MeSH Terms) OR ("cost benefit"(All Fields) AND "analysis"(All Fields)) OR "cost benefit analysis"(All Fields) OR ("cost"(All Fields) AND "benefit"(All Fields) AND "analysis"(All Fields)) OR "cost benefit analysis"(All Fields)) OR ("technology assessment, biomedical"(MeSH Terms) OR ("technology"(All Fields) AND "assessment"(All Fields) AND "biomedical"(All Fields)) OR "biomedical technology assessment"(All Fields) OR ("health"(All Fields) AND "technology"(All Fields) AND "assessment"(All Fields)) OR "health technology assessment"(All Fields)) OR (("value"(All Fields) OR "values"(All Fields)) AND ("money"(All Fields) OR "money s"(All Fields) OR "moneys"(All	

			Fields)))) AND 2017/01/01:2024/01/31(Date - Publication)) OR ("reimburse*"(All Fields) AND ("costs and cost analysis"(MeSH Terms) OR ("costs"(All Fields) AND "cost"(All Fields) AND "analysis"(All Fields)) OR "costs and cost analysis"(All Fields) OR ("cost"(All Fields) AND "analysis"(All Fields)) OR "cost analysis"(All Fields)) AND 2017/01/01:2024/01/31(Date - Publication))) AND 2017/01/01:2024/01/31(Date – Publication))) AND (2017/1/1:2024/1/31(pdat))	
4	(Economic Evaluation OR Decision Modelling OR Budget impact OR Cost Utility Analysis OR Cost Effectiveness Analysis OR Cost Benefit Analysis OR Health Technology Assessment OR Value for money AND (2017/1/1:2024/1/31(pdat))) OR (Reimburse* AND Cost Analysis AND (2017/1/1:2024/1/31(pdat)))	from 2017/1/1 - 2024/1/31	((("cost benefit analysis"(MeSH Terms) OR ("cost benefit"(All Fields) AND "analysis"(All Fields)) OR "cost benefit analysis"(All Fields) OR ("economic"(All Fields) AND "evaluation"(All Fields)) OR "economic evaluation"(All Fields) OR ("decision modelling"(All Fields) OR "decision support techniques"(MeSH Terms) OR ("decision"(All Fields) AND "support"(All Fields) AND "techniques"(All Fields)) OR "decision support techniques"(All Fields) OR ("decision"(All Fields) AND "modeling"(All Fields)) OR "decision modeling"(All Fields)) OR (("budget s"(All Fields) OR "budgeted"(All Fields) OR "budgets"(MeSH Terms) OR "budgets"(All Fields) OR "budget"(All Fields) OR "budgeting"(All Fields)) AND ("impact"(All Fields) OR "impactful"(All Fields) OR "impacting"(All Fields) OR "impacts"(All Fields) OR "tooth, impacted"(MeSH Terms) OR ("tooth"(All Fields) AND "impacted"(All Fields)) OR "impacted tooth"(All Fields) OR "impacted"(All Fields))) OR ("cost benefit analysis"(MeSH Terms) OR ("cost benefit"(All Fields) AND "analysis"(All Fields)) OR "cost benefit analysis"(All Fields) OR ("cost"(All Fields) AND "utility"(All Fields) AND "analysis"(All Fields)) OR "cost utility analysis"(All Fields)) OR ("cost effectiveness	146,029

			analysis"(MeSH Terms) OR ("cost effectiveness"(All Fields) AND "analysis"(All Fields)) OR "cost effectiveness analysis"(All Fields) OR ("cost"(All Fields) AND "effectiveness"(All Fields) AND "analysis"(All Fields)) OR "cost effectiveness analysis"(All Fields)) OR ("cost benefit analysis"(MeSH Terms) OR ("cost benefit"(All Fields) AND "analysis"(All Fields)) OR "cost benefit analysis"(All Fields) OR ("cost"(All Fields) AND "benefit"(All Fields) AND "analysis"(All Fields)) OR "cost benefit analysis"(All Fields)) OR ("technology assessment, biomedical"(MeSH Terms) OR ("technology"(All Fields) AND "assessment"(All Fields) AND "biomedical"(All Fields)) OR "biomedical technology assessment"(All Fields) OR ("health"(All Fields) AND "technology"(All Fields) AND "assessment"(All Fields)) OR "health technology assessment"(All Fields)) OR (("value"(All Fields) OR "values"(All Fields)) AND ("money"(All Fields) OR "money s"(All Fields) OR "moneys"(All Fields)))) AND 2017/01/01:2024/01/31(Date - Publication)) OR ("reimburse*"(All Fields) AND ("costs and cost analysis"(MeSH Terms) OR ("costs"(All Fields) AND "cost"(All Fields) AND "analysis"(All Fields)) OR "costs and cost analysis"(All Fields) OR ("cost"(All Fields) AND "analysis"(All Fields)) OR "cost analysis"(All Fields)) AND 2017/01/01:2024/01/31(Date - Publication))) AND (2017/1/1:2024/1/31(pdat))	
3	Reimburse* AND Cost Analysis	from 2017/1/1 - 2024/1/31	("reimburse*"(All Fields) AND ("costs and cost analysis"(MeSH Terms) OR ("costs"(All Fields) AND "cost"(All Fields) AND "analysis"(All Fields)) OR "costs and cost analysis"(All Fields) OR ("cost"(All Fields) AND "analysis"(All Fields)) OR "cost analysis"(All Fields))) AND (2017/1/1:2024/1/31(pdat))	4,016

2	Economic Evaluation OR Decision Modelling OR Budget impact OR Cost Utility Analysis OR Cost Effectiveness Analysis OR Cost Benefit Analysis OR Health Technology Assessment OR Value for money	from 2017/1/1 - 2024/1/31	("cost benefit analysis"(MeSH Terms) OR ("cost benefit"(All Fields) AND "analysis"(All Fields)) OR "cost benefit analysis"(All Fields) OR ("economic"(All Fields) AND "evaluation"(All Fields)) OR "economic evaluation"(All Fields) OR ("decision modelling"(All Fields) OR "decision support techniques"(MeSH Terms) OR ("decision"(All Fields) AND "support"(All Fields) AND "techniques"(All Fields)) OR "decision support techniques"(All Fields) OR ("decision"(All Fields) AND "modeling"(All Fields)) OR "decision modeling"(All Fields)) OR (("budget s"(All Fields) OR "budgeted"(All Fields) OR "budgets"(MeSH Terms) OR "budgets"(All Fields) OR "budget"(All Fields) OR "budgeting"(All Fields)) AND ("impact"(All Fields) OR "impactful"(All Fields) OR "impacting"(All Fields) OR "impacts"(All Fields) OR "tooth, impacted"(MeSH Terms) OR ("tooth"(All Fields) AND "impacted"(All Fields)) OR "impacted tooth"(All Fields) OR "impacted"(All Fields))) OR ("cost benefit analysis"(MeSH Terms) OR ("cost benefit"(All Fields) AND "analysis"(All Fields)) OR "cost benefit analysis"(All Fields) OR ("cost"(All Fields) AND "utility"(All Fields) AND "analysis"(All Fields)) OR "cost utility analysis"(All Fields)) OR ("cost effectiveness analysis"(MeSH Terms) OR ("cost effectiveness"(All Fields) AND "analysis"(All Fields)) OR "cost effectiveness analysis"(All Fields) OR ("cost"(All Fields) AND "effectiveness"(All Fields) AND "analysis"(All Fields)) OR "cost effectiveness analysis"(All Fields)) OR ("cost benefit analysis"(MeSH Terms) OR ("cost benefit"(All Fields) AND "analysis"(All Fields)) OR "cost benefit analysis"(All Fields) OR ("cost"(All Fields) AND "benefit"(All Fields) AND "analysis"(All Fields)) OR "cost benefit analysis"(All Fields)) OR ("technology assessment, biomedical"(MeSH	143,872

			Terms) OR ("technology"(All Fields) AND "assessment"(All Fields) AND "biomedical"(All Fields)) OR "biomedical technology assessment"(All Fields) OR ("health"(All Fields) AND "technology"(All Fields) AND "assessment"(All Fields)) OR "health technology assessment"(All Fields)) OR (("value"(All Fields) OR "values"(All Fields)) AND ("money"(All Fields) OR "money s"(All Fields) OR "moneys"(All Fields)))) AND (2017/1/1:2024/1/31(pdat))	
1	Tisagenlecleucel OR Kymriah OR Axicabtagene ciloleucel OR Yescarta OR Brexucabtagene autoleucel OR Tecartus OR Lisocabtagene maraleucel OR Breyanzi OR Idecabtagene vicleucel OR Abecma OR Ciltacabtagene autoleucel OR Carvykti OR Relmacabtagene autoleucel OR Carteyva	from 2017/1/1 - 2024/1/31	("tisagenlecleucel"(Supplementary Concept) OR "tisagenlecleucel"(All Fields) OR ("tisagenlecleucel"(Supplementary Concept) OR "tisagenlecleucel"(All Fields) OR "kymriah"(All Fields)) OR ("axicabtagene ciloleucel"(Supplementary Concept) OR "axicabtagene ciloleucel"(All Fields)) OR ("axicabtagene ciloleucel"(Supplementary Concept) OR "axicabtagene ciloleucel"(All Fields) OR "yescarta"(All Fields)) OR ("brexucabtagene autoleucel"(Supplementary Concept) OR "brexucabtagene autoleucel"(All Fields)) OR ("brexucabtagene autoleucel"(Supplementary Concept) OR "brexucabtagene autoleucel"(All Fields) OR "tecartus"(All Fields)) OR ("Lisocabtagene"(All Fields) AND "maraleucel"(All Fields)) OR ("breyanzi"(All Fields) OR "lisocabtagene maraleucel"(All Fields)) OR ("idecabtagene vicleucel"(Supplementary Concept) OR "idecabtagene vicleucel"(All Fields)) OR ("abecma"(All Fields) OR "idecabtagene vicleucel"(Supplementary Concept) OR "idecabtagene vicleucel"(All Fields)) OR ("Ciltacabtagene"(All Fields) AND "autoleucel"(All Fields)) OR "Carvykti"(All Fields) OR ("relmacabtagene autoleucel"(Supplementary Concept) OR "relmacabtagene	1,086

			autoleucel"(All Fields)) OR "Carteyva"(All Fields)) AND (2017/1/1:2024/1/31(pdat))	
**Embase, (Ovid)**				
**Search number**	**Query**	**Filters**	**Search Details**	**Results**
5	Search 1 AND Search 4	2017 - 2024		**142**
4	Search 2 OR Search 3	2017 - 2024		59,906
3	Reimburse* AND Cost Analysis	2017 - 2024		765
2	Economic Evaluation OR Decision Modelling OR Budget impact OR Cost Utility Analysis OR Cost Effectiveness Analysis OR Cost Benefit Analysis OR Health Technology Assessment OR Value for money	2017 - 2024		59,207
1	Tisagenlecleucel OR Kymriah OR Axicabtagene ciloleucel OR Yescarta OR Brexucabtagene autoleucel OR Tecartus OR Lisocabtagene maraleucel OR Breyanzi OR Idecabtagene vicleucel OR Abecma OR Ciltacabtagene autoleucel OR Carvykti OR Relmacabtagene autoleucel OR Carteyva	2017 - 2024		3,351
Multidisciplinary Database				
**Scopus**				
**Search number**	**Query**	**Filters**	**Search Details**	**Results**
5	Search 1 AND Search 4	2017 - 2024	(TITLE-ABS-KEY ({Axicabtagene ciloleucel} OR {Yescarta} OR {Brexucabtagene autoleucel} OR {Tecartus} OR {Lisocabtagene maraleucel} OR {Breyanzi} OR {Idecabtagene vicleucel} OR {Abecma} OR {Ciltacabtagene autoleucel} OR {Carvykti})) AND ((TITLE-ABS-KEY ({Economic Evaluation} OR {Decision Modelling} OR {Budget impact} OR {Cost Utility Analysis} OR {Cost Effectiveness Analysis} OR {Cost	**181**

			Benefit Analysis} OR {Health Technology Assessment} OR {Value for money})) OR (TITLE-ABS-KEY ("Reimburse*" AND {Cost Analysis}))) AND (LIMIT-TO (PUBYEAR , 2017) OR LIMIT-TO (PUBYEAR , 2018) OR LIMIT-TO (PUBYEAR , 2019) OR LIMIT-TO (PUBYEAR , 2020) OR LIMIT-TO (PUBYEAR , 2021) OR LIMIT-TO (PUBYEAR , 2022) OR LIMIT-TO (PUBYEAR , 2023) OR LIMIT-TO (PUBYEAR , 2024))	
4	Search 2 OR Search 3	2017 - 2024	(TITLE-ABS-KEY ({Economic Evaluation} OR {Decision Modelling} OR {Budget impact} OR {Cost Utility Analysis} OR {Cost Effectiveness Analysis} OR {Cost Benefit Analysis} OR {Health Technology Assessment} OR {Value for money})) OR (TITLE-ABS-KEY ("Reimburse*" AND {Cost Analysis})) AND (LIMIT-TO (PUBYEAR , 2017) OR LIMIT-TO (PUBYEAR , 2018) OR LIMIT-TO (PUBYEAR , 2019) OR LIMIT-TO (PUBYEAR , 2020) OR LIMIT-TO (PUBYEAR , 2021) OR LIMIT-TO (PUBYEAR , 2022) OR LIMIT-TO (PUBYEAR , 2023) OR LIMIT-TO (PUBYEAR , 2024))	125,868
3	Reimburse* AND Cost Analysis	2017 - 2024	TITLE-ABS-KEY ("Reimburse*" AND {Cost Analysis}) AND (LIMIT-TO (PUBYEAR , 2017) OR LIMIT-TO (PUBYEAR , 2018) OR LIMIT-TO (PUBYEAR , 2019) OR LIMIT-TO (PUBYEAR , 2020) OR LIMIT-TO (PUBYEAR , 2021) OR LIMIT-TO (PUBYEAR , 2022) OR LIMIT-TO (PUBYEAR , 2023) OR LIMIT-TO (PUBYEAR , 2024))	757
2	Economic Evaluation OR Decision Modelling OR Budget impact OR Cost Utility Analysis OR Cost Effectiveness Analysis OR Cost Benefit Analysis OR Health Technology Assessment OR Value for money	2017 - 2024	TITLE-ABS-KEY ({Economic Evaluation} OR {Decision Modelling} OR {Budget impact} OR {Cost Utility Analysis} OR {Cost Effectiveness Analysis} OR {Cost Benefit Analysis} OR {Health Technology Assessment} OR {Value for money}) AND (LIMIT-TO (PUBYEAR , 2017) OR LIMIT-TO (PUBYEAR , 2018) OR LIMIT-TO (PUBYEAR , 2019) OR LIMIT-TO (PUBYEAR , 2020) OR LIMIT-TO (PUBYEAR , 2021) OR	125,469

			LIMIT-TO (PUBYEAR , 2022) OR LIMIT-TO (PUBYEAR , 2023) OR LIMIT-TO (PUBYEAR , 2024))	
1	Tisagenlecleucel OR Kymriah OR Axicabtagene ciloleucel OR Yescarta OR Brexucabtagene autoleucel OR Tecartus OR Lisocabtagene maraleucel OR Breyanzi OR Idecabtagene vicleucel OR Abecma OR Ciltacabtagene autoleucel OR Carvykti OR Relmacabtagene autoleucel OR Carteyva	2017 - 2024	TITLE-ABS-KEY ({Tisagenlecleucel} OR {Kymriah} OR {Axicabtagene ciloleucel} OR {Yescarta} OR {Brexucabtagene autoleucel} OR {Tecartus} OR {Lisocabtagene maraleucel} OR {Breyanzi} OR {Idecabtagene vicleucel} OR {Abecma} OR {Ciltacabtagene autoleucel} OR {Carvykti}) AND (LIMIT-TO (PUBYEAR , 2017) OR LIMIT-TO (PUBYEAR , 2018) OR LIMIT-TO (PUBYEAR , 2019) OR LIMIT-TO (PUBYEAR , 2020) OR LIMIT-TO (PUBYEAR , 2021) OR LIMIT-TO (PUBYEAR , 2022) OR LIMIT-TO (PUBYEAR , 2023) OR LIMIT-TO (PUBYEAR , 2024))	2,933

Table S2: Population, intervention, outcome, inclusion and exclusion criteria

	Include	Exclude
**Population**	• Users treated with chimeric antigen receptor (CAR)T-cell therapy	• Not applicable
**Intervention & Comparator**	• Not applicable	• Not applicable
**Outcomes**	• Quality adjusted life years (QALYs)<br>• Incremental cost-effectiveness ratio (ICER)	• Clinical outcomes
**Study Design**	• All cost-effectiveness/cost-utility studies of CAR T-cell therapies will be included	• Abstract only study<br>• Commentaries, editorials<br>• Cost only study<br>• Reviews<br>• Budget impact study<br>• Partial economic evaluations<br>• Reports
**Limits**	• English language only<br>• Studies published in peer reviewed journals only	
**Date**	• Pubmed (MeSH & free-text) 1/1/2017 - 29/1/2024<br>• Ovid Embase (free-text) 2017 - 2024<br>• Scopus (free-text) 1/1/2017 - 29/1/2024	

Table S3: CAR T-cell therapy spending by cost component (US$)

Author	Drug Acquisition CAR T (US$)	%	Administration (US$)	%	Hospitalisation (US$)	%	Bridging and lymphodepleting chemotherapy (US$)	%	Leukapheresis (US$)	%	Adverse events (US$)	%	HSCT (US$)	%
Roth et al (2018) [1]	373,000	86	$ -	0	$ -	0	$ -	0	$ -	0	$ 3,745	1	$ 19,310	4
Whittington et al (2019) Adult [2]	459,000	100	$ -	0	$ -	0	$ -	0	$ -	0	$ -	0	$ -	0
	554,000	100	$ -	0	$ -	0	$ -	0	$ -	0	$ -	0	$ -	0
Lin et al (2019) [3]	373,000	74	$ 31,000	6	$ -	0	$ 3,200	1	$ -	0	$ 25,100	5	$ 66,000	13
Liu et al (2021) [4]	373,000	55	$ 143	0	$ 168,453	25	$ 3,175	0	$ 224	0	$ 6,119	1	$ 39,518	6
Oluwale et al (2022) [5]	468,499	64	$ -	0	$ 112,972	15	$ 14,932	2	$ -	0	$ -	0	$ 49,474	7
Cummings et al (2022) [6]	399,000	57	$ 148	0	$ 57,935	8	$ 13,958	2	$ 336	0	$ 34,906	5	$ 60,221	9
	410,300	88	$ 148	0	$ 56,874	12	$ -	0	$ -	0	$ -	0	$ -	0
	373,000	87	$ 148	0	$ 56,386	13	$ -	0	$ -	0	$ -	0	$ -	0
Bastos-Oreiro (2022) [7]	516,316	83	$ -	0	$ -	0	$ 80,625	13	$ 3,404	1	$ -	0	$ 16,154	3
Perales et al (2022) [8]	399,000	44	$ 72,977	8	$ -	0	$ 5,231	1	$ 1,173	0	$ -	0	$ 399,893	45
Kambhampati et al (2022) [9]	393,104	66	$ -	0	$ -	0	$ -	0	$ -	0	$ 59,124	10	$ 139,194	24
Hillis et al (2022) [10]	395,936	61	$ 38,549	6	$ -	0	$ 1,613	0	$ 1,214	0	$ -	0	$ 10,414	2
Li et al (2022) [11]	173,978	56	$ -	0	$ -	0	$ -	0	$ -	0	$ -	0	$ 139,194	44
Potnis et al (2023) [12]	443,118	37	$ -	0	$ -	0	$ -	0	$ 395,380	33	$ 18,335	2	$ 293,787	24
Loftager et al (2023) [13]	386,242	74	$ -	0	$ 16,014	3	$ -	0	$ 914	0	$ 20,167	4	$ 85,068	16
Choe et al (2022) [14]	399,000	71	$ 2,731	0	$ 80,071	14	$ -	0	$ -	0	$ 65,826	12	$ -	0
	0	0	$ -	0	$ -	0	$ -	0	$ -	0	$ -	0	$ -	0
Wu et al (2023) [15]	298,359	72	$ -	0	$ 13,251	3	$ 7,909	2	$ -	0	$ 55,401	13	$ 29,751	7

	298,359	72	$ -	0	$ 13,251	3	$ 7,909	2	$ -	0	$ 55,401	13	$ 29,751	7
	298,359	72	$ -	0	$ 13,251	3	$ 7,909	2	$ -	0	$ 55,401	13	$ 29,751	7
Vijenthira et al (2023) [16]	399,000	80	$ 469	0	$ -	0	$ 1,519	0	$ 2,188	0	$ 85,465	17	$ -	0
	373,000	79	$ 469	0	$ -	0	$ 1,519	0	$ 2,188	0	$ 80,465	17	$ -	0
	410,300	86	$ 469	0	$ -	0	$ 1,519	0	$ 2,188	0	$ 50,393	11	$ -	0
Oluwale et al (2023) [17]	462,000	76	$ 74,068	12	$ -	0	$ 6,317	1	$ 1,190	0	$ -	0	$ 45,172	7
	447,227	75	$ 74,068	12	$ -	0	$ 6,317	1	$ 1,190	0	$ -	0	$ 45,172	8
	427,048	74	$ 74,068	13	$ -	0	$ 6,317	1	$ 1,190	0	$ -	0	$ 45,172	8
Whittington et al (2018) Paediatric [18]	405,490	67	$ -	0	$ -	0	$ 111,548	19	$ -	0	$ 33,534	6	$ 47,744	8
Lin et al (2018) [19]	475,000	79	$ 5,800	1	$ -	0	$ 16,900	3	$ -	0	$ 37,000	6	$ 66,000	11
Lin et al (2019) [3]	373,000	74	$ 31,000	6	$ -	0	$ 3,200	1	$ -	0	$ 22,700	5	$ 66,000	13
Sarkar et al (2019) [20]	475,000	88	$ -	0	$ 32,723	6	$ 3,453	1	$ -	0	$ 15,726	3	$ -	0
	$ -	0	$ -	0	$ -	0	$ -	0	$ -	0	$ -	0	$ -	0
Thielen et al (2020) [21]	418,848	74	$ -	0	$ -	0	$ -	0	$ 8,928	2	$ 32,370	6	$ 47,221	8
Cher et al (2020) [22]	370,370	65	$ -	0	$ 2,498	0	$ 3,755	1	$ 1,345	0	$ 17,495	3	$ 173,999	31
Furzer et al (2020) [23]	507,959	76	$ -	0	$ 18,927	3	$ 2,214	0	$ 8,045	1	$ 132,993	20	$ -	0
Santasusana et al (2020) [24]	413,452	72	$ -	0	$ 41,122	7	$ 45,550	8	$ -	0	$ 51,389	9	$ 19,678	3
Qi et al (2021) [25]	373,000	71	$ 12,347	2	$ 46,398	9	$ -	0	$ -	0	$ 45,092	9	$ 35,156	7
Wakase et al (2021) [26]	349,622	80	$ -	0	$ -	0	$ 7,462	2	$ -	0	$ 16,106	4	$ 58,978	13
Wakase et al (2021) [27]	349,622	88	$ -	0	$ -	0	$ 5,634	1	$ -	0	$ 4,767	1	$ 32,905	8
Moradi-Lakeh et al (2021) [28]	253,012	92	$ 607	0	$ -	0	$ -	0	$ -	0	$ 19,509	7	$ -	0
	253,012	100	$ 622	0	$ -	0	$ -	0	$ -	0	$ -	0	$ -	0
Wang et al (2021) [29]	595,238	92	$ -	0	$ 20,239	3	$ 6,504	1	$ 4,917	1	$ 11,540	2	$ 7,697	1

Wang et al (2022) [30]	595,238	85	$ -	0	$ 17,290	2	$ 28,009	4	$ 4,917	1	$ 21,876	3	$ 27,675	4
Carey et al (2022) [31]	387,371	82	$ -	0	$ 48,709	10	$ 8,172	2	$ 1,603	0	$ 24,217	5	$ -	0
Gye et al (2022) [32]	271,788	88	$ -	0	$ -	0	$ -	0	$ -	0	$ 23,841	8	$ 12,667	4
Choe et al (2022) [14]	373,000	36	$ 9,675	1	$ 21,383	2	$ 9,049	1	$ -	0	$ 25,705	2	$ 578,734	56
	373,000	36	$ 9,675	1	$ 21,383	2	$ 2,685	0	$ -	0	$ 31,022	3	$ 578,734	56
Wu et al (2023) [15]	309,547	73	$ -	0	$ 13,251	3	$ 7,909	2	$ -	0	$ 55,401	13	$ 29,751	7
	309,547	73	$ -	0	$ 13,251	3	$ 7,909	2	$ -	0	$ 55,401	13	$ 29,751	7
Simons et al (2021) [33]	373,000	69	$ 114	0	$ 75,543	14	$ 12,866	2	$ 1,437	0	$ 72,297	13	$ -	0
Ball et al (2021) [34]	304,490	79	$ 172	0	$ 43,578	11	$ 707	0	$ 1,136	0	$ 9,260	2	$ -	0
Shah et al (2022) [35]	399,000	53	$ 202,249	27	$ -	0	$ -	0	$ -	0	$ 49,172	7	$ 75,724	10
Petersohn et al (2022) [36]	464,197	88	$ -	0	$ 17,715	3	$ 9,762	2	$ 2,233	0	$ 13,452	3	$ -	0
Marchetti (2023) [37]	600,000	65	$ -	0	$ 11,035	1	$ -	0	$ 671	0	$ 103,265	10	$ 299,030	29
Wu et al (2023) [15]	309,547	73	$ -	0	$ 13,251	3	$ 7,909	2	$ -	0	$ 55,401	13	$ 29,751	7
	309,547	73	$ -	0	$ 13,251	3	$ 7,909	2	$ -	0	$ 55,401	13	$ 29,751	7
Parker et al (2023) [38]	410,300	74	$ 15,328	3	$ 3,888	1	$ 7,776	1	$ 5,964	1	$ 89,149	16	$ -	0
Kelker et al (2023) [39]	417,735	54	$ -	0	$ -	0	$ -	0	$ 1,265	0	$ 135,678	17	$ 171,892.00	22
	412,362	53	$ -	0	$ -	0	$ -	0	$ 1,265	0	$ 135,678	18	$ 171,892.00	22
	398,228	52	$ -	0	$ -	0	$ -	0	$ 1,265	0	$ 135,678	18	$ 171,892.00	23
Choe et al (2024) [40]	410,300	80	$ 536	0	$ 36,114	7	$ 8,068	2	$ 2,920	1	$ 47,682	9	$ -	0
Kapinos et al (2023) [41]	442,705	77		0	$ -	0	$ 12,981	2	$ -	0	$ 119,262	21	$ -	0
Wu et al (2023) [42]	193,023	93	$ -	0	$ -	0	$ -	0	$ -	0	$ 11,785	6	$ -	
Karampampa et al (2023) [43]	444,898	91	$ -	0	$ 10,297.14	2	$ 9,271.02	2	$ 2,161.63	0	$ 17,744	4	$ -	0

	509,772	90	$ 11,284	2	$ 3,460.77	1	$ 10,552.07	2	$ 2,014.27	0	$ 15,131	3	$ -	0
Yamamoto et al (2024) [44]	419,500	93	$ -	0	$ -	0	$ 23,308.00	5	$ 1,323.00	0	$ -	0	$ -	0
	465,000	93	$ -	0	$ -	0	$ 23,308.00	5	$ 1,323.00	0	$ -	0	$ -	0
	334,598	91	$ -	0	$ -	0	$ 14,330.06	4	$ 1,787.38	0	$ -	0	$ -	0
	334,598	91	$ -	0	$ -	0	$ 14,330.06	4	$ 1,787.38	0	$ -	0	$ -	0
Wu et al (2023) [42]	193,023	93	$ -	0	$ -	0	$ -	0	$ -	0	$ 11,785	6	$ -	0
Kapinos et al (2023) [41]	442,705	77		0	$ -	0	$ 12,981	2	$ -	0	$ 117,789	21	$ -	0
Wu et al (2023) [15]	320,736	73	$ -	0	$ 13,251	3	$ 7,909	2	$ -	0	$ 55,401	13	$ 29,751	7
Lin et al (2023) [45]	320,736	87	$ 5,362	1	$ -	0	$ -	0	$ -	0	$ 12,344	3	$ 28,158	8

**Author**	**End of Life (US$)**	**%**	**Terminal care (US$)**	**%**	**Societal Cost (US$)**	**%**	**Emergency Care (US$)**	**%**	**Outpatient (US$)**	**%**	**Total Intervention (US$)**	**Hospitalisation, adverse events and CAR T Total (US$)**	**Other %**
Roth et al (2018) [1]	$ -	0	$ 36,256	8	$ -	0	$ -	0	$ -	0	432,311	376,745	13
Whittington et al (2019) Adult [2]	$ -	0	$ -	0	$ -	0	$ -	0	$ -	0	459,000	459,000	0
	$ -	0	$ -	0	$ -	0	$ -	0	$ -	0	554,000	554,000	0
Lin et al (2019) [3]	$ -	0	$ -	0	$ -	0	$ -	0	$ 8,100	2	506,400	398,100	21
Liu et al (2021) [4]	$ -	0	$ 87,040	13	$ -	0	$ -	0	$ -	0	677,672	547,572	19
Oluwale et al (2022) [5]	$ -	0	$ 89,895	12	$ -	0	$ -	0	$ -	0	735,772	581,471	21
Cummings et al (2022) [6]	$ 136,775	19	$ -	0	$ -	0	$ 7	0	$ 2,623	0	705,909	491,841	30
	$ -	0	$ -	0	$ -	0	$ 5	0	$ -	0	467,327	467,174	0
	$ -	0	$ -	0	$ -	0	$ 6	0	$ -	0	429,540	429,386	0
Bastos-Oreiro (2022) [7]	$ 7,974	1	$ -	0	$ -	0	$ 953	0	$ -	0	625,426	516,316	17

Perales et al (2022) [8]	$ 19,406	2	$ -	0	$ -	0	$ -	0	$ -	0	897,680	399,000	56
Kambhampati et al (2022) [9]	$ -	0	$ -	0	$ -	0	$ -	0	$ -	0	591,422	452,228	24
Hillis et al (2022) [10]	$ -	0	$ 23,980	4	$ 179,789	28	$ 1,282	0	$ -	0	472,988	395,936	16
Li et al (2022) [11]	$ -	0	$ -	0	$ -	0	$ -	0	$ -	0	313,172	173,978	39
Potnis et al (2023) [12]	$ 60,525	5	$ -	0	$ -	0	$ -	0	$ -	0	1,211,145	461,453	62
Loftager et al (2023) [13]	$ 10,515	2	$ -	0	$ -	0	$ -	0	$ 381	0	519,300	422,422	19
Choe et al (2022) [14]	$ 18,076	3	$ -	0	$ -	0	$ -	0	$ -	0	565,704	544,897	4
	$ -	0	$ -	0	$ 2,579	100	$ -	0	$ -	0	2,579	0	100
Wu et al (2023) [15]	$ -	0	$ 1,231	0	$ -	0	$ -	0	$ 9,849	2	415,752	367,011	12
	$ -	0	$ 1,231	0	$ -	0	$ -	0	$ 9,849	2	415,752	367,011	12
	$ -	0	$ 1,231	0	$ -	0	$ -	0	$ 9,849	2	415,752	367,011	12
Vijenthira et al (2023) [16]	$ 12,829	3	$ -	0	$ -	0	$ -	0	$ -	0	501,470	484,465	3
	$ 12,829	3	$ -	0	$ -	0	$ -	0	$ -	0	470,470	453,465	4
	$ 12,829	3	$ -	0	$ -	0	$ -	0	$ -	0	477,698	460,693	4
Oluwale et al (2023) [5]	$ 19,696	3	$ -	0	$ -	0	$ -	0	$ -	0	608,443	462,000	24
	$ 19,696	3	$ -	0	$ -	0	$ -	0	$ -	0	593,670	447,227	25
	$ 19,696	3	$ -	0	$ -	0	$ -	0	$ -	0	573,491	427,048	26
Whittington et al (2018) Paediatric [18]	$ 1,602	0	$ 2,648	0	$ -	0	$ -	0	$ -	0	602,566	439,024	27
Lin et al (2018) [19]	$ -	0	$ 220	0	$ -	0	$ -	0	$ -	0	600,920	512,000	15
Lin et al (2019) [3]	$ -	0	$ -	0	$ -	0	$ -	0	$ 8,100	2	504,000	395,700	21
Sarkar et al (2019) [20]	$ 12,867	2	$ -	0	$ -	0	$ -	0	$ -	0	542,348	523,449	3
	$ -	0	$ -	0	$ 2,579	0	$ -	0	$ -	0	2,579	0	100
Thielen et al (2020) [21]	$ -	0	$ -	0	$ 55,730	10	$ -	0	$ -	0	563,098	451,219	20
Cher et al (2020) [22]	$ -	0	$ -	0	$ -	0	$ -	0	$ -	0	569,462	390,363	31

Furzer et al (2020) [23]	$ -	0	$ -	0	$ -	0	$ -	0	$ -	0	670,137	659,878	2
Santasusana et al (2020) [24]	$ -	0	$ 6,845	1	$ -	0	$ -	0	$ -	0	578,037	505,963	12
Qi et al (2021) [25]	$ -	0	$ 15,516	3	$ -	0	$ -	0	$ -	0	527,509	464,490	12
Wakase et al (2021) [27]	$ -	0	$ 5,730	1	$ -	0	$ -	0	$ -	0	437,897	365,728	16
Wakase et al (2021) [26]	$ -	0	$ 6,636	2	$ -	0	$ -	0	$ -	0	399,563	354,389	11
Moradi-Lakeh et al (2021) [28]	$ -	0	$ 1,933	1	$ -	0	$ -	0	$ -	0	275,061	272,521	1
	$ -	0	$ -	0	$ -	0	$ -	0	$ -	0	253,634	253,012	0
Wang et al (2021) [29]	$ -	0	$ -	0	$ -	0	$ -	0	$ -	0	646,134	627,017	3
Wang et al (2022) [30]	$ -	0	$ 3,823	1	$ -	0	$ -	0	$ -	0	698,827	634,404	9
Carey et al (2022) [31]	$ -	0	$ -	0	$ -	0	$ -	0	$ -	0	470,072	460,296	2
Gye et al (2022) [32]	$ -	0	$ -	0	$ -	0	$ -	0	$ -	0	308,296	295,629	4
Choe et al (2022) [14]	$ 18,076	2	$ -	0	$ 2,579	0	$ -	0	$ -	0	1,035,622	420,088	60
	$ 18,076	2	$ -	0	$ 2,579	0	$ -	0	$ -	0	1,034,575	425,405	59
Wu et al (2023) [15]	$ -	0	$ 1,231	0	$ -	0	$ -	0	$ 9,849	2	426,941	378,200	11
	$ -	0	$ 1,231	0	$ -	0	$ -	0	$ 9,849	2	426,941	378,200	11
Simons et al (2021) [33]	$ 5,394	1	$ -	0	$ -	0	$ -	0	$ -	0	540,651	520,840	4
Ball et al (2021) [34]	$ 24,149	6	$ -	0	$ -	0	$ -	0	$ -	0	383,492	357,328	7
Shah et al (2022) [35]	$ 22,368	3	$ -	0	$ -	0	$ -	0	$ -	0	748,513	448,172	40
Petersohn et al (2022) [36]	$ 17,756	3	$ -	0	$ -	0	$ -	0	$ -	0	525,116	495,364	6
Marchetti (2023) [37]	$ -	0	$ 8,553	1	$ -	0	$ -	0	$ -	0	1,022,554	714,300	30
Wu et al (2023) [15]	$ -	0	$ 1,231	0	$ -	0	$ -	0	$ 9,849	2	426,941	378,200	11
	$ -	0	$ 1,231	0	$ -	0	$ -	0	$ 9,849	2	426,941	378,200	11

Parker et al (2023) [38]	$ 10,716	2	$ -	0	$ -	0	$ -	0	$ 11,441	2	554,562	503,337	9
Kelker et al (2023) [39]	$ -	0	$ -	0	$ -	0	$ -	0	$ 53,360	7	779,930	553,413	29
	$ -	0	$ -	0	$ -	0	$ -	0	$ 53,360	7	774,557	548,040	29
	$ -	0	$ -	0	$ -	0	$ -	0	$ 53,360	7	760,423	533,906	30
Choe et al (2024) [40]	$ -	0	$ -	0	$ 8,161	2	$ -	0	$ -	0	513,781	494,096	4
Kapinos et al (2023) [41]	$ -	0	$ -	0	$ -	0	$ -	0	$ -	0	574,948	561,967	2
Wu et al (2023) [42]	$ -	0	$ 3,039	1	$ -	0	$ -	0	0	0	207,847	204,808	1
Karampampa et al (2023) [43]	$ -	0	$ 3,607	1	$ 1,915	0	$ -	0	0	0	489,173	472,939	3
	$ -	0	$ 10,810	2	$ 1,803	0	$ -	0	0	0	564,827	528,364	6
Yamamoto et al (2024) [44]	$ 9,175	2	$ -	0	$ -	0	$ -	0	0	0	453,306	419,500	7
	$ 9,175	2	$ -	0	$ -	0	$ -	0	0	0	498,806	465,000	7
	$ 15,702	4	$ -	0	$ -	0	$ -	0	0	0	366,418	334,598	9
	$ 15,702	4	$ -	0	$ -	0	$ -	0	0	0	366,418	334,598	9
Wu et al (2023) [42]	$ -	0	$ 3,039	1	$ -	0	$ -	0	0	0	207,847	204,808	1
Kapinos et al (2023) [41]	$ -	0	$ -	0	$ -	0	$ -	0	$ -	0	573,475	560,494	2
Wu et al (2023) [15]	$ -	0	$ 1,231	0	$ -	0	$ -	0	$ 9,849	2	438,129	389,388	11
Lin et al (2023) [45]	$ 3,754	1	$ -	0	$ -	0	$ -	0	$ -	0	370,353	333,080	10

Table S4: Payer perspective comparisons including head-to-head CAR T-cell therapy comparisons

Author (year)	CAR T ($) (Intervention)	Line of therapy	Comparator	Intervention (US$)	Comparator (US$)	Δ US$	Intervention QALYs	Comparator QALYs	Δ QALYs	Cost per QALY gained (US$)	Cost-effective (%)	WTP (US$)
**Axicabtagene Ciloleucel**												
Roth et al (2018) [1]	373,000	Not specified	SC	552,921	172,737	380,184	7.67	1.13	6.54	58,146	90.0%	100,000
Lin et al (2019) [19]	373,000	Not specified	SC + SCT	651,000	169,000	482,000	5.5	1.78	3.72	129,000	73.0%	150,000

Perales et al (2022) [8]	399,000	2L	SC	635,794	535,428	100,366	7.08	5.56	1.52	66,381	75.0%	150,000
Kambhampati et al (2022) [9]	393,104	2L	SC	771,838	508,034	263,804	5.42	2.6	2.82	93,547	73.0%	150,000
Hillis et al (2022) [10]	395,936	≥2L	Chemo	502,894	106,704	396,190	7.71	3.15	4.56	86,851	92.0%	150,000
Li et al (2022) [11]	173,978	≥2L	SC	198,070	22,690	175,380	3.08	0.47	2.61	67,251	99.0%	200,000
Potnis et al (2023) [12]	443,118	3L	SC	731,682	458,490	273,192	7.04	5.54	1.5	182,127	4.0%	150,000
Loftager et al (2023) [13]	386,242	2L	Chemo	472,860	379,963	92,897	7.51	5.99	1.52	61,102	73.0%	114,273
Choe et al (2022) [14]	399,000	2L	SC + ASCT	678,903	619,149	59,754	4.53	3.93	0.6	99,101	44.0%	100,000
Wu et al (2023) [15]	298,359	1L	SC	339,867	52,913	286,954	8.4	7.86	0.54	528,421	0.0%	191,875
	298,359	2L	SC + ASCT	329,737	197,849	131,889	5.03	3.58	1.45	90,497	0.0%	191,875
	298,359	≥3L	SC	345,696	63,758	281,939	5.39	2.11	3.28	86,029	0.0%	191,875
Vijenthira et al (2023) [16]	399,000	Pol-R-CHP + 2L CAR-T	R-CHOP plus 2L SC +/- ASCT	498,243	283,398	214,845	9.61	9.17	0.44	488,284	0.0%	150,000
	399,000	R-CHOP plus 2L CAR-T	R-CHOP plus 2L SC +/- ASCT	332,968	283,398	49,570	9.33	9.17	0.16	309,813	0.1%	150,000
	399,000	Pol-R-CHP + 2L CAR-T	R-CHOP plus 2L SC +/- ASCT	204,092	108,172	95,920	9.61	9.17	0.44	218,000	0.0%	150,000
	399,000	R-CHOP plus 2L CAR-T	R-CHOP plus 2L SC +/- ASCT	147,769	108,172	39,597	9.33	9.17	0.16	247,480	0.0%	150,000
Kelker et al (2023) [39]	417,735	2L	SC + ASCT	537,361	385,260	152,101	1.82	1.6	0.22	684,225	20.0%	200,000
Oluwale et al (2023) [17]	462,000	2L	SC + ASCT	769,890	609,981	159,909	7.23	5.6	1.63	98,040	82.0%	150,000
Liu et al (2021) [4]	373,000	≥2L	Tis	586,313	587,720	-1,407	7.47	5.16	2.31	-609	95.0%	31,500
Oluwale et al (2022) [5]	468,499	≥2L	LM	611,440	597,174	14,266	7.76	5.94	1.82	7,843	93.0%	50,000
Cummings et al (2022) [6]	399,000	≥2L	LM	637,129	620,962	16,167	7.705	5.898	1.807	8,946	100.0%	150,000
	399,000	≥2L	Tis	631,331	576,563	54,768	7.24	5.005	2.235	24,506	100.0%	150,000
Bastos-Oreiro (2022) [7]	516,316	≥2L	Tis	708,465	658,901	81,520	7.47	5.16	2.31	1,003	92.3%	36,184
**Tisagenlecleucel**												
Lin et al (2018) [19]	475,000	Not specified	Blin	599,000	282,000	317,000	8.74	3.57	5.17	61,315	98%	100,000
Lin et al (2019) [3]	373,000	Not specified	SC + SCT	529,000	169,000	360,000	3.92	1.78	2.14	168,224	33.0%	150,000
Sarkar et al (2019) [20]	475,000	Not specified	SC	528,200	440,600	87,600	16.76	8.58	8.18	64,601	94.8%	100,000
Qi et al (2021) [25]	373,000	≥2L	SC	588,080	324,319	263,761	5.29	1.94	3.35	78,652	91.9%	150,000
Furzer et al (2020) [23]	507,959	2L	SC + ASCT	477,551	93,061	384,490	5.14	3.46	1.68	383,685	32.0%	122,449
Wakase et al (2021) [26]	349,622	Not specified	Blin	412,782	235,478	177,304	11.6	3.1	8.5	20,857	100.0%	51,149
		Not specified	CC	412,782	153,592	259,189	11.6	2.1	8.5	27,105	100.0%	51,149
Wakase et al (2021) [27]	349,622	≥3L	SC	369,813	210,032	159,781	5.42	2.57	2.85	56,127	80.0%	51,149

Thielen et al (2020) [21]	418,848	1L	Blin	723,402	349,815	273,914	11.26	2.25	9.01	34,188	98.0%	104,712
Moradi-Lakeh et al (2021) [28]	253,012	≥2L	CC (pALL)	340,838	188,008	152,830	8.29	1.64	6.65	22,989	100.0%	107,581
		≥2L	SC (pALL)	340,838	172,813	168,025	8.29	2.51	5.78	21,279	100.0%	107,581
		≥2L	Blin (pALL)	340,838	190,143	150,695	8.29	2.07	6.22	24,247	100.0%	107,581
		≥2L	SC (DLBCL)	268,622	98,292	170,328	4.77	2.51	2.26	75,352	86.0%	66,578
Cher et al (2020) [22]	370,370	≥2L	SC	297,911	39,536	307,589	2.064	1.556	0.508	508,530	0.0%	280,000
Wang et al (2021) [29]	595,238	≥2L	SC	616,252	626,343	10,090	5.6655	2.8905	2.775	-10,090	100.0%	317,825
Wang et al (2022) [30]	595,238	≥2L	SC	713,005	174,532	538,473	10.6	0.73	9.87	54,571	100.0%	317,825
	595,239	≥2L	Blin	713,005	249,101	350,885	10.6	3.1	7.5	61,879	100.0%	317,825
Carey et al (2022) [31]	387,371	≥2L	Blin	483,797	282,349	201,448	4.33	2.18	2.15	93,820	16.0%	57,766
Choe et al (2022) [40]	373,000	2L	SC + ASCT	534,426	496,623	37,803	2.02	2.04	-0.02	-130,355	9.0%	100,000
	373,000	≥3L	SC + ASCT	489,767	218,368	271,399	3.86	1.72	2.14	126,593	9.0%	100,000
Wu et al (2023) [15]	309,547	2L	SC + ASCT	353,865	146,532	207,333	2.34	2.32	0.02	7,972,845	0.0%	191,875
	309, 547	3L	SC	360,350	63,758	296,592	3.93	2.11	1.82	162,963	0.0%	191.875
**Brexucabtagene Autoleucel**												
Simons et al (2021) [33]	373,000	Not specified	Cyto chemo, PI, IMD, Bcl-2, BTK	693,832	574,263	119,569	7.39	3.65	3.74	31,985	94.0%	100,000
Ball et al (2021) [34]	304,490	≥2L	BSC	570,777	53,753	517,841	8.34	1.31	7.03	72,247	82.0%	81,633
Shah et al (2022) [35]	399,000	Not specified	Blin	776,320	725,407	50,913	5.95	4.92	1.03	20,843	78.4%	150,000
	399,000	Not specified	Ino	776,320	524,789	251,531	5.95	2.69	3.26	77,271	74.0%	150,000
	399,000	Not specified	SC	776,320	344,293	432,027	5.95	2.17	3.78	93,768	75.4%	150,000
Marchetti (2023) [37]	600,000	Not specified	R-BAC	685,672	124,025	561,647	6.4	1.2	5.2	107,997	88%	£145,550
**Lisocabtagene Maraleucel**												
Wu et al (2023) [15]	309,547	2L	SC + ASCT	348,128	198,826	149,302	4.73	3.01	1.72	86,225	0.0%	191,875
	309,547	2L	SC	351,235	61,418	289,817	2.83	1.88	0.95	306,807	0.0%	191,875
	309,547	≥3L	SC	347,072	63,758	283,314	4.72	2.11	2.61	108,617	0.0%	191,875
Kelker et al (2023) [39]	412,362	2L	SC + ASCT	547,951	424,386	123,565	1.41	1.31	0.1	1,171,909	19.0%	200,000
Choe et al (2024) [40]	410,300	2L	SCare chemo + ASCT	668,624	467,624	201,001	3.64	1.62	2.02	99,669	54.0%	100,000
Parker et al (2023) [38]	410,300	≥3L	AC	440,106	515,085	-74,980	5.09	5.09	0.002	-37,490,000	82.0%	100,000
	410,300	≥3L	Tis	440,106	372,180	67,926	5.09	3.07	2.02	33,627	96.0%	100,000

**Idecabtagene Vicleucel**												
Wu et al (2023) [42]	193,023	≥4L	SC	217,205	76,512	140,693	2.11	0.92	1.19	118,229	0.0%	37,653
**Ciltacabtagene Autoleucel**												
Kapinos et al (2023) [41]	465,000	Not specified	ADC	477,980	65,428	412,552	4.29	0.52	3.77	109,497	50.0%	123,618
Wu et al (2023) [42]	193,023	≥4L	SC	196,318	75,512	119,806	4.23	0.92	3.31	36,195	72.0%	37,653
**Relmacabtagene Autoleucel**												
Wu et al (2023) [15]	320,736	≥3L	SC	358,909	63,758	295,151	6.34	2.11	4.23	69,857	0.0%	£191,875
Lin et al (2023) [45]	320,736	2L	SC	400,022	134,624	265,398	6.67	1.41	5.26	50,506	74.0%	£60,400

*Where two perspectives are given and one is societal, we took the payer perspective. Excluded studies without reporting of cost-effective (%) or WTP (US$) but includes CAR T V CAR T comparisons, n = 63

Table S5: Multiple linear regression analysis, relationship between cost per QALY gained, treatment-line and predictor variables

Regression 1

Source	SS	df	MS	Number of obs	=	52
				F(32, 19)	=	1.06
**Model**	3.9834E+13	32	1.2448E+12	Prob > F	=	0.4543
**Residual**	2.2225E+13	19	1.1698E+12	R-squared	=	**0.6419**
				Adj R-squared	=	0.0387
**Total**	6.2059E+13	51	1.2168E+12	Root MSE	=	1.10E+06

Cost_per_QALY	Coef.	Std. Err.	t	P>t	(95% Conf. Interval)	
**CART**						
Kymriah_Tisagenlecleucel	1777472	909379.1	1.95	0.066	-125880.2	3680825
Tecartus_Brexucabtagene Autoleucel	1436880	1588805	0.9	0.377	-1888526	4762287
Breyanzi_Lisocabtagene Maraleucel	-1135937	807445.3	-1.41	0.176	-2825939	554065.6
Abecma_Idecabtagene Vicleucel	1034746	1496728	0.69	0.498	-2097941	4167433
Carvykti_Ciltacabtagene Autoleucel	2519610	1447473	1.74	0.098	-509985.8	5549206
Carteyva_Relmacabtagene Autoleucel	-2313755	1441102	-1.61	0.125	-5330016	702505.6
**Efficacy_year**						
2-3 years	-1620694	946593.9	-1.71	0.103	-3601938	360549.6
>3 years	-1708137	803925.7	-2.12	0.047	-3390773	-25501.5
**CART_price**	-4.434422	6.815149	-0.65	0.523	-18.69869	9.82985
**Line_1**						
2L	778732.2	1370769	0.57	0.577	-2090320	3647784
>=2L	-856248.3	1467186	-0.58	0.566	-3927104	2214607
3L	-2771114	1854123	-1.49	0.151	-6651839	1109610
>=3L	103268.5	1356503	0.08	0.94	-2735924	2942461

>=4L	-2507098	1989611	-1.26	0.223	-6671402	1657206
**Country**						
Netherland	-2493139	2544753	-0.98	0.34	-7819369	2833091
Switzerland	-2700529	2438610	-1.11	0.282	-7804598	2403540
Singapore	-178124.8	2176764	-0.08	0.936	-4734144	4377894
Ireland	-2772440	1875632	-1.48	0.156	-6698182	1153303
Canada	-775718.7	1096188	-0.71	0.488	-3070066	1518628
United States	-1015629	964975	-1.05	0.306	-3035345	1004087
Sweden	-534998.9	1635710	-0.33	0.747	-3958580	2888582
Japan	-2218160	989250.2	-2.24	0.037	-4288684	-147635.3
France	64517.58	1873670	0.03	0.973	-3857119	3986154
United Kingdom	47116.88	2542916	0.02	0.985	-5275267	5369500
**Funder**						
Public	-764839.9	789164.3	-0.97	0.345	-2416580	886900
Non-profit organisation	-1839346	932618.1	-1.97	0.063	-3791338	112646.3
No statement	0	(omitted)				
**Population**						
Paediatric	1077480	2137205	0.5	0.62	-3395742	5550703
Paediatric & young adult	424538	1379299	0.31	0.762	-2462368	3311444
Paediatric & adult	396646.1	1912357	0.21	0.838	-3605963	4399256
**Cancer_type**						
Diffuse large B cell lymphoma (DLBCL)	998740.9	708866.7	1.41	0.175	-484934.1	2482416
Mantle cell lymphoma	0	(omitted)				
Follicular lymphoma (FL)	4403715	2062071	2.14	0.046	87751.74	8719678
Acute lymphoblastic leukaemia (ALL)	0	(omitted)				
Acute lymphoblastic leukaemia (ALL) & Adult Diffuse Large B-Cell Lymphoma	995772.9	1685828	0.59	0.562	-2532707	4524252
Multiple myeloma	0	(omitted)				

**_cons**	3238264	3085306	1.05	0.307	-3219355	9695884

Regression1: n = 52 comparisons. Where two perspectives are given and one is societal, we took the payer perspective. Excludes CAR T v CAR T comparisons and unspecified treatment-line. *Line of treatment taken from published paper.*

Regression 2

**Source**	**SS**	**df**	**MS**	**Number of obs**	=	52
				**F(30, 21)**	=	0.73
**Model**	3.176E+13	30	1.0588E+12	**Prob > F**	=	0.7854
**Residual**	3.0295E+13	21	1.4426E+12	**R-squared**	=	**0.5118**
				**Adj R-squared**	=	-0.1856
**Total**	6.21E+13	51	1.2168E+12	**Root MSE**	=	1.20E+06

**Cost_per_QALY**	**Coef.**	**Std. Err.**	**t**	**P>t**	**(95% Conf. Interval)**	
**CART**						
Kymriah_Tisagenlecleucel	1018557	927019.3	1.1	0.284	-909285.6	2946399
Tecartus_Brexucabtagene Autoleucel	293430.7	1653253	0.18	0.861	-3144696	3731558
Breyanzi_Lisocabtagene Maraleucel	-1206201	894852.7	-1.35	0.192	-3067149	654746.9
Abecma_Idecabtagene Vicleucel	171442.7	1593639	0.11	0.915	-3142710	3485596
Carvykti_Ciltacabtagene Autoleucel	1735238	1572628	1.1	0.282	-1535220	5005696
Carteyva_Relmacabtagene Autoleucel	-2532564	1527343	-1.66	0.112	-5708848	643719.4
**Efficacy_year**						
2-3 years	-1973390	969465.1	-2.04	0.055	-3989503	42722.8
>3 years	-1925350	883688.5	-2.18	0.041	-3763081	-87619.64
**CART_price**	1.572906	7.048216	0.22	0.826	-13.08466	16.23047

**Line_2**						
2	447343.9	1477020	0.3	0.765	-2624286	3518974
3	-264723.6	1496417	-0.18	0.861	-3376693	2847246
4	-1749569	2184688	-0.8	0.432	-6292876	2793737
**Country**						
Netherland	-2391700	2667391	-0.9	0.38	-7938844	3155444
Switzerland	-1991472	2668565	-0.75	0.464	-7541057	3558113
Singapore	-2047684	2116911	-0.97	0.344	-6450041	2354673
Ireland	-3610483	1920847	-1.88	0.074	-7605102	384136.4
Canada	-1135447	1127320	-1.01	0.325	-3479836	1208943
United States	-1369460	1008129	-1.36	0.189	-3465979	727058.9
Sweden	-1029789	1749572	-0.59	0.562	-4668223	2608646
Japan	-1686983	1015902	-1.66	0.112	-3799666	425701.4
France	-136968.4	2039873	-0.07	0.947	-4379117	4105181
United Kingdom	-1407506	2606764	-0.54	0.595	-6828569	4013557
**Funder**						
Public	-404846.8	823933.6	-0.49	0.628	-2118310	1308617
Non-profit organisation	-1446627	1021658	-1.42	0.171	-3571281	678025.8
No statement	0	(omitted)				
**Population**						
Paediatric	712900.8	2341638	0.3	0.764	-4156802	5582603
Paediatric & young adult	-332271.1	1463478	-0.23	0.823	-3375740	2711197
Paediatric & adult	-360162.9	2075025	-0.17	0.864	-4675414	3955088
**Cancer_type**						
Diffuse large B cell lymphoma (DLBCL)	475992.2	742330.6	0.64	0.528	-1067769	2019753
Mantle cell lymphoma	0	(omitted)				

Follicular lymphoma (FL)	1083302	1758649	0.62	0.545	-2574009	4740614
Acute lymphoblastic leukaemia (ALL)	0	(omitted)				
Acute lymphoblastic leukaemia (ALL) & Adult Diffuse Large B-Cell Lymphoma	473024.1	1853736	0.26	0.801	-3382032	4328080
Multiple myeloma	0	(omitted)				
**_cons**	1961376	3352723	0.59	0.565	-5010993	8933745

Regression 2: n = 52 comparisons. Where two perspectives are given and one is societal, we took the payer perspective. Excludes CAR T v CAR T comparisons and unspecified treatment-line. *Line of treatment adjusted.*

Table S6: Consolidated health economic evaluation reporting standards (CHEERS) checklist 2022

Section item	Item	Roth et al (2018)	Whittington et al (2018)
**Title**			
Title	1	Front page	Front page
**Abstract**			
Abstract	2	Front page	Front page
**Introduction**			
Background and objectives	3	Introduction	Introduction
**Methods**			
Health economic analysis plan	4	Not reported	Not reported
Study population	5	Patient population and Model Structure	Intervention and Comparator
Setting and location	6	Introduction	Model Inputs
Comparators	7	Introduction	Intervention and Comparator
Perspective	8	Overview	Study Design
Time horizon	9	Overview	Study Design
Discount rate	10	Overview	Discount Rate
Selection of outcomes	11	Overview	Sensitivity Analyses
Measurement of outcomes	12	Outcomes	Sensitivity Analyses
Valuation of outcomes	13	Health state utilities	Not reported
Measurement and valuation of resources and costs	14	Resource use and cost	Model Inputs, Table 1
Currency, price date, and conversion	15	Overview	Model Inputs
Rationale and description of model	16	Patient population and Model Structure	Study Design
Analytics and assumptions	17	Survival	Study Design
Characterizing heterogeneity	18	Not reported	Not reported
Characterizing distributional effects	19	Not reported	Not reported
Characterizing uncertainty	20	Scenario, threshold and Uncertainty Analyses	Sensitivity Analysis
Approach to engagement with patients and others affected by the study	21	N/A	N/A
**Results**			
Study parameters	22	Table 1	Table 1

Summary of main results	23	Results	Results
Effect of uncertainty	24	Results	Results
Effect of engagement with patients and others affected by the study	25	N/A	N/A
**Discussion**			
Study findings, limitations, generalizability, and current knowledge	26	Discussion	Discussion
**OTHER RELEVANT INFORMATION**			
Source of funding	27	Declaration of funding	Funding/Support
Conflicts of interest	28	Not reported	Conflict of Interest Disclosures

Section item	Item	Lin (2018)	Whittington et al (2019)
**Title**			
Title	1	Front page	Front page
**Abstract**			
Abstract	2	Front page	Front page
**Introduction**			
Background and objectives	3	Introduction	Introduction
**Methods**			
Health economic analysis plan	4	Not reported	Not reported
Study population	5	Model Structure	Study Design
Setting and location	6	Costs and Utilities	Study Design
Comparators	7	Treatment Strategies	Study Design
Perspective	8	Costs and Utilities	Cost-effectiveness Model Structure
Time horizon	9	Main Outcomes	Study Design
Discount rate	10	Main Outcomes	Study Design
Selection of outcomes	11	Main Outcomes	Study Design
Measurement of outcomes	12	Main Outcomes	Study Design
Valuation of outcomes	13	Costs and Utilities	Supplement
Measurement and valuation of resources and costs	14	Costs and Utilities, Table 1	Supplement

Currency, price date, and conversion	15	Costs and Utilities	Supplement
Rationale and description of model	16	Model Structure	Supplement
Analytics and assumptions	17	Methods	Supplement
Characterizing heterogeneity	18	Not reported	Not reported
Characterizing distributional effects	19	Not reported	Not reported
Characterizing uncertainty	20	Sensitivity Analyses	Not reported
Approach to engagement with patients and others affected by the study	21	N/A	N/A
**Results**			
Study parameters	22	Table 1	Supplement
Summary of main results	23	Base-Case Analysis	Results, Table 2 and 3
Effect of uncertainty	24	Sensitivity Analyses	Not reported
Effect of engagement with patients and others affected by the study	25	N/A	N/A
**Discussion**			
Study findings, limitations, generalizability, and current knowledge	26	Discussion	Discussion
**OTHER RELEVANT INFORMATION**			
Source of funding	27	Research Funding	Article information
Conflicts of interest	28	Authors' disclosures of potential conflicts of interest	Article information

**Section item**	**Item**	**Lin et al (2019)**	**Sarkar et al (2019)**
**Title**			
Title	1	Front page	Front page
**Abstract**			
Abstract	2	Front page	Front page
**Introduction**			
Background and objectives	3	Introduction	Introduction
**Methods**			
Health economic analysis plan	4	Not reported	Not reported
Study population	5	Model Structure	Patient Population and Treatment

Setting and location	6	Model Structure	Patient Population and Treatment
Comparators	7	Treatment Strategies	Analysis
Perspective	8	Costs and Utilities	Analysis
Time horizon	9	Model Structure	Analysis
Discount rate	10	Main Outcomes	Analysis
Selection of outcomes	11	Main Outcomes	Outcomes Measures
Measurement of outcomes	12	Supplement	Outcomes Measures
Valuation of outcomes	13	Costs and Utilities	Outcomes Measures
Measurement and valuation of resources and costs	14	Costs and Utilities	Costs
Currency, price date, and conversion	15	Costs and Utilities	Costs
Rationale and description of model	16	Model Structure	Cost-Effectiveness Model
Analytics and assumptions	17	Supplement	Supplement
Characterizing heterogeneity	18	Not reported	Not reported
Characterizing distributional effects	19	Not reported	Not reported
Characterizing uncertainty	20	Sensitivity Analyses	Analysis
Approach to engagement with patients and others affected by the study	21	N/A	N/A
**Results**			
Study parameters	22	Table 1	Table 1
Summary of main results	23	Base Case Analysis	Results
Effect of uncertainty	24	Sensitivity Analyses	Results, Figure 4
Effect of engagement with patients and others affected by the study	25	N/A	N/A
**Discussion**			
Study findings, limitations, generalizability, and current knowledge	26	Discussion	Discussion
**OTHER RELEVANT INFORMATION**			
Source of funding	27	Author contributions	Funding
Conflicts of interest	28	Authors' disclosures of potential conflicts of interest and data availability statement	Notes

**Section item**	**Item**	**Cher et al (2020)**	**Furzer et al (2020)**

**Title**			
Title	1	Front page	Front page
**Abstract**			
Abstract	2	Front page	Front page
**Introduction**			
Background and objectives	3	Background	Introduction
**Methods**			
Health economic analysis plan	4	Not reported	Not reported
Study population	5	Model structure	Introduction
Setting and location	6	Model structure	Introduction
Comparators	7	Treatment strategies	Current Standard, Care Strategy and Tisagenlecleucel Strategy
Perspective	8	Background	Introduction
Time horizon	9	Model structure	Introduction
Discount rate	10	Utility values	Methods
Selection of outcomes	11	Utility values	Methods
Measurement of outcomes	12	Additional analyses	Methods
Valuation of outcomes	13	Utility values	Utilities
Measurement and valuation of resources and costs	14	Supplement	Costs
Currency, price date, and conversion	15	Costs	Table 1
Rationale and description of model	16	Model structure	Methods
Analytics and assumptions	17	Supplement	Model Input Data
Characterizing heterogeneity	18	Not reported	Not reported
Characterizing distributional effects	19	Not reported	Not reported
Characterizing uncertainty	20	Sensitivity analyses	Results
Approach to engagement with patients and others affected by the study	21	N/A	N/A
**Results**			
Study parameters	22	Supplement	Table 1
Summary of main results	23	Base-case analysis	Results, Table 2
Effect of uncertainty	24	Sensitivity analyses	Results

Effect of engagement with patients and others affected by the study	25	N/A	N/A
**Discussion**			
Study findings, limitations, generalizability, and current knowledge	26	Discussion	Discussion
**OTHER RELEVANT INFORMATION**			
Source of funding	27	Declaration of financial/other interests	Article Information
Conflicts of interest	28	Declaration of financial/other interests	Article Information

**Section item**	**Item**	**Maria et al (2020)**	**Thielen et al (2020)**
**Title**			
Title	1	Front page	Front page
**Abstract**			
Abstract	2	Front page	Front page
**Introduction**			
Background and objectives	3	Introduction	Introduction
**Methods**			
Health economic analysis plan	4	Not reported	Not reported
Study population	5	Patients	Introduction
Setting and location	6	Materials and Methods	Methods
Comparators	7	Treatments	Methods
Perspective	8	Materials and Methods	Methods
Time horizon	9	Materials and Methods	Methods
Discount rate	10	Materials and Methods	Methods
Selection of outcomes	11	Effectiveness Measures	Methods
Measurement of outcomes	12	Effectiveness Measures	Methods
Valuation of outcomes	13	Effectiveness Measures	Methods
Measurement and valuation of resources and costs	14	Resource Use and Health Costs	Supplemental and Table 1
Currency, price date, and conversion	15	Resource Use and Health Costs	Methods and Supplement
Rationale and description of model	16	Analysis	Methods
Analytics and assumptions	17	Supplement	Supplement

Characterizing heterogeneity	18	Not reported	Not reported
Characterizing distributional effects	19	Not reported	Not reported
Characterizing uncertainty	20	Sensitivity Analysis	Results
Approach to engagement with patients and others affected by the study	21	N/A	N/A
**Results**			
Study parameters	22	Supplement	Table 2
Summary of main results	23	Results of the Base Case	Results
Effect of uncertainty	24	Results of the Sensitivity Analysis	Results
Effect of engagement with patients and others affected by the study	25	N/A	N/A
**Discussion**			
Study findings, limitations, generalizability, and current knowledge	26	Discussion	Discussion
**OTHER RELEVANT INFORMATION**			
Source of funding	27	Disclosure	Conflict of Interest
Conflicts of interest	28	Disclosure	Conflict of Interest

**Section item**	**Item**	**Liu et al (2021)**	**Ball et al (2021)**
**Title**			
Title	1	Front page	Front page
**Abstract**			
Abstract	2	Front page	Front page
**Introduction**			
Background and objectives	3	Introduction	Introduction
**Methods**			
Health economic analysis plan	4	Not reported	Not reported
Study population	5	Introduction	Target Population
Setting and location	6	Methods	Materials and Methods
Comparators	7	Methods	Comparators
Perspective	8	Methods	Model perspective
Time horizon	9	Methods	Time Horizon, Discounting Cycle Length

Discount rate	10	Methods	Time Horizon, Discounting Cycle Length
Selection of outcomes	11	Methods	Partitioned Survival Model
Measurement of outcomes	12	Methods	Materials and Methods, Table 1
Valuation of outcomes	13	Utilities	Utility Values
Measurement and valuation of resources and costs	14	Costs, Table 1	Table A2
Currency, price date, and conversion	15	Methods	Cost and Resource Use
Rationale and description of model	16	Model structure	Model Structure and Approach
Analytics and assumptions	17	Model inputs	Materials and Methods
Characterizing heterogeneity	18	Model inputs	Partitioned Survival Mixture Cure Model
Characterizing distributional effects	19	Not reported	Not reported
Characterizing uncertainty	20	Sensitivity analyses	Not reported
Approach to engagement with patients and others affected by the study	21	N/A	N/A
**Results**			
Study parameters	22	Table 1	Table A8
Summary of main results	23	Base case	Deterministic Analysis
Effect of uncertainty	24	Probabilistic sensitivity analysis	Probabilistic Sensitivity Analysis
Effect of engagement with patients and others affected by the study	25	N/A	N/A
**Discussion**			
Study findings, limitations, generalizability, and current knowledge	26	Discussion	Discussion
**OTHER RELEVANT INFORMATION**			
Source of funding	27	Declaration of funding	Funding
Conflicts of interest	28	Declaration of financial/other relationships	Conflicts of Interest

Section item	Item	Simons et al (2021)	Moradi-Lakeh et al (2021)
**Title**			
Title	1	Front page	Front page
**Abstract**			
Abstract	2	Front page	Front page
**Introduction**			

Background and objectives	3	Introduction	Introduction
**Methods**			
Health economic analysis plan	4	Not reported	Not reported
Study population	5	Overview	Introduction
Setting and location	6	Overview	Introduction
Comparators	7	Overview	Intervention Technology and Comparators
Perspective	8	Overview	Overview of Models (pALL and DLBCL)
Time horizon	9	Overview	Overview of Models (pALL and DLBCL)
Discount rate	10	Overview	Overview of Models (pALL and DLBCL)
Selection of outcomes	11	Overview	Overview of Models (pALL and DLBCL)
Measurement of outcomes	12	Overview	Overview of Models (pALL and DLBCL)
Valuation of outcomes	13	Utilities	Utility Inputs
Measurement and valuation of resources and costs	14	Cost and resource use, Table 1	Supplemental
Currency, price date, and conversion	15	Overview	Economic Inputs
Rationale and description of model	16	Mode Structure	Model Structure
Analytics and assumptions	17	Methods	Supplemental
Characterizing heterogeneity	18	Not reported	Not reported
Characterizing distributional effects	19	Not reported	Not reported
Characterizing uncertainty	20	Sensitivity analyses	Sensitivity Analyses
Approach to engagement with patients and others affected by the study	21	N/A	N/A
**Results**			
Study parameters	22	Table 1	Supplemental
Summary of main results	23	Base case results, Table 2	Results, Table 2
Effect of uncertainty	24	Sensitivity analyses	Results
Effect of engagement with patients and others affected by the study	25	N/A	N/A
**Discussion**			
Study findings, limitations, generalizability, and current knowledge	26	Discussion	Discussion
**OTHER RELEVANT INFORMATION**			

Source of funding	27	Declaration of funding	Funding
Conflicts of interest	28	Declaration of financial/other interests	Disclosures

**Section item**	**Item**	**Qi et al (2021)**	**Wakase et al (2021)**
**Title**			
Title	1	Front page	Front page
**Abstract**			
Abstract	2	Front page	Front page
**Introduction**			
Background and objectives	3	Introduction	Introduction
**Methods**			
Health economic analysis plan	4	Not reported	Not reported
Study population	5	Model Overview	Introduction
Setting and location	6	Model Overview	Introduction
Comparators	7	Model Overview	Model Overview and Structure
Perspective	8	Model Overview	Model Overview and Structure
Time horizon	9	Model Overview	Model Overview and Structure
Discount rate	10	Model Overview	Model Overview and Structure
Selection of outcomes	11	Model Outputs	Model Outputs
Measurement of outcomes	12	Model Overview	Model Outputs
Valuation of outcomes	13	Model Overview	Utility Inputs
Measurement and valuation of resources and costs	14	Cost Inputs, Table 1	Table 1
Currency, price date, and conversion	15	Not reported	Cost Inputs
Rationale and description of model	16	Model Overview	Model Overview and Structure
Analytics and assumptions	17	Model Assumptions	Model Assumptions
Characterizing heterogeneity	18	Not reported	Not reported
Characterizing distributional effects	19	Not reported	Not reported
Characterizing uncertainty	20	Model Outputs	Sensitivity Analyses
Approach to engagement with patients and others affected by the study	21	N/A	N/A
**Results**			

Study parameters	22	Supplemental	Table 1
Summary of main results	23	Cost-Effectiveness Analyses	Base Case Analysis, Table 2
Effect of uncertainty	24	Cost-Effectiveness Analyses	Sensitivity Analyses
Effect of engagement with patients and others affected by the study	25	N/A	N/A
**Discussion**			
Study findings, limitations, generalizability, and current knowledge	26	Discussion	Discussion
**OTHER RELEVANT INFORMATION**			
Source of funding	27	Acknowledgements	Financial disclosure
Conflicts of interest	28	Disclosures	Financial disclosure

Section item	Item	Wakase et al (2021)	Wang et al (2021)
**Title**			
Title	1	Front page	Front page
**Abstract**			
Abstract	2	Front page	Front page
**Introduction**			
Background and objectives	3	Introduction	Introduction
**Methods**			
Health economic analysis plan	4	Not reported	Not reported
Study population	5	Introduction	Introduction
Setting and location	6	Introduction	Introduction
Comparators	7	Model Overview and Structure	Model Overview and Structure
Perspective	8	Model Overview and Structure	Model Overview and Structure
Time horizon	9	Model Overview and Structure	Model Overview and Structure
Discount rate	10	Model Overview and Structure	Model Overview and Structure
Selection of outcomes	11	Model Outputs	Model Overview and Structure
Measurement of outcomes	12	Model Outputs	Model Overview and Structure
Valuation of outcomes	13	Utility inputs	Utility inputs
Measurement and valuation of resources and costs	14	Table 2	Resource use and cost inputs, Table 1 and Table 2

Currency, price date, and conversion	15	Cost Inputs	Model Overview and Structure
Rationale and description of model	16	Model Overview and Structure	Model Overview and Structure
Analytics and assumptions	17	Model Assumptions	Not reported
Characterizing heterogeneity	18	Not reported	Not reported
Characterizing distributional effects	19	Not reported	Not reported
Characterizing uncertainty	20	Model Outputs	Sensitivity analyses
Approach to engagement with patients and others affected by the study	21	N/A	N/A
**Results**			
Study parameters	22	Table 2	Table 1 and Table 2
Summary of main results	23	Base Case Analysis, Table 2	Base-case analysis, Table 4
Effect of uncertainty	24	Probabilistic sensitivity analyses	Sensitivity analyses
Effect of engagement with patients and others affected by the study	25	N/A	N/A
**Discussion**			
Study findings, limitations, generalizability, and current knowledge	26	Discussion	Discussion
**OTHER RELEVANT INFORMATION**			
Source of funding	27	Acknowledgements	Declaration of funding
Conflicts of interest	28	Conflict of interest statement	Declaration of financial/other interests

**Section item**	**Item**	**Joyner et al (2022)**	**Bastos-Oreiro (2022)**
**Title**			
Title	1	Front page	Front page
**Abstract**			
Abstract	2	Front page	Front page
**Introduction**			
Background and objectives	3	Introduction	Introduction
**Methods**			
Health economic analysis plan	4	Not reported	Not reported
Study population	5	Data	Population
Setting and location	6	Data	Model Structure

Comparators	7	Structure	Treatment Strategies
Perspective	8	Key Summary Points	Base Case
Time horizon	9	Structure	Base Case
Discount rate	10	Table 1	Model Structure
Selection of outcomes	11	Introduction	Outcomes
Measurement of outcomes	12	Introduction	Outcomes
Valuation of outcomes	13	Introduction	Utilities
Measurement and valuation of resources and costs	14	Table 1 and Table 2	Model Structure
Currency, price date, and conversion	15	Cost Estimation	Resource Use and Costs
Rationale and description of model	16	Structure	Model Structure
Analytics and assumptions	17	Not reported	Not reported
Characterizing heterogeneity	18	Not reported	Not reported
Characterizing distributional effects	19	Not reported	Not reported
Characterizing uncertainty	20	Results Presentation	Sensitivity Analysis
Approach to engagement with patients and others affected by the study	21	N/A	N/A
**Results**			
Study parameters	22	CAR T Cell Inputs and General, Model Inputs	Not reported
Summary of main results	23	Results	Base Case, Table 1
Effect of uncertainty	24	Results	Sensitivity Analysis
Effect of engagement with patients and others affected by the study	25	N/A	N/A
**Discussion**			
Study findings, limitations, generalizability, and current knowledge	26	Discussion	Discussion
**OTHER RELEVANT INFORMATION**			
Source of funding	27	Funding	Funding
Conflicts of interest	28	Disclosures	Conflicts of Interest

**Section item**	**Item**	**Li et al (2022)**	**Perales et al (2022)**
**Title**			
Title	1	Front page	Front page

**Abstract**			
Abstract	2	Front page	Front page
**Introduction**			
Background and objectives	3	Introduction	Introduction
**Methods**			
Health economic analysis plan	4	Not reported	Not reported
Study population	5	Population and interventions	Cost-Effectiveness Model Overview
Setting and location	6	Population and interventions	Cost-Effectiveness Model Overview
Comparators	7	Model structure	Introduction
Perspective	8	Introduction	Cost-Effectiveness Model Overview
Time horizon	9	Statistical analysis	Cost-Effectiveness Model Overview
Discount rate	10	Statistical analysis	Cost-Effectiveness Model Overview
Selection of outcomes	11	Effectiveness	Cost-Effectiveness Model Overview
Measurement of outcomes	12	Effectiveness	Cost-Effectiveness Model Overview
Valuation of outcomes	13	Effectiveness	Health-Related Quality of Life
Measurement and valuation of resources and costs	14	Costs, Table 1	Costs
Currency, price date, and conversion	15	Statistical analysis	Not reported
Rationale and description of model	16	Model structure	Cost-Effectiveness Model Overview
Analytics and assumptions	17	Not reported	Supplemental
Characterizing heterogeneity	18	Not reported	Not reported
Characterizing distributional effects	19	Not reported	Not reported
Characterizing uncertainty	20	Sensitivity analysis and scenario analyses	Sensitivity and Scenario Analyses
Approach to engagement with patients and others affected by the study	21	N/A	N/A
**Results**			
Study parameters	22	Table 1	Table 1
Summary of main results	23	Base case analysis	Cost-Effectiveness Analysis
Effect of uncertainty	24	Results	Cost-Effectiveness Analysis
Effect of engagement with patients and others affected by the study	25	N/A	N/A

**Discussion**			
Study findings, limitations, generalizability, and current knowledge	26	Discussion	Discussion
**OTHER RELEVANT INFORMATION**			
Source of funding	27	Funding	Financial disclosure
Conflicts of interest	28	Conflict of interest	Conflict of interest statement

**Section item**	**Item**	**Hillis et al (2022)**	**Oluwale et al (2022)**
**Title**			
Title	1	Front page	Front page
**Abstract**			
Abstract	2	Front page	Front page
**Introduction**			
Background and objectives	3	Introduction	Introduction
**Methods**			
Health economic analysis plan	4	Not reported	Not reported
Study population	5	Target Population	Model overview
Setting and location	6	Target Population	Model overview
Comparators	7	Comparators	Introduction
Perspective	8	Model Perspective	Model overview
Time horizon	9	Time Horizon	Model overview
Discount rate	10	Methods	Model overview
Selection of outcomes	11	Table 1	Model overview
Measurement of outcomes	12	Table 1	Model overview
Valuation of outcomes	13	Utility Values	Model overview
Measurement and valuation of resources and costs	14	Supplemental	Model overview
Currency, price date, and conversion	15	Cost and Resource Use	Model overview
Rationale and description of model	16	Model Structure and Approach	Model overview
Analytics and assumptions	17	Validation	Model overview
Characterizing heterogeneity	18	Not reported	Not reported
Characterizing distributional effects	19	Not reported	Not reported

Characterizing uncertainty	20	Analyses	Model overview
Approach to engagement with patients and others affected by the study	21	N/A	N/A
**Results**			
Study parameters	22	Supplemental	Table 1
Summary of main results	23	Deterministic Analysis, Table 3	Base case, Table 2
Effect of uncertainty	24	Probabilistic Sensitivity Analysis	Univariate and probabilistic sensitivity analyses
Effect of engagement with patients and others affected by the study	25	N/A	N/A
**Discussion**			
Study findings, limitations, generalizability, and current knowledge	26	Discussion	Discussion
**OTHER RELEVANT INFORMATION**			
Source of funding	27	Funding	Declaration of funding
Conflicts of interest	28	Conflict of interest	Declaration of financial/other interests

Section item	Item	Petersohn et al (2022)	Shah et al (2022)
**Title**			
Title	1	Front page	Front page
**Abstract**			
Abstract	2	Front page	Front page
**Introduction**			
Background and objectives	3	Introduction	Introduction
**Methods**			
Health economic analysis plan	4	Not reported	Not reported
Study population	5	Supplemental	Patient Population
Setting and location	6	Survival	Model Overview
Comparators	7	Overview	Model Overview
Perspective	8	Overview	Model Overview
Time horizon	9	Overview	Model Overview
Discount rate	10	Overview	Model Overview
Selection of outcomes	11	Outcomes	Outcomes

Measurement of outcomes	12	Outcomes	Outcomes
Valuation of outcomes	13	Utilities	Health State Utilities
Measurement and valuation of resources and costs	14	Cost and resource use	Cost and Resource Use
Currency, price date, and conversion	15	Overview	Model Overview
Rationale and description of model	16	Model Structure	Model Structure
Analytics and assumptions	17	Survival, Cost and resource use and Utilities	Supplemental
Characterizing heterogeneity	18	Not reported	Not reported
Characterizing distributional effects	19	Not reported	Not reported
Characterizing uncertainty	20	Uncertainty analyses	Sensitivity Analyses
Approach to engagement with patients and others affected by the study	21	N/A	N/A
**Results**			
Study parameters	22	Table 1	Table 1
Summary of main results	23	Base case results, Table 2	Overall Population
Effect of uncertainty	24	Uncertainty analyses	Sensitivity Analyses
Effect of engagement with patients and others affected by the study	25	N/A	N/A
**Discussion**			
Study findings, limitations, generalizability, and current knowledge	26	Discussion	Discussion
**OTHER RELEVANT INFORMATION**			
Source of funding	27	Declaration of funding	Funding
Conflicts of interest	28	Declaration of financial/other interests	Disclosures

**Section item**	**Item**	**Carey et al (2022)**	**Gye et al (2022)**
**Title**			
Title	1	Front page	Front page
**Abstract**			
Abstract	2	Front page	Front page
**Introduction**			
Background and objectives	3	Aim	Introduction and Intervention and Comparator
**Methods**			

Health economic analysis plan	4	Not reported	Not reported
Study population	5	Population	Study Setting and Patient Population
Setting and location	6	Model Structure	Study Setting and Patient Population
Comparators	7	Comparator	Intervention and Comparator
Perspective	8	Perspective	Study Setting and Patient Population
Time horizon	9	Model Structure	Model Structure
Discount rate	10	Model Structure	Model Structure
Selection of outcomes	11	Model Outputs	Outcome Measures
Measurement of outcomes	12	Model Outputs	Outcome Measures
Valuation of outcomes	13	Utility Inputs	Utility Values
Measurement and valuation of resources and costs	14	Cost Inputs	Resource Use and Costs
Currency, price date, and conversion	15	Cost Inputs	Resource Use and Costs
Rationale and description of model	16	Model Structure	Model Structure
Analytics and assumptions	17	Supplemental	Supplemental
Characterizing heterogeneity	18	Not reported	Not reported
Characterizing distributional effects	19	Not reported	Not reported
Characterizing uncertainty	20	Probabilistic ICER and Scatterplot	Sensitivity Analyses
Approach to engagement with patients and others affected by the study	21	N/A	N/A
**Results**			
Study parameters	22	Table 2	Table 1
Summary of main results	23	Deterministic Results	Base Case, Table 2
Effect of uncertainty	24	Probabilistic Results	Sensitivity Analyses
Effect of engagement with patients and others affected by the study	25	N/A	N/A
**Discussion**			
Study findings, limitations, generalizability, and current knowledge	26	Discussion	Discussion
**OTHER RELEVANT INFORMATION**			
Source of funding	27	Not reported	Funding
Conflicts of interest	28	Conflicts of Interest	Conflict of interest

Section item	Item	Wang et al (2022)	Kambhampati et al (2022)
**Title**			
Title	1	Front page	Front page
**Abstract**			
Abstract	2	Front page	Front page
**Introduction**			
Background and objectives	3	Introduction	Introduction
**Methods**			
Health economic analysis plan	4	Not reported	Not reported
Study population	5	Introduction	Assumptions
Setting and location	6	Introduction	Assumptions
Comparators	7	Introduction	Introduction
Perspective	8	Introduction	Analysis
Time horizon	9	Model Outline	Model Structure
Discount rate	10	Model Outline	Analysis
Selection of outcomes	11	Model Outline	Analysis
Measurement of outcomes	12	Model Outline	Analysis
Valuation of outcomes	13	Utility Inputs	Model Inputs, Supplemental
Measurement and valuation of resources and costs	14	Healthcare Resource Utilization and Costs	Model inputs
Currency, price date, and conversion	15	Model Outline	Analysis
Rationale and description of model	16	Healthcare Resource Utilization and Costs	Model Structure and Supplemental
Analytics and assumptions	17	Model Outline	Assumptions
Characterizing heterogeneity	18	Not reported	Not reported
Characterizing distributional effects	19	Not reported	Not reported
Characterizing uncertainty	20	Sensitivity Analyses	Sensitivity analyses
Approach to engagement with patients and others affected by the study	21	N/A	N/A
**Results**			
Study parameters	22	Table 3-4	Table 1
Summary of main results	23	Base-Case Analysis, Table 6	Modelling second-line CAR-T in primary refractory/ early relapse DLBCL patients

Effect of uncertainty	24	Sensitivity Analyses, Table 5	Sensitivity analyses
Effect of engagement with patients and others affected by the study	25	N/A	N/A
**Discussion**			
Study findings, limitations, generalizability, and current knowledge	26	Discussion	Discussion
**OTHER RELEVANT INFORMATION**			
Source of funding	27	Funding	Conflict of interest disclosure
Conflicts of interest	28	Disclosure	Conflict of interest disclosure

**Section item**	**Item**	**Choe et al (2022)**	**Vijenthira et al (2022**
**Title**			
Title	1	Front page	Front page
**Abstract**			
Abstract	2	Front page	Front page
**Introduction**			
Background and objectives	3	Introduction	Background
**Methods**			
Health economic analysis plan	4	Not reported	Not reported
Study population	5	Treatment Strategies	Patients and Intervention
Setting and location	6	Model Parameters	Patients and Intervention
Comparators	7	Treatment Strategies	Patients and Intervention
Perspective	8	Introduction	Background
Time horizon	9	Model Structure	Model Design
Discount rate	10	Model Structure	Model Design
Selection of outcomes	11	Model Structure	Outcomes
Measurement of outcomes	12	Model Structure	Outcomes
Valuation of outcomes	13	Model Parameters	Utilities
Measurement and valuation of resources and costs	14	Supplemental	Costs
Currency, price date, and conversion	15	Model Structure	Costs and Supplement
Rationale and description of model	16	Model Structure	Model Design

Analytics and assumptions	17	Supplemental	Sensitivity Analysis
Characterizing heterogeneity	18	Not reported	Not reported
Characterizing distributional effects	19	Not reported	Not reported
Characterizing uncertainty	20	Sensitivity Analysis	Sensitivity Analysis
Approach to engagement with patients and others affected by the study	21	N/A	N/A
**Results**			
Study parameters	22	Table 1	Table 2
Summary of main results	23	Second-line CAR T Cell Therapy, Table 2	Base Case Analysis
Effect of uncertainty	24	Sensitivity Analyses	Sensitivity Analyses
Effect of engagement with patients and others affected by the study	25	N/A	N/A
**Discussion**			
Study findings, limitations, generalizability, and current knowledge	26	Discussion	Discussion
**OTHER RELEVANT INFORMATION**			
Source of funding	27	Funding/Support	Research Funding
Conflicts of interest	28	Conflict of Interest Disclosures	Authors' disclosures of potential conflicts of interest

**Section item**	**Item**	**Wu et al (2023)**	**Wu et al (2023)**
**Title**			
Title	1	Front page	Front page
**Abstract**			
Abstract	2	Front page	Front page
**Introduction**			
Background and objectives	3	Introduction	Introduction
**Methods**			
Health economic analysis plan	4	Not reported	Not reported
Study population	5	Model Structure	Patients and Intervention
Setting and location	6	Model Structure	Patients and Intervention
Comparators	7	Comparator	Patients and Intervention

Perspective	8	Introduction	Introduction
Time horizon	9	Main outcome	Model Design
Discount rate	10	Main outcome	Main Outcomes
Selection of outcomes	11	Main outcome	Main Outcomes
Measurement of outcomes	12	Main outcome	Main Outcomes
Valuation of outcomes	13	Utility	Costs and Utility
Measurement and valuation of resources and costs	14	Costs	Costs and Utility
Currency, price date, and conversion	15	Main outcome	Not reported
Rationale and description of model	16	Model structure	Model Design
Analytics and assumptions	17	Model structure, Costs, Utility	Supplemental
Characterizing heterogeneity	18	Not reported	Not reported
Characterizing distributional effects	19	Not reported	Not reported
Characterizing uncertainty	20	Sensitivity analyses	Sensitivity Analyses
Approach to engagement with patients and others affected by the study	21	N/A	N/A
**Results**			
Study parameters	22	Table 1	Table 1 and Table 2
Summary of main results	23	Base case analysis	First-Line Settings, Second-Line Settings, Third-Line or Later Settings
Effect of uncertainty	24	Sensitivity analyses	Sensitivity Analyses
Effect of engagement with patients and others affected by the study	25	N/A	N/A
**Discussion**			
Study findings, limitations, generalizability, and current knowledge	26	Discussion	Discussion
**OTHER RELEVANT INFORMATION**			
Source of funding	27	Declaration of funding	Funding
Conflicts of interest	28	Declaration of financial/other interests	Conflict of interest

Section item	Item	Kapinos et al (2023)	Karampampa et al (2023)
**Title**			
Title	1	Front page	Front page

**Abstract**			
Abstract	2	Front page	Front page
**Introduction**			
Background and objectives	3	Introduction	Overview
**Methods**			
Health economic analysis plan	4	Not reported	Not reported
Study population	5	Introduction	Overview
Setting and location	6	Introduction	Overview
Comparators	7	Introduction	Overview
Perspective	8	Microsimulation Modeling	Overview
Time horizon	9	Microsimulation Modeling	Overview
Discount rate	10	Microsimulation Modeling	Overview
Selection of outcomes	11	Introduction	Outcomes
Measurement of outcomes	12	Microsimulation Modeling	Outcomes
Valuation of outcomes	13	Microsimulation Modeling	Health state utilities
Measurement and valuation of resources and costs	14	Model Structure	Cost inputs
Currency, price date, and conversion	15	Microsimulation Modeling	Overview
Rationale and description of model	16	Model Structure	Model Structure
Analytics and assumptions	17	Microsimulation Modeling	Supplemental
Characterizing heterogeneity	18	Not reported	Not reported
Characterizing distributional effects	19	Not reported	Not reported
Characterizing uncertainty	20	Sensitivity Analyses	Sensitivity analyses
Approach to engagement with patients and others affected by the study	21	N/A	N/A
**Results**			
Study parameters	22	Table 1	Supplemental
Summary of main results	23	Results	Base case, Table 3 and Table 4
Effect of uncertainty	24	Results	Probabilistic sensitivity analysis results
Effect of engagement with patients and others affected by the study	25	N/A	N/A
**Discussion**			

Study findings, limitations, generalizability, and current knowledge	26	Discussion	Discussion
**OTHER RELEVANT INFORMATION**			
Source of funding	27	Funding	Declaration of funding
Conflicts of interest	28	Declaration of Conflicting Interests	Declaration of financial/other relationships

**Section item**	**Item**	**Potnis et al (2023)**	**Parker et al (2023)**
**Title**			
Title	1	Front page	Front page
**Abstract**			
Abstract	2	Front page	Front page
**Introduction**			
Background and objectives	3	Introduction	Overview
**Methods**			
Health economic analysis plan	4	Not reported	Not reported
Study population	5	Patients and intervention	Overview
Setting and location	6	Model construction	Overview
Comparators	7	Model construction	Overview
Perspective	8	Patients and intervention	Introduction
Time horizon	9	Patients and intervention	Overview
Discount rate	10	Model construction	Overview
Selection of outcomes	11	Model construction	Overview
Measurement of outcomes	12	Model construction	Analyses
Valuation of outcomes	13	Utilities	Utilities
Measurement and valuation of resources and costs	14	Costs	Resource Use and Costs
Currency, price date, and conversion	15	Model construction	Resource Use and Costs
Rationale and description of model	16	Model construction	Model Structure
Analytics and assumptions	17	Not reported	Model Verification and Validation
Characterizing heterogeneity	18	Not reported	Not reported
Characterizing distributional effects	19	Not reported	Not reported

Characterizing uncertainty	20	Sensitivity analyses	Analyses
Approach to engagement with patients and others affected by the study	21	N/A	N/A
**Results**			
Study parameters	22	Table 1	Table 3
Summary of main results	23	Base-case analysis, Table 3	Base-Case Analysis
Effect of uncertainty	24	Sensitivity analyses	PSA
Effect of engagement with patients and others affected by the study	25	N/A	N/A
**Discussion**			
Study findings, limitations, generalizability, and current knowledge	26	Discussion	Discussion
**OTHER RELEVANT INFORMATION**			
Source of funding	27	Authorship	Funding
Conflicts of interest	28	Conflict of interest disclosure	Disclosures

Section item	Item	Loftager et al (2023)	Lin et al (2023)
**Title**			
Title	1	Front page	Front page
**Abstract**			
Abstract	2	Front page	Front page
**Introduction**			
Background and objectives	3	Introduction	Introduction
**Methods**			
Health economic analysis plan	4	Not reported	Not reported
Study population	5	Introduction	Population, Interventions and Outcomes
Setting and location	6	Introduction	Introduction
Comparators	7	Introduction	Population, Interventions and Outcomes
Perspective	8	Introduction	Introduction
Time horizon	9	Model overview	Model Structure
Discount rate	10	Model overview	Population, Interventions and Outcomes
Selection of outcomes	11	Model overview	Population, Interventions and Outcomes

Measurement of outcomes	12	Model overview	Population, Interventions and Outcomes
Valuation of outcomes	13	Health-related quality of life	Health utility values
Measurement and valuation of resources and costs	14	Costs	Medical costs and health resources used
Currency, price date, and conversion	15	Treatment costs	Medical costs and health resources used
Rationale and description of model	16	Model overview	Model Structure
Analytics and assumptions	17	Post-event utilities, Health state costs and Sensitivity and scenario analyses	Not reported
Characterizing heterogeneity	18	Not reported	Not reported
Characterizing distributional effects	19	Not reported	Not reported
Characterizing uncertainty	20	Sensitivity and scenario analyses	Sensitivity and Scenario Analyses
Approach to engagement with patients and others affected by the study	21	N/A	N/A
**Results**			
Study parameters	22	Costs	Table 1
Summary of main results	23	Cost-effectiveness analysis, Table 5	Base Case Analysis
Effect of uncertainty	24	Sensitivity and Scenario Analyses	Sensitivity Analysis
Effect of engagement with patients and others affected by the study	25	N/A	N/A
**Discussion**			
Study findings, limitations, generalizability, and current knowledge	26	Discussion	Discussion
**OTHER RELEVANT INFORMATION**			
Source of funding	27	Funding	Funding/Support:
Conflicts of interest	28	Declaration of financial/other relationships	Conflict of Interest Disclosures

Section item	Item	Kelker et al (2023)	Marchetti (2023)
**Title**			
Title	1	Front page	Front page
**Abstract**			
Abstract	2	Front page	Front page
**Introduction**			
Background and objectives	3	Introduction	Introduction

**Methods**			
Health economic analysis plan	4	Not reported	Not reported
Study population	5	Data	Methods
Setting and location	6	Model Conceptualization	Introduction
Comparators	7	Methods	Not reported
Perspective	8	Costs	Methods
Time horizon	9	Model Conceptualization	Methods
Discount rate	10	Discounting	Methods
Selection of outcomes	11	Effectiveness	Methods
Measurement of outcomes	12	Effectiveness	Methods
Valuation of outcomes	13	Quality of Life	Methods
Measurement and valuation of resources and costs	14	Costs	Methods
Currency, price date, and conversion	15	Costs	Methods
Rationale and description of model	16	Methods	Methods
Analytics and assumptions	17	Validation	Methods
Characterizing heterogeneity	18	Not reported	Not reported
Characterizing distributional effects	19	Not reported	Not reported
Characterizing uncertainty	20	Sensitivity and Scenario Analyses	Methods
Approach to engagement with patients and others affected by the study	21	N/A	N/A
**Results**			
Study parameters	22	Table 1	Not reported
Summary of main results	23	Axi-cel Versus ASCT, Liso-cel Versus ASCT, Table 2	Results, Table 2 and Table 3
Effect of uncertainty	24	Sensitivity and Scenario Analyses	Results
Effect of engagement with patients and others affected by the study	25	N/A	N/A
**Discussion**			
Study findings, limitations, generalizability, and current knowledge	26	Discussion	Discussion
**OTHER RELEVANT INFORMATION**			
Source of funding	27	Primary Funding Source	Funding

Conflicts of interest	28	Disclosures	Disclosure statement

**Section item**	**Item**	**Oluwale et al (2023)**	**Yamamoto et al (2024)**
**Title**			
Title	1	Front page	Front page
**Abstract**			
Abstract	2	Front page	Front page
**Introduction**			
Background and objectives	3	Introduction	Introduction
**Methods**			
Health economic analysis plan	4	Not reported	Not reported
Study population	5	Model updates	Model Construction
Setting and location	6	Not reported	Model Construction
Comparators	7	Introduction	Introduction
Perspective	8	Analysis	Costs and Effectiveness
Time horizon	9	Analysis	Introduction
Discount rate	10	Analysis	Model Construction
Selection of outcomes	11	Analysis	Model Construction
Measurement of outcomes	12	Analysis	Model Construction
Valuation of outcomes	13	Table 1	Costs and Effectiveness
Measurement and valuation of resources and costs	14	Not reported	Costs and Effectiveness, Supplemental
Currency, price date, and conversion	15	Model updates	Model Construction
Rationale and description of model	16	Model updates	Model Construction
Analytics and assumptions	17	Not reported	Supplemental
Characterizing heterogeneity	18	Not reported	Not reported
Characterizing distributional effects	19	Not reported	Not reported
Characterizing uncertainty	20	Scenario analyses, Supplemental	Sensitivity Analysis
Approach to engagement with patients and others affected by the study	21	N/A	N/A
**Results**			
Study parameters	22	Table 1	Table 2

Summary of main results	23	Results, Table 2	Base Case Analysis, Table 3 and Table 4
Effect of uncertainty	24	Results	Sensitivity Analysis
Effect of engagement with patients and others affected by the study	25	N/A	N/A
**Discussion**			
Study findings, limitations, generalizability, and current knowledge	26	Discussion	Discussion
**OTHER RELEVANT INFORMATION**			
Source of funding	27	Declaration of funding	Financial disclosure
Conflicts of interest	28	Declaration of financial/other interests	Conflict of interest statement

**Section item**	**Item**	**Choe et al (2024)**	**Overall Score (%)**
**Title**			
Title	1	Front page	100
**Abstract**			
Abstract	2	Front page	100
**Introduction**			
Background and objectives	3	Introduction	100
**Methods**			
Health economic analysis plan	4	Not reported	0
Study population	5	Treatment strategies	100
Setting and location	6	Treatment strategies	98
Comparators	7	Treatment strategies	98
Perspective	8	Introduction	100
Time horizon	9	Model structure and calibration	100
Discount rate	10	Model structure and calibration	100
Selection of outcomes	11	Treatment strategies	100
Measurement of outcomes	12	Treatment strategies	100
Valuation of outcomes	13	Model parameters	98
Measurement and valuation of resources and costs	14	Model parameters	98
Currency, price date, and conversion	15	Model parameters, Supplemental	93

Rationale and description of model	16	Model structure and calibration	100
Analytics and assumptions	17	Not reported	82
Characterizing heterogeneity	18	Not reported	0
Characterizing distributional effects	19	Not reported	0
Characterizing uncertainty	20	Sensitivity analyses	
Approach to engagement with patients and others affected by the study	21	N/A	N/A
**Results**			
Study parameters	22	Table 1	96
Summary of main results	23	Base case analysis, Table 2	100
Effect of uncertainty	24	Sensitivity analyses	98
Effect of engagement with patients and others affected by the study	25	N/A	N/A
**Discussion**			
Study findings, limitations, generalizability, and current knowledge	26	Discussion	100
**OTHER RELEVANT INFORMATION**			
Source of funding	27	Not reported	96
Conflicts of interest	28	Conflict of interest disclosure	98

References

1. Roth JA, Sullivan SD, Lin VW, Bansal A, Purdum AG, Navale L, et al. Cost-effectiveness of axicabtagene ciloleucel for adult patients with relapsed or refractory large B-cell lymphoma in the United States. J Med Econ. 2018;21(12):1238-45.
2. Whittington MD, McQueen RB, Ollendorf DA, Kumar VM, Chapman RH, Tice JA, et al. Long-term Survival and Cost-effectiveness Associated With Axicabtagene Ciloleucel vs Chemotherapy for Treatment of B-Cell Lymphoma. JAMA Network Open. 2019;2(2):e190035.
3. Lin JK, Muffly LS, Spinner MA, Barnes JI, Owens DK, Goldhaber-Fiebert JD. Cost Effectiveness of Chimeric Antigen Receptor T-Cell Therapy in Multiply Relapsed or Refractory Adult Large B-Cell Lymphoma. Journal of clinical oncology : official journal of the American Society of Clinical Oncology. 2019;37(24):2105-19.
4. Liu R, Oluwole OO, Diakite I, Botteman MF, Snider JT, Locke FL. Cost effectiveness of axicabtagene ciloleucel versus tisagenlecleucel for adult patients with relapsed or refractory large B-cell lymphoma after two or more lines of systemic therapy in the United States. Journal of medical economics. 2021;24(1):458-68.
5. Oluwole OO, Liu R, Diakite I, Feng C, Patel A, Nourhussein I, et al. Cost-effectiveness of axicabtagene ciloleucel versus lisocabtagene maraleucel for adult patients with relapsed or refractory large B-cell lymphoma after two or more lines of systemic therapy in the US. Journal of medical economics. 2022;25(1):541-51.
6. Cummings Joyner AK, Snider JT, Wade SW, Wang ST, Buessing MG, Johnson S, et al. Cost-Effectiveness of Chimeric Antigen Receptor T Cell Therapy in Patients with Relapsed or Refractory Large B Cell Lymphoma: No Impact of Site of Care. Adv Ther. 2022;39(8):3560-77.
7. Bastos-Oreiro M, de Las Heras A, Presa M, Casado MA, Pardo C, Martín-Escudero V, et al. Cost-Effectiveness Analysis of Axicabtagene Ciloleucel vs. Tisagenlecleucel for the Management of Relapsed/Refractory Diffuse Large B-Cell Lymphoma in Spain. Cancers. 2022;14(3).
8. Perales MA, Kuruvilla J, Snider JT, Vadgama S, Blissett R, El-Moustaid F, et al. The Cost-Effectiveness of Axicabtagene Ciloleucel as Second-Line Therapy in Patients with Large B-Cell Lymphoma in the United States: An Economic Evaluation of the ZUMA-7 Trial. Transplantation and Cellular Therapy. 2022.
9. Kambhampati S, Saumoy M, Schneider Y, Serrao S, Solaimani P, Budde LE, et al. Cost-effectiveness of second-line axicabtagene ciloleucel in relapsed refractory diffuse large B-cell lymphoma. Blood. 2022;140(19):2024-36.
10. Hillis C, Vicente C, Ball G. The Cost Effectiveness of Axicabtagene Ciloleucel Versus Best Supportive Care in the Treatment of Adult Patients with Relapsed or Refractory Large B-Cell Lymphoma (LBCL) After Two or More Lines of Systemic Therapy in Canada. Pharmacoeconomics. 2022;40(9):917-28.
11. Li. Cost-effectiveness analysis of axicabtagene ciloleucel vs. salvage chemotherapy for relapsed or refractory adult diffuse large B-cell lymphoma in China. 2022.
12. Potnis KC, Di M, Isufi I, Gowda L, Seropian SE, Foss FM, et al. Cost-effectiveness of chimeric antigen receptor T-cell therapy in adults with relapsed or refractory follicular lymphoma. Blood advances. 2023;7(5):801-10.
13. Loftager ASL, Danø A, Eklund O, Vadgama S, Hedlof Kanje V, Munk E. Axicabtagene ciloleucel compared to standard of care in Swedish patients with large B-cell lymphoma: a cost-effectiveness analysis of the ZUMA-7 trial. Journal of medical economics. 2023;26(1):1303-17.
14. Choe JH, Abdel-Azim H, Padula WV, Abou-El-Enein M. Cost-effectiveness of Axicabtagene Ciloleucel and Tisagenlecleucel as Second-line or Later Therapy in Relapsed or Refractory Diffuse Large B-Cell Lymphoma. JAMA Netw Open. 2022;5(12):e2245956.
15. Wu W, Zhou Y, Wang Y, Keramat SA, Balasooriya NN, Zhao Z, et al. Value for Money of CAR-T Cell Therapy for Patients with Diffuse Large B-cell Lymphoma in China: Evidence from a Cost-Effectiveness Analysis. Applied health economics and health policy. 2023;21(5):773-83.
16. Vijenthira A, Kuruvilla J, Crump M, Jain M, Prica A. Cost-Effectiveness Analysis of Frontline Polatuzumab-Rituximab, Cyclophosphamide, Doxorubicin, and Prednisone and/or Second-Line Chimeric Antigen Receptor T-Cell Therapy Versus Standard of Care for Treatment of Patients With Intermediate- to High-Risk Diffuse Large B-Cell Lymphoma. Journal of clinical oncology : official journal of the American Society of Clinical Oncology. 2023;41(8):1577-89.

17. Oluwole OO, Patel AR, Vadgama S, Smith NJ, Blissett R, Feng C, et al. An updated cost-effectiveness analysis of axicabtagene ciloleucel in second-line large B-cell lymphoma patients in the United States. Journal of medical economics. 2024;27(1):77-83.
18. Whittington MD, McQueen RB, Ollendorf DA, Kumar VM, Chapman RH, Tice JA, et al. Long-term Survival and Value of Chimeric Antigen Receptor T-Cell Therapy for Pediatric Patients with Relapsed or Refractory Leukemia. JAMA Pediatrics. 2018;172(12):1161-8.
19. Lin JK, Lerman BJ, Barnes JI, Boursiquot BC, Tan YJ, Robinson AQL, et al. Cost effectiveness of chimeric antigen receptor T-cell therapy in relapsed or refractory pediatric B-cell acute lymphoblastic leukemia. J Clin Oncol. 2018;36(32):3192-202.
20. Sarkar RR, Gloude NJ, Schiff D, Murphy JD. Cost-Effectiveness of Chimeric Antigen Receptor T-Cell Therapy in Pediatric Relapsed/Refractory B-Cell Acute Lymphoblastic Leukemia. J Natl Cancer Inst. 2019;111(7):719-26.
21. Thielen FW, van Dongen-Leunis A, Arons AMM, Ladestein JR, Hoogerbrugge PM, Uyl-de Groot CA. Cost-effectiveness of Anti-CD19 chimeric antigen receptor T-Cell therapy in pediatric relapsed/refractory B-cell acute lymphoblastic leukemia. A societal view. European journal of haematology. 2020;105(2):203-15.
22. Cher BP, Gan KY, Aziz MIA, Lin L, Hwang WYK, Poon LM, et al. Cost utility analysis of tisagenlecleucel vs salvage chemotherapy in the treatment of relapsed/refractory diffuse large B-cell lymphoma from Singapore's healthcare system perspective. Journal of medical economics. 2020;23(11):1321-9.
23. Furzer J, Gupta S, Nathan PC, Schechter T, Pole JD, Krueger J, et al. Cost-effectiveness of Tisagenlecleucel vs Standard Care in High-risk Relapsed Pediatric Acute Lymphoblastic Leukemia in Canada. JAMA Oncology. 2020;6(3):393-401.
24. Ribera Santasusana JM, de Andrés Saldaña A, García-Muñoz N, Gostkorzewicz J, Martínez Llinàs D, Díaz de Heredia C. Cost-Effectiveness Analysis of Tisagenlecleucel in the Treatment of Relapsed or Refractory B-Cell Acute Lymphoblastic Leukaemia in Children and Young Adults in Spain. ClinicoEconomics and outcomes research : CEOR. 2020;12:253-64.
25. Qi CZV, Bollu. Hongbo, Yang. Anand, Dalal. Su, Zhang. Jie, Zhang. Cost-Effectiveness Analysis of Tisagenlecleucel for the Treatment of Patients With Relapsed or Refractory Diffuse Large B-Cell Lymphoma in the United States. Clinical Therapeutics. 2021;43(8):28.
26. Wakase S, Teshima T, Zhang J, Ma Q, Watanabe Y, Yang H, et al. Cost-Effectiveness Analysis of Tisagenlecleucel for the Treatment of Pediatric and Young Adult Patients with Relapsed or Refractory B Cell Acute Lymphoblastic Leukemia in Japan. Transplantation and Cellular Therapy. 2021;27(3):241.e1-.e11.
27. Wakase S, Teshima T, Zhang J, Ma Q, Fujita T, Yang H, et al. Cost Effectiveness Analysis of Tisagenlecleucel for the Treatment of Adult Patients with Relapsed or Refractory Diffuse Large B Cell Lymphoma in Japan. Transplantation and cellular therapy. 2021;27(6):506.e1-.e10.
28. Moradi-Lakeh M, Yaghoubi M, Seitz P, Javanbakht M, Brock E. Cost-Effectiveness of Tisagenlecleucel in Paediatric Acute Lymphoblastic Leukaemia (pALL) and Adult Diffuse Large B-Cell Lymphoma (DLBCL) in Switzerland. Adv Ther. 2021;38(6):3427-43.
29. Wang XJ, Wang YH, Li SC, Gkitzia C, Hwang WY, et al. Cost-effectiveness and budget impact analyses of tisagenlecleucel in adult patients with relapsed or refractory diffuse large B-cell lymphoma from Singapore's private insurance payer's perspective. J Med Econ. 2021;24(1):637-53.
30. Wang. Cost-Effectiveness and Budget Impact Analyses of Tisagenlecleucel in Pediatric and Young Adult Patients with Relapsed or Refractory B-Cell Acute Lymphoblastic Leukemia from the Singapore Healthcare System Perspective. 2022.
31. Carey N, Leahy J, Trela-Larsen L, McCullagh L, Barry M. Tisagenlecleucel for relapsed/refractory acute lymphoblastic leukemia in the Irish healthcare setting: cost-effectiveness and value of information analysis. Int J Technol Assess Health Care. 2022;38(1).
32. Gye A, Goodall S, De Abreu Lourenco R. Cost-effectiveness Analysis of Tisagenlecleucel Versus Blinatumomab in Children and Young Adults with Acute Lymphoblastic Leukemia: Partitioned Survival Model to Assess the Impact of an Outcome-Based Payment Arrangement. Pharmacoeconomics. 2022;21:21.
33. Simons CL, Malone D, Wang M, Maglinte GA, Inocencio T, Wade SW, et al. Cost-effectiveness for KTE-X19 CAR T therapy for adult patients with relapsed/refractory mantle cell lymphoma in the United States. Journal of medical economics. 2021;24(1):421-31.

34. Ball G, Lemieux C, Cameron D, Seftel MD. Cost-Effectiveness of Brexucabtagene Autoleucel versus Best Supportive Care for the Treatment of Relapsed/Refractory Mantle Cell Lymphoma following Treatment with a Bruton's Tyrosine Kinase Inhibitor in Canada. Curr Oncol. 2022;29(3):2021-45.
35. Shah BD, Smith NJ, Feng C, Jeyakumar S, Castaigne JG, Faghmous I, et al. Cost-Effectiveness of KTE-X19 for Adults with Relapsed/Refractory B-Cell Acute Lymphoblastic Leukemia in the United States. Advances in therapy. 2022;39(8):3678-95.
36. Petersohn S, Salles G, Wang M, Wu J, Hess G, et al. Cost-effectiveness analysis of KTE-X19 CAR T therapy versus real-world standard of care in patients with relapsed/refractory mantle cell lymphoma post BTKi in England. J Med Econ. 2022;25(1):730-40.
37. Marchetti M, Visco C. Cost-Effectiveness of brexucabtagene autoleucel for relapsed/refractory mantle cell lymphoma. Leukemia & lymphoma. 2023;64(8):1442-50.
38. Parker C, Liu FF, Deger KA, Franco-Villalobos C, Proskorovsky I, Keating SJ, et al. Cost-Effectiveness of Lisocabtagene Maraleucel Versus Axicabtagene Ciloleucel and Tisagenlecleucel in the Third-Line or Later Treatment Setting for Relapsed or Refractory Large B-cell Lymphoma in the United States. Advances in therapy. 2023;40(5):2355-74.
39. Kelkar AH, Cliff ERS, Jacobson CA, Abel GA, Dijk SW, Krijkamp EM, et al. Second-Line Chimeric Antigen Receptor T-Cell Therapy in Diffuse Large B-Cell Lymphoma : A Cost-Effectiveness Analysis. Annals of internal medicine. 2023;176(12):1625-37.
40. Choe JH, Yu T, Abramson JS, Abou-El-Enein M. Cost-effectiveness of second-line lisocabtagene maraleucel in relapsed or refractory diffuse large B-cell lymphoma. Blood advances. 2024;8(2):484-96.
41. Kapinos KA, Hu E, Trivedi J, Geethakumari PR, Kansagra A. Cost-Effectiveness Analysis of CAR T-Cell Therapies vs Antibody Drug Conjugates for Patients with Advanced Multiple Myeloma. Cancer control : journal of the Moffitt Cancer Center. 2023;30:10732748221142945.
42. Wu W, Ding S, Mingming Z, Yuping Z, Sun X, Zhao Z, et al. Cost effectiveness analysis of CAR-T cell therapy for patients with relapsed/refractory multiple myeloma in China. Journal of medical economics. 2023;26(1):701-9.
43. Karampampa K, Zhang W, Venkatachalam M, Cotte FE, Dhanda D. Cost-effectiveness of idecabtagene vicleucel compared with conventional care in triple-class exposed relapsed/refractory multiple myeloma patients in Canada and France. Journal of medical economics. 2023;26(1):243-53.
44. Yamamoto C, Minakata D, Yokoyama D, Furuki S, Noguchi A, Koyama S, et al. Cost-Effectiveness of Anti-BCMA Chimeric Antigen Receptor T Cell Therapy in Relapsed/Refractory Multiple Myeloma. Transplantation and cellular therapy. 2024;30(1):118.e1-.e15.
45. Lin Z, Zuo C, Jiang Y, Su W, Yao X, Man Y, et al. Cost-Effectiveness Analysis of Relmacabtagene Autoleucel for Relapsed or Refractory Large B-Cell Lymphoma in China. Value in health regional issues. 2023;37:41-8.
